# Vitamin and Mineral Supplementation During Pregnancy on Maternal, Birth, Child Health and Development Outcomes in Low- and Middle-Income Countries: A Systematic Review and Meta-Analysis

**DOI:** 10.3390/nu12020491

**Published:** 2020-02-14

**Authors:** Christina Oh, Emily C. Keats, Zulfiqar A. Bhutta

**Affiliations:** 1 Centre for Global Child Health, The Hospital for Sick Children, Toronto, ON M5G 0A4, Canada; christina.oh@sickkids.ca (C.O.); emily.keats@sickkids.ca (E.C.K.); 2 Centre of Excellence in Women and Child’s Health, Aga Khan University, Karachi 74800, Pakistan

**Keywords:** micronutrient supplementation, vitamin supplementation, pregnancy, developing countries

## Abstract

Almost two billion people are deficient in key vitamins and minerals, mostly women and children in low- and middle-income countries (LMICs). Deficiencies worsen during pregnancy due to increased energy and nutritional demands, causing adverse outcomes in mother and child, but could be mitigated by interventions like micronutrient supplementation. To our knowledge, this is the first systematic review that aimed to compile evidence from both efficacy and effectiveness trials, evaluating different supplementation interventions on maternal, birth, child health, and developmental outcomes. We evaluated randomized controlled trials and quasi-experimental studies published since 1995 in peer-reviewed and grey literature that assessed the effects of calcium, vitamin A, iron, vitamin D, and zinc supplementation compared to placebo/no treatment; iron-folic (IFA) supplementation compared to folic acid only; multiple micronutrient (MMN) supplementation compared to IFA; and lipid-based nutrient supplementation (LNS) compared to MMN supplementation. Seventy-two studies, which collectively involved 314 papers (451,723 women), were included. Meta-analyses showed improvement in several key birth outcomes, such as preterm birth, small-for-gestational age (SGA) and low birthweight with MMN supplementation, compared to IFA. MMN also improved child outcomes, including diarrhea incidence and retinol concentration, which are findings not previously reported. Across all comparisons, micronutrient supplementation had little to no effect on mortality (maternal, neonatal, perinatal, and infant) outcomes, which is consistent with other systematic reviews. IFA supplementation showed notable improvement in maternal anemia and the reduction in low birthweight, whereas LNS supplementation had no apparent effect on outcomes; further research that compares LNS and MMN supplementation could help understand differences with these commodities. For single micronutrient supplementation, improvements were noted in only a few outcomes, mainly pre-eclampsia/eclampsia (calcium), maternal anemia (iron), preterm births (vitamin D), and maternal serum zinc concentration (zinc). These findings highlight that micronutrient-specific supplementation should be tailored to specific groups or needs for maximum benefit. In addition, they further contribute to the ongoing discourse of choosing antenatal MMN over IFA as the standard of care in LMICs.

## 1. Introduction

### 1.1. Background

Micronutrient deficiencies are a key contributing factor to poor health and suboptimal development outcomes, and they especially affect women and children who reside in low- and middle-income countries (LMICs) [1,2]. Micronutrient deficiencies are defined as insufficient amounts of essential vitamins and minerals, which are obtained from the diet, to meet recommended daily allowances for proper health, growth and development [3]. They often result from diets that chronically lack diversity or proper and sufficient nutrients, and in some cases, from infections and/or chronic disease that inhibit proper nutrient absorption [4]. Considered one of the three tenets of the triple burden of malnutrition, also known as hidden hunger, micronutrient deficiencies affect approximately two billion people globally [5,6]. Left unaddressed, micronutrient deficiencies are of particular concern as they will threaten the survival and well-being of women of reproductive age (WRA) and their infants, and may put subsequent generations of children at risk due to the intergenerational transfer of malnutrition [5,7].

Micronutrient deficiencies are often exacerbated during pregnancy due to increased nutritional requirements and, in LMICs, often appear concurrently (deficiencies in two or more micronutrients) [1,8]. Prenatal iron deficiency is one common example with a high global prevalence of 19.2% (95% confidence interval (CI) 17.1–21.5%), while maternal vitamin A deficiency affects approximately 15.3% (95% CI 6.0–24.6%) of pregnant women [1]. Despite sparse population-level data on deficiencies, these estimates reflect the current state of health of the global maternal population. Repeated pregnancies and short inter-pregnancy levels are also known contributors to poor maternal micronutrient status [2].

Micronutrients are critical for optimal pregnancy outcomes and proper metabolic activities that support tissue growth and functioning in the developing fetus. As such, deficiencies result in a vast array of adverse health outcomes affecting both mother and baby. Anemia, commonly caused by iron deficiency, is associated with increased risks of maternal mortality, perinatal mortality and low birthweight [9,10,11]. Folate and iodine deficiencies are well known to severely impair fetal development, leading to neural tube defects (NTDs) and an increased risk of mental retardation and cretinism, respectively [12,13]. Insufficient calcium during pregnancy is linked to the development of hypertension, which is a leading cause of maternal mortality, morbidity, fetal growth restriction and preterm birth [14,15]. Similar to calcium, vitamin D deficiency can lead to pre-eclampsia, and subsequently increase the risks of preterm birth, small-for-gestational age (SGA) and perinatal mortality [16,17,18]. While the effects of maternal zinc deficiency are not well established, it has been suggested that maternal zinc supplementation can reduce preterm birth [19].

Maternal malnutrition not only negatively affects the mother and fetus during the period of pregnancy, but also manifests through intergenerational effects. It can significantly alter the short-term and long-term health and development outcomes in offspring, including growth, neurodevelopment and cognition, and cardio-metabolic, pulmonary and immune functions [20]. Poor nutritional status in mothers shortchanges a newborn’s chance to reach their fullest potential in growth and development in the short term and establishes a trajectory for chronic illness and other diseases in adolescence and adulthood. Poor fetal and infant health due to maternal malnutrition are associated with stunting that can be sustained into adulthood, chronic diseases relating to nutrition, lower educational attainment, reduced income, and even decreased birthweight in the subsequent generation [21].

### 1.2. Current Strategies and Interventions

Several strategies exist globally to address micronutrient malnutrition in women and children. Common strategies include diet diversification, biofortification of staple crops, large-scale, targeted, and home fortification [22]. Micronutrient supplementation is another common strategy, often used for short-term, preventive purposes targeting specific at-risk population groups [4]. Micronutrients are ingested in the forms of tablets or other vehicles (e.g., syrup, drops, capsules, powder, or food matrices) and bioconverted to their active form. Thus, supplementation is a recommended part of routine antenatal care to overcome complications associated with micronutrient deficiencies during pregnancy, and to support maternal health and fetal development. This review will focus on micronutrient supplementation interventions during pregnancy.

Currently, the World Health Organization (WHO) recommends daily iron and folic acid (IFA) supplementation with 30–60 mg of elemental iron and 400 µg folic acid [23]. In populations where anemia prevalence is less than 20%, or where side effects from daily supplementation are severe, the WHO recommends intermittent (once weekly) supplementation with 120 mg of elemental iron and 2800 µg folic acid instead [23]. The WHO also recommends daily calcium supplementation (1.5–2.9 g oral elemental calcium) in populations with low dietary calcium intake and daily or weekly vitamin A supplementation (up to 10,000 IU or 25,000 IU, respectively) where vitamin A deficiency is a severe public health problem [23]. Zinc supplementation is only recommended where rigorous research supports its provision, and vitamin D supplementation is not recommended for pregnant women to improve maternal and perinatal outcomes [23]. The National Academies of Sciences, Engineering, and Medicine provide recommended dietary allowances (RDA) for micronutrients and vitamins during pregnancy [24] (see Table 1), which may slightly differ from recommendations by the WHO.

To address the issue of multiple, concurrent micronutrient deficiencies, the United Nations Children’s Fund (UNICEF), United Nations University, and the WHO developed a multiple-micronutrient (MMN) tablet, called UNIMMAP. The MMN tablet provides the daily recommended intakes of vitamins A (800 µg), B1 (1.4 mg), B2 (1.4 mg), B6 (1.9 mg), B12 (2.6 mg), C (70 mg), D (200 IU) and E (10 mg), and niacin (18 mg), folic acid (400 µg), copper (2 mg), selenium (65 µg), and iodine (150 µg) with 30 mg of iron and 15 mg of zinc for pregnant women [25]. Comparing to the RDA for micronutrients and vitamins during pregnancy by the National Academies of Sciences, Engineering, and Medicine (Table 1), the UNIMMAP formulation is very similar with identical amounts for vitamins B1, B2, B3, B6 and B12, and slightly higher amounts of vitamin A, iron, zinc, copper, and selenium. The UNIMMAP formulation contains lower amounts, compared to the RDAs, for vitamins C, D, and E, and folic acid and iodine [24]. Similar tablets for supplementation have been developed on a case-by-case basis, and typically provide at least three micronutrients.

More recently, lipid-based nutrient supplements (LNS) have been used to address the adverse effects of micronutrient deficiencies in mothers. LNS supplements typically contain the same vitamins and minerals found in MMN supplements, with the added components of protein, essential fatty acids, and energy in the form of fats (such as vegetable fat, peanut/groundnut paste, milk powder and sugar) [26]. Table 1 highlights the RDA for protein, carbohydrates and fats for pregnant women of various age ranges [24]. Traditionally, lipid-based products, like Plumpy’nut, have also been used to treat severe acute malnutrition; however, they have since been adapted to be used also as a preventive therapy for undernutrition [26]. It is important to note that supplementation with MMN is not recommended by the WHO for pregnant women to improve maternal and perinatal outcomes. The WHO has indicated that more research needs to be conducted to determine which micronutrients improve maternal and perinatal outcomes. [23]. Further, the WHO has not yet issued any guidance for LNS [23].

Primary studies and meta-analyses of randomized controlled trials (RCTs) have shown that some antenatal micronutrient supplementation interventions are efficacious in improving congenital/birth outcomes, including reduced risks of NTDs, cretinism, premature rupture of membranes (PROM), low birthweight and preterm birth [9,12,18,19,27,28,29,30]. It is important to consider that different supplements may require different timing of initiation and durations of exposure to produce clinically meaningful results. Folic acid supplementation is recommended as early as possible, ideally before conception [23], while daily iron supplementation at mid-gestation has been shown to be effective in improving health and development outcomes [31].

### 1.3. Why it is Important to Do this Review

Several existing systematic reviews—many of which include trials conducted in LMICs—have examined the impact of single and multiple micronutrient supplementation interventions in pregnancy [18,19,31,32,33,34,35,36,37,38,39,40,41,42,43]. However, among the reported results, significant heterogeneity exists; this has not yet been explained by subgroup analysis. Further, for several micronutrient supplementation interventions, such as folic acid supplementation for maternal health outcomes and calcium supplementation for pregnancy and infant outcomes, there are inconclusive results that necessitate further investigation. Recently, concerns have been raised regarding the safety of iron supplementation in women with high hemoglobin concentrations, and the potentially negative long-term consequences that unabsorbed iron may have on child morbidity [44,45]. Moreover, many of the systematic reviews are several years old, highlighting the need for current and updated evidence, including newly completed trial data. This is especially important given the constantly changing nutrition landscape globally and the growing triple burden of obesity, malnutrition and micronutrient deficiency. Lastly, there is a gap in research examining the effectiveness of antenatal micronutrient supplementation interventions in a real world setting.

The objective of this systematic review is to understand which antenatal supplementation interventions are effective at improving key maternal and child health, nutrition and mortality outcomes in LMICs, including data from large programme evaluations, as well as, smaller studies. Another aim is to conduct subgroup analyses to answer questions regarding potential differences in outcomes based on maternal/child and intervention characteristics.

## 2. Materials and Methods

### 2.1. Literature Search

We used the PICO methodology [46] to formulate our search strategy (see Supplementary Materials, Table S1) based on medical subject headings (MeSH) and key words. Both grey literature and databases were searched. Databases included CAB Abstracts, CINAHL, Cochrane Library, Embase, International Initiative for Impact Evaluations, LILACS, Medline, Popline, Web Science and WHO library database. Non-indexed, grey literature searches of organizational websites were also conducted to locate relevant studies. The date of the final search was 31 October 2019.

### 2.2. Study Selection and Data Abstraction

Studies for title and abstract screening and full-text screening were managed using Covidence, a streamlined, web-based software platform for systematic review processes. Titles and abstracts were screened independently. If eligibility could not be determined by screening the title alone, then the abstract was also screened. Full-text screening was conducted in duplicate, according to the same inclusion/exclusion criteria. Any disagreement was resolved by a third reviewer. Study eligibility criteria are summarized in Table 2.

Studies were eligible if they included data collected in or after 1995 on vitamin and mineral supplementation during pregnancy in healthy mothers of any age and parity in LMICs, which were defined based on the World Bank Group income classification at the time of the search. The year 1995 was used as a cut off to capture the best and most recent evidence on antenatal supplementation. There were no language restrictions. Studies were excluded if the maternal population was considered unhealthy (e.g., chronic and genetic diseases, metabolic and nutritional disorders). Though our aim was to include healthy pregnant women, we recognize that the prevalence of micronutrient deficiencies is high in LMICs, and that it is likely that women have one or more micronutrient deficiencies at baseline; women were not excluded on this basis. Eligible study designs included randomized controlled trials (RCTs) and quasi-experimental designs (including, natural experiments, controlled before/after studies, regression discontinuity designs and interrupted time series studies). Reviews were excluded, but reference lists were hand-searched for relevant studies. All data was extracted in duplicate, using a standardized, previously piloted data abstraction form that comprised of a general study information sheet and a quantitative outcome sheet.

### 2.3. Quality Assessment

Quality assessment of individual studies was conducted in duplicate. Individual studies were critically appraised using the Cochrane Risk of Bias (ROB) tool for RCTs and the Cochrane Effective Practice and Organization of Care (EPOC) guidelines for controlled before/after studies and interrupted time series studies. We considered the following domains: random sequence generation, allocation concealment, similar baseline outcome measurements, similar baseline characteristics, incomplete outcome data, knowledge of the allocated interventions adequately prevented during study, protection against contamination, selective outcome reporting and other risk of bias (e.g., bias in measurement). Any discrepancies were resolved through discussion and with a third reviewer.

### 2.4. Data Synthesis

Statistical analysis was conducted using Review Manager 5.3. A random effects meta-analysis was used to mitigate heterogeneity within included studies of a given synthesis. The generic inverse-variance approach was used for both dichotomous and continuous outcomes to adjust study weights according to the variance of the effect estimate. The GRADE tool was used to assess the quality of the evidence for the primary outcomes for which meta-analysis was conducted. The outcomes assessed with GRADE were maternal mortality, maternal anemia, low birthweight and perinatal mortality. Meta-analyses were conducted only where there were data for, at minimum, three studies per outcome of interest.

Subgroup analyses for primary outcomes, where ≥three studies per subgroup of interest were available, were conducted for the following variables: maternal age, geographical region (based on the WHO regions), duration of intervention, dose of the intervention for iron and iron-folic acid supplementation, the formulation for multiple micronutrient supplements, sex of infant, and baseline nutritional status in mothers (anemia status, nutritional status based on body mass index, low vs. normal stature).

An exploratory post-hoc analysis was also conducted for both primary and secondary outcomes provided available data, comparing studies that strictly gave the single micronutrient supplement and those that provided additional micronutrients (e.g., iron-folic acid) with the single micronutrient in question, or for MMN vs. IFA, MMN supplements that contained >4 components and those that contained three or four micronutrients. A post-hoc sensitivity analysis was also conducted based on studies that reported a conflict of interest. The methodology for this review is described in detail in the published protocol [47].

## 3. Results

**Figure 1:**
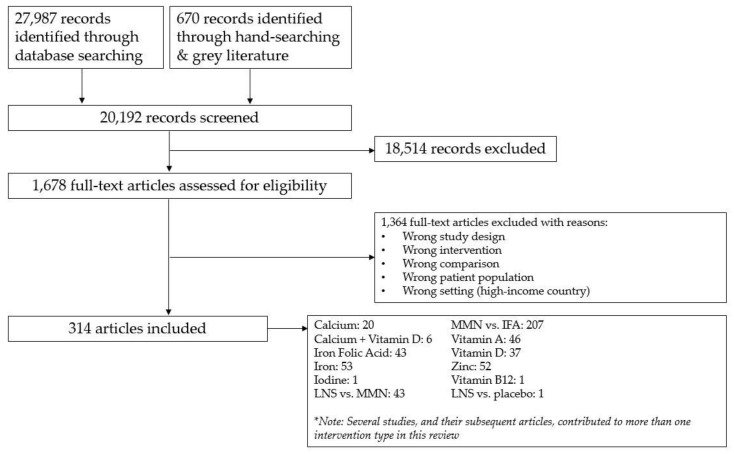
Study flow diagram.

From a database search of 27,987 citations and from 670 citations identified through hand-searching and a grey literature search, 1246 full-text articles and 432 full-text articles, respectively, were screened. Of these, a total of 72 unique studies, with 314 associated papers, were identified for inclusion based on our pre-defined inclusion and exclusion criteria (Figure 1). Eight studies were included in the review, but did not contribute data to the meta-analyses for reasons including incomplete data and no reported outcomes of interest [48,49,50,51,52,53,54,55]. Subgroup analyses were conducted for the following comparisons: zinc vs. placebo, iron vs. placebo, and MMN vs. IFA supplementation. Post-hoc subgroup analyses were conducted for all outcomes, across all comparisons when ≥three studies per subgroup of interest were available.

The majority of studies were conducted in four regions: East Asia and Pacific (*n*studies = 12; [50,53,56,57,58,59,60,61,62,63,64,65]), Middle-East and North Africa (*n* = 13; [54,66,67,68,69,70,71,72,73,74,75,76,77]), sub-Saharan Africa (*n* = 18; [51,78,79,80,81,82,83,84,85,86,87,88,89,90,91,92,93,94]) and South Asia (*n* = 19; [49,95,96,97,98,99,100,101,102,103,104,105,106,107,108,109,110,111,112]). Seven studies were conducted in Latin America and the Caribbean [48,113,114,115,116,117,118], and only one trial was conducted in Europe and Central Asia (Turkey; [52]). Two trials were multi-country studies [55,119], conducted in countries representative of multiple geographical regions. A summary of study characteristics can be found in the Supplementary Materials, Table S2.

Overall, the majority of included studies were deemed high quality. The risk of bias was generally low, with at least 75% of the judgements assessed as low risk for four domains (blinding of participants, personnel and outcome assessment, selective reporting and other biases), and at least 50% of the judgements as low risk for three domains of the quality assessment (random sequence generation, allocation concealment and incomplete outcome data).First com

The 64 studies included in data analyses enrolled 439,649 women of varying gestational age at baseline, ranging from early pregnancy (<13 weeks gestation) to <37 weeks gestation. Most participants were enrolled at mid-gestation at baseline (approximately 20 weeks gestational age). Inclusion criteria most studies were similar, requiring pregnant women who were healthy, without anemia or any other known chronic or systemic medical condition (e.g., cardiovascular diseases, tuberculosis, human immunodeficiency virus (HIV)) or heightened risk of pregnancy complications, such as a history of gestational hypertension, pre-eclampsia or eclampsia. Baseline characteristics of participants assigned to the intervention and control groups were overall comparable for the majority of studies, with the exception of five studies. In two studies, the control or placebo groups included participants that differed from those in the intervention group, in terms of land ownership and socio-economic status [93,98]. In the study by Friis et al., there were more primigravidae participants in the control group compared to the intervention group [83]. In the Roberfroid et al. study, the serum hemoglobin level of participants in the intervention group was lower than those in the control group at baseline [90]. Lastly, in the study by Ramakrishnan et al., in the intervention group, there was a greater proportion of single mothers, and participants with a low mean BMI in comparison to the control group [118]. Across all studies, supplements were given orally in the form of tablets, drops, syrup or powder and were given to participants from the time of enrollment until the end of pregnancy, the most common endpoint of the intervention.

### 3.1. Vitamin A Supplementation Versus Placebo

Nine studies assessed vitamin A supplementation compared to placebo or no treatment [53,60,63,79,86,92,94,110,111]. Of these, one study was excluded from meta-analysis because it reported no poolable outcomes, but was narratively synthesized [53] (see Supplementary Materials, Table S3). Thus, eight studies were included in the meta-analyses [60,63,79,86,92,94,110,111]. Four of these studies were conducted in the sub-Saharan Africa—of which, two were in Ghana [79,86], one in Malawi [92] and one in Tanzania [94]. From East Asia and the Pacific, two studies were conducted in Indonesia [60,63], and from South Asia, one study took place in Nepal [111], and the other in Bangladesh [110]. Three studies provided supplementation from enrollment till the end of pregnancy [60,92,111], whereas, two studies provided supplements from enrollment until 6 weeks postpartum [79] and 12 weeks postpartum [110], respectively, and one study did not indicate the intervention endpoint [86].

**Figure 2:**
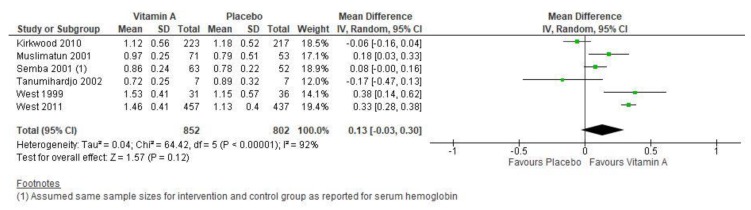
Forest plot for comparison vitamin A supplementation versus placebo/no vitamin A baseline to post-intervention for maternal serum/plasma retinol concentration (umol/L).

Vitamin A supplementation, compared to placebo, showed no impact on maternal mortality (average risk ratio (RR) 0.90, 95% Confidence Interval (CI) 0.68 to 1.18; studies = 3; GRADE: low-quality evidence), nor any effect on the risk of stillbirths (average RR 1.01, 95% CI 0.96 to 1.07; studies = 3) or maternal hemoglobin concentration (average MD 0.51 g/L, 95% CI −2.42 to 3.43; studies = 5). Supplementation may have improved maternal serum retinol concentration (average MD 0.13 umol/L, 95% CI -0.03 to 0.30; studies = 6) (Figure 2).

No subgroup analysis for primary outcomes was possible for this comparison due to an insufficient number of studies per subgroup of interest.

### 3.2. Zinc Supplementation Versus Placebo

Thirteen studies evaluated zinc supplementation compared to placebo in women during pregnancy [53,56,66,74,91,94,95,98,99,103,114,115,117]. Prawirohartono et al. was not included in meta-analysis because it reported no poolable outcomes, but was narratively synthesized [53] (see Supplementary Materials, Table S3). Of these, twelve were included in the meta-analyses [56,66,74,91,94,95,98,99,103,114,115,117]. Four studies were conducted in South Asia, with two of these conducted in Bangladesh [95,103], one in Pakistan [99], and one in Nepal [98]. Three studies were conducted in Latin America and the Caribbean, specifically two in Peru [115,117] and on in Chile [114]. From the Middle East and North Africa region, two studies were conducted in Iran [66,74], and two studies were conducted in sub-Saharan Africa, one study each in Ghana [91] and in Tanzania [94]. One study was conducted in Indonesia, in East Asia and the Pacific [56].

The majority of the studies (*n* = 8) provided zinc supplementation until the end of pregnancy [56,66,74,91,94,98,99,103], while two studies provided zinc until one month postpartum [115,117], and one study until 6 months postpartum [95]. One study did not explicitly state the endpoint of the intervention [114].

Zinc supplementation, compared to placebo, showed no impact on the risk of having a low birthweight baby (average RR 1.08, 95% CI 0.94 to 1.25; studies = 10; GRADE: moderate-quality evidence). A subgroup analysis was conducted for the outcome of low birthweight based on geographical region; no significant differences were observed (P for subgroup differences = 0.39). Further, zinc supplementation showed little to no effect on reducing the risk of pre-eclampsia/eclampsia (average RR 1.01, 95% CI 0.53 to 1.93; studies = 3), preterm birth (average RR 0.97, 95% CI 0.80 to 1.17; studies = 11) and infants considered SGA (average RR 1.05, 9% CI 0.97 to 1.13; studies = 3). In post-hoc analysis, no differences were observed between studies that provided zinc with additional micronutrients and studies that strictly provided zinc.

**Figure 3:**
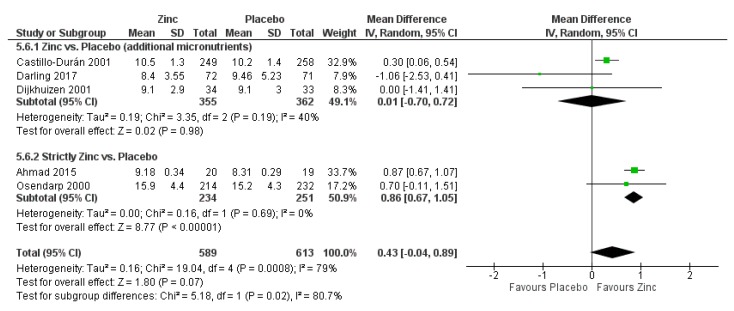
Forest plot for comparison zinc supplementation versus placebo/no zinc from baseline to post-intervention for maternal serum/plasma zinc concentration (umol/L).

However, zinc supplementation may have improved maternal serum/plasma zinc concentrations, although the lower limit of the confidence interval just crossed the line of no effect (average MD 0.43 umol/L, 95% CI -0.04 to 0.89; studies = 5) (Figure 3). In a post-hoc analysis, studies that provided strictly zinc showed a greater improvement in maternal serum/plasma zinc concentration (average MD 0.86 umol/L, 95% CI 0.67 to 1.05; studies = 2) than studies that provided additional micronutrients (average MD 0.01 umol/L, 95% CI -0.70 to 0.72; studies = 3).

### 3.3. Iron Supplementation Versus Placebo

Thirteen studies examined the effects of iron supplementation during pregnancy on maternal and infant health, compared to placebo [52,59,63,64,65,70,72,76,77,81,87,89,98]. In the meta-analyses, twelve studies were included [59,63,64,65,70,72,76,77,81,87,89,98]. Korkmaz et al. was not included because it did not report any outcomes of interest for meta-analysis; however, it was narratively synthesized [52] (see Supplementary Materials, Table S3).

Four studies were conducted in the East Asia and Pacific region—of which, three were conducted in China [59,64,65], and one in Indonesia [63]. Four studies were conducted in the Middle East and North Africa, specifically in Iran [70,72,76,77], and three studies were conducted in sub-Saharan Africa in Tanzania [81], the Gambia [87], and Niger [89]. One study was conducted in Nepal in the South Asia region [98]. The majority of studies provided supplements that contained 60 mg of elemental iron [63,64,65,70,81,87], while two studies gave 50 mg of iron [76,77], and three studies gave supplements with 30 mg of iron [59,72,98]. One study gave much higher amounts of elemental iron to women during their pregnancy; 100 mg [89]. Of the twelve studies, five provided folic acid supplementation as a co-intervention [59,76,81,87,98]. In terms of duration of intervention, the majority of studies (studies = 10) provided iron supplements until the end of pregnancy [59,64,65,70,72,76,81,87,89,98], while two studies gave supplementation until 6 weeks [77] and 8 weeks [63] postpartum, respectively.

**Figure 4:**
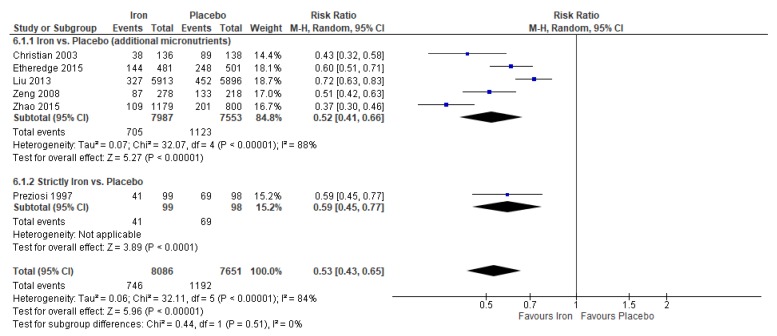
Forest plot for comparison iron supplementation versus placebo/no iron from baseline to post-intervention on the risk of maternal anemia.

**Figure 5:**
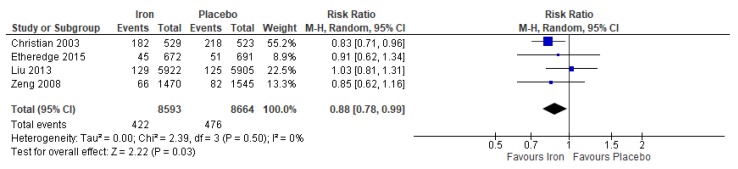
Forest plot for comparison iron supplementation versus placebo/no iron from baseline to post-intervention on the risk of low birthweight infants.

When compared to placebo or no treatment, iron supplementation reduced the risk of maternal anemia by 47% (average RR 0.53, 95% CI 0.43 to 0.65; studies = 6; GRADE: moderate-quality evidence) (Figure 4), and reduced the risk of having a low birthweight baby by 12% (average RR 0.88, 95% CI 0.78 to 0.99; studies = 4; GRADE: high-quality evidence) (Figure 5). However, findings showed no effect of iron on the risk of perinatal mortality (average RR 0.88, 95% CI 0.71 to 1.08; studies = 4; GRADE: high-quality evidence).

For maternal anemia, a post-hoc analysis was conducted between studies that gave strictly iron supplements versus placebo and studies that provided iron supplements with other additional micronutrients, typically vitamin A or folic acid; no significant differences between subgroups were observed (P for subgroup differences = 0.51) (Figure 4). Further, subgroup analyses were conducted for maternal anemia according to geographical region and the dose of iron in the intervention supplement. There were no significant differences between groups for both subgroup variables (geographical region: P for subgroup differences = 0.49 and dose of iron: P for subgroup differences = 0.69).

Among the secondary outcomes, iron supplementation improved maternal hemoglobin concentration (average MD 7.80 g/L, 95% CI 4.08 to 11.52; studies = 11) and maternal serum/plasma ferritin concentrations (average MD 25.30 ug/L, 95% CI 9.74 to 40.87; studies = 9). In a post-hoc analysis, no differences were observed for maternal hemoglobin concentration between studies that provided strictly iron and studies that gave additional micronutrients; however, there were significant differences for maternal serum/plasma ferritin concentrations. Studies that gave iron with additional micronutrients showed a greater effect on ferritin concentration (average MD 32.87 ug/L, 95% CI 15.39 to 50.34; studies = 6) than studies that gave only iron (average MD 7.09 ug/L, 95% CI 4.45 to 9.72; studies = 3). Further, iron supplementation reduced rates of iron deficiency when compared to placebo (average RR 0.54, 95% CI 0.40 to 0.74; studies = 4). This effect increased in studies that strictly gave iron (average RR 0.34, 95% CI 0.23 to 0.51; studies = 2) compared to studies that provided additional supplements with the iron (average RR 0.67, 95% CI 0.54 to 0.83; studies = 2).

Iron supplementation appeared to have little effect on maternal transferrin receptor concentration (average MD −0.16 mg/L, 95% CI −0.96 to 0.65; studies = 3), and on reducing the risk of pre-eclampsia/eclampsia (average RR 1.55, 95% CI 0.91 to 2.63; studies= 3), neonatal mortality (average RR 0.85, 95% CI 0.55 to 1.31), infant mortality (average RR 1.10, 95% CI 0.84 to 1.45; studies = 3), preterm births (average RR 0.94, 95% CI 0.63 to 1.41; studies = 6), and SGA infants (average RR 1.04, 95% CI 0.87 to 1.24; studies = 4).

### 3.4. Vitamin D Supplementation Versus Placebo

Eleven studies were included in the meta-analyses for vitamin D supplementation compared to placebo or no vitamin D [67,69,71,73,75,100,101,105,106,107,112]. More than half of the studies (studies = 6) were conducted in South Asia—of which, two were in Bangladesh [105,106], two in Pakistan [100,101], and two in India [107,112]. The other half of the studies (studies = 5) were conducted in the Middle East and North Africa region—all of which were in Iran [67,69,71,73,75]. Six studies provided vitamin D supplementation until the end of pregnancy [71,73,75,100,101,106], while three studies specifically stated that supplementation was provided for eight weeks [69], nine weeks [67], and 10 weeks [105] from the time of enrollment, respectively.

**Figure 6:**
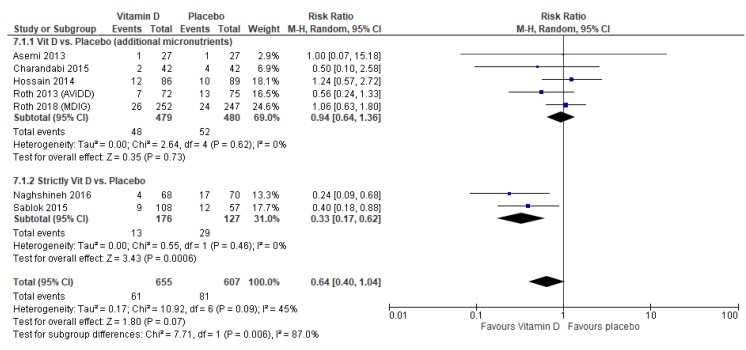
Forest plot for comparison vitamin D supplementation versus placebo/no vitamin D on the risk of preterm births.

Findings showed that vitamin D supplementation may have reduced the risk of preterm births by 36% (average RR 0.64, 95% CI 0.40 to 1.04; studies = 7), though the upper limit of the confidence interval just crossed the line of no effect (Figure 6). It was noted that studies that strictly gave vitamin D showed a greater reduction in preterm birth risk (average RR 0.33, 95% CI 0.17 to 0.62; studies = 2), compared to studies that provided additional supplements such as iron and folic acid (average RR 0.94, 95% CI 0.64 to 1.36; studies = 5) (Figure 6).

Vitamin D supplementation made no difference on the risk of infants born SGA (average RR 0.93, 95% CI 0.51 to 1.53; studies = 3), the risk of having a Caesarean section as a mode of delivery (average RR 1.05, 95% CI 0.94 to 1.18; studies = 5), or maternal serum/plasma calcium concentrations (average MD −0.06 mg/dL, 95% CI −0.21 to 0.09; studies = 5), but did significantly increase the vitamin D concentrations in pregnant mothers (average MD 44.70 umol/L, 95% CI 21.94 to 67.45; studies = 9).

No subgroup analysis was conducted for this comparison as no primary outcomes were reported for this comparison.

### 3.5. Calcium Supplementation Versus Placebo

Five studies assessed calcium supplementation in pregnant women [51,102,113,116,119]—of which, four were included in the meta-analysis [102,113,116,119]. Two of these studies were conducted in Latin America and the Caribbean [113,116], and one study took place in South Asia [102]. The fourth study was a multi-country study that was conducted in Peru, South Africa, Vietnam, and India [119]. Jarjou et al. was excluded on the basis that no common outcomes of interest were reported and could not be pooled with other studies [51]; however, the paper was narratively synthesized (see Supplementary Materials, Table S3).

Calcium supplementation, compared to placebo, did not impact the risk of having a low birthweight baby (average RR 0.99, 95% CI 0.95 to 1.04; studies = 3; GRADE: high-quality evidence), stillbirths (average RR 0.87, 95% CI 0.70 to 1.07; studies = 4), preterm births (average RR 0.84, 95% CI 0.65 to 1.08; studies = 4), or Caesarean section as a mode of delivery (average RR 0.99, 95% CI 0.84 to 1.15; studies = 3).

**Figure 7:**
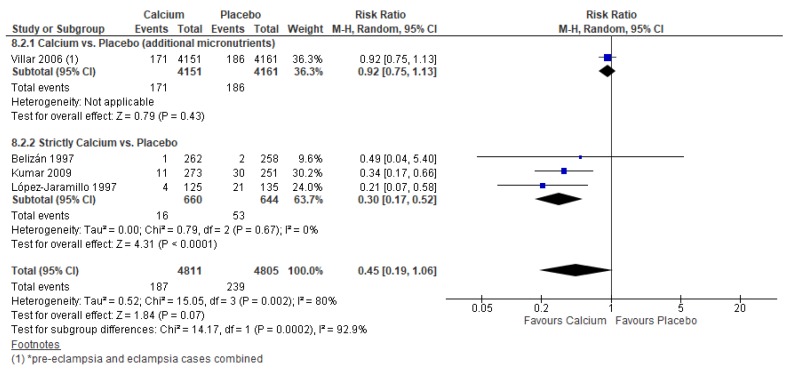
Forest plot for comparison calcium supplementation versus no calcium/placebo on the risk of pre-eclampsia/eclampsia in mothers during pregnancy.

However, calcium supplementation may have improved the risk of maternal pre-eclampsia/eclampsia, although the upper limit of the confidence interval just crossed the line of no effect (average RR 0.45, 95% CI 0.19 to 1.06; studies = 4) (Figure 7). In a post-hoc analysis, studies that provided only calcium to mothers showed a greater reduction in the risk of pre-eclampsia/eclampsia (average RR 0.30, 95% CI 0.17 to 0.52; studies = 3) compared to studies that provided additional micronutrients (average RR 0.92, 95% CI 0.75 to 1.13; studies = 1) (Figure 7).

No subgroup analysis for primary outcomes was conducted for this comparison due to an insufficient number of studies per subgroup of interest.

### 3.6. Iron and Folic Acid Versus Folic Acid Supplementation

Seven studies were included in the meta-analyses for iron and folic acid (IFA) supplementation versus folic acid (FA) supplementation or placebo [59,64,65,76,81,87,98]. Of these, three studies were conducted in the East Asia Pacific region, specifically in China [59,64,65], two studies were from sub-Saharan Africa [81,87], one from South Asia in Nepal [98], and one in Iran, the Middle East and North Africa region [76]. Across all studies, similar formulations of IFA and FA were utilized, with the majority of studies providing 50 or 60 mg of elemental iron and one study providing 30 mg of elemental iron [59]. Folic acid was typically provided in amounts of 400 µg, with two studies providing 5000 µg [81,87] and another 1000 µg of folic acid [76].

**Figure 8:**
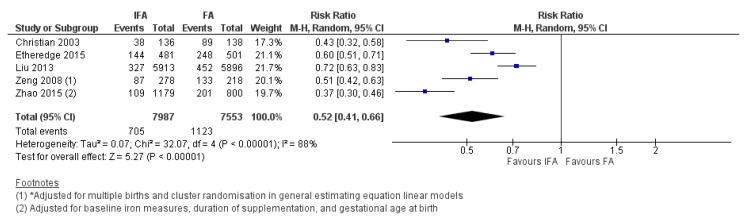
Forest plot of comparison Iron-Folic Acid (IFA) versus Folic Acid (FA) supplementation/placebo, from baseline to post-intervention on the risk of maternal anemia.

**Figure 9:**
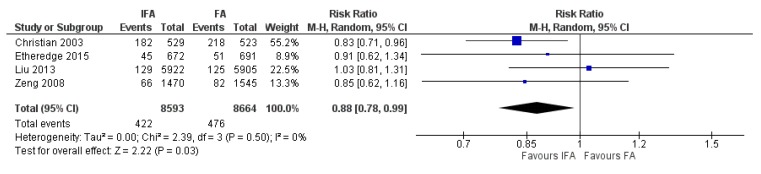
Forest plot of comparison IFA versus FA supplementation/placebo, from baseline to post-intervention on the risk of low birthweight infants.

IFA supplementation compared to FA, across five studies [59,64,65,81,98], showed a 48% reduction in the risk of maternal anemia in the third trimester of pregnancy (average RR 0.52, 95% CI 0.41 to 0.66; studies = 5; GRADE: moderate-quality evidence) (Figure 8). For the risk of low birthweight babies, a 12% reduction was noted across four studies (average RR 0.88, 95% CI 0.78 to 0.99; studies = 4; GRADE: high-quality evidence) (Figure 9). However, IFA did not reduce the risk of perinatal mortality (average RR 0.88, 95% CI 0.71 to 1.08; studies = 4; GRADE: moderate-quality evidence).

Notable secondary outcomes that showed significant increases with IFA supplementation were maternal hemoglobin concentration (average mean difference (MD) 6.95 g/L, 95% CI 2.80 to 11.1; studies = 7) and maternal serum/plasma ferritin concentration (average MD 15.87 ug/L, 95% CI 2.96 to 28.79; studies = 5). However, IFA supplementation did not show significant differences for the following outcomes: maternal serum/plasma transferrin receptor concentration (average MD −0.16 mg/L, 95% CI -0.96 to 0.65; studies = 3), neonatal mortality (average RR 0.85, 95% CI 0.55 to 1.31; studies = 3), preterm births (average RR 0.96, 95% CI 0.64 to 1.44; studies = 5), SGA infants (average RR 1.03, 95% CI 0.87 to 1.23; studies = 4), and infant mortality (average RR 1.10, 95% CI 0.84 to 1.45; studies = 3).

No subgroup analysis for primary outcomes was conducted for this comparison due to an insufficient number of studies per subgroup of interest.

### 3.7. Multiple Micronutrient Supplementation Versus Iron Folic Acid Supplementation

Thirty-four studies were identified and included in the meta-analyses for MMN supplementation compared to iron with or without folic acid supplementation [56,57,58,59,60,61,62,64,66,68,74,78,80,82,83,85,88,90,91,92,93,96,97,98,99,104,105,106,108,109,115,117,118,119]. Ten of these studies were conducted in the sub-Saharan Africa region—of which, two were conducted in Ghana [80,91], two in Malawi [78,92], and one each in Burkina Faso [90], Gambia [88], Guinea-Bissau [85], Tanzania [82], Zimbabwe [83], and Niger [93]. From the East Asia and Pacific region, four studies were conducted in Indonesia [56,60,61,62], two in Vietnam [57,58], and two in China [59,64]. Eight studies were from the South Asia region—of which, five were from Bangladesh [97,105,106,108,109], two were conducted in Nepal [98,104], and two in Pakistan [96,99]. Within Latin America and the Caribbean, two studies were conducted in Peru [115,117], and one in Mexico [118]. Three studies were conducted in Iran, in the Middle East and North Africa [66,68,74].

Of these, 11 studies used the UNIMMAP formulation, developed by UNICEF, WHO and the United Nations University (See Table 1) [58,59,61,62,64,85,90,93,96,104,108]. The UNIMMAP supplement contains 800 retinol equivalents of Vitamin A, 200 IU Vitamin D, 10 mg of Vitamin E, 70 mg of Vitamin C, 1.4 mg of thiamin, 1.4 mg of riboflavin, 18 mg of niacin, 1.9 mg of pyridoxine, 2.6 mg of cobalamin, 400 µg of folic acid, 30 mg of iron, 15 mg of zinc, 2 mg of copper, 65 mg of selenium and 150 µg of iodine. Three studies used an adapted UNIMMAP formulation whereby the same combination, but different dosages, of vitamins and minerals were included in the supplement [57,88,109]. The remaining 20 studies used non-UNIMMAP formulations for their MMN supplement, comprised of as few as three micronutrients and vitamins and some of which included other components such as pantothenic acid, vitamin K and manganese [56,60,66,68,74,78,80,82,83,91,92,97–99,105,106,115,117–119).

**Figure 10:**
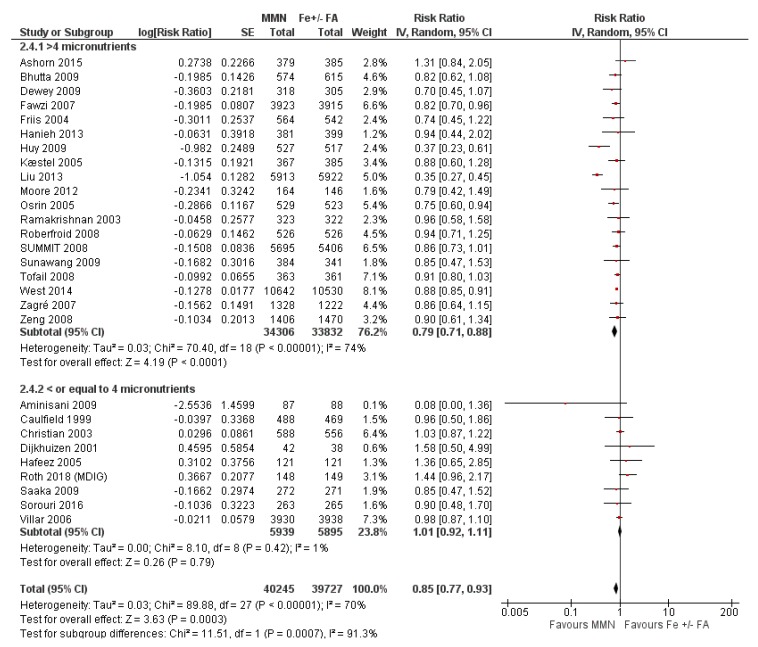
Forest plot of comparison MMN versus IFA supplementation, from baseline to post-intervention, on the risk of low birthweight infants.

Compared to iron supplementation with or without folic acid, MMN supplementation demonstrated no impact on maternal mortality (average RR 1.04, 95% CI 0.71 to 1.51; studies = 7; GRADE: moderate-quality evidence); perinatal mortality (average RR 1.00, 95% CI 0.90 to 1.11; studies = 16; GRADE: high-quality evidence); maternal anemia (average RR 1.02; 95% CI 0.95 to 1.10; studies = 16; GRADE: high-quality evidence); and iron deficiency anemia (average RR 1.12, 95% CI 0.62 to 2.02; studies = 4; GRADE: very low-quality evidence). However, MMN supplementation showed a 15% reduction in the risk of delivering a low birthweight baby (average RR 0.85, 95% CI 0.77 to 0.93; studies = 28 studies; GRADE: high-quality evidence) when compared to iron with or without folic acid (Figure 10). A post-hoc analysis revealed a greater reduction in the risk of LBW (average RR 0.79, 95% CI 0.71 to 0.88; studies = 19) in studies whose MMN formulation contained > 4 micronutrients, compared to studies whose MMN supplement contained only 3 or 4 components (average RR 1.01, 95% CI 0.90 to 1.11; studies = 9) (Figure 10). For a description of the subgroup analyses for these primary outcomes, see Section 3.7.2 Subgroup Analyses for MMN vs. IFA supplementation.

**Figure 11:**
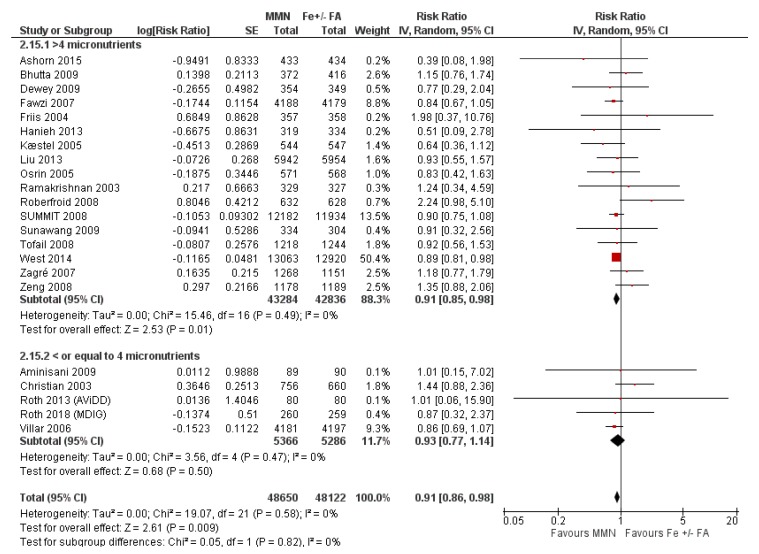
Forest plot of comparison MMN versus IFA supplementation, from baseline to post-intervention, on the risk of stillbirths.

**Figure 12:**
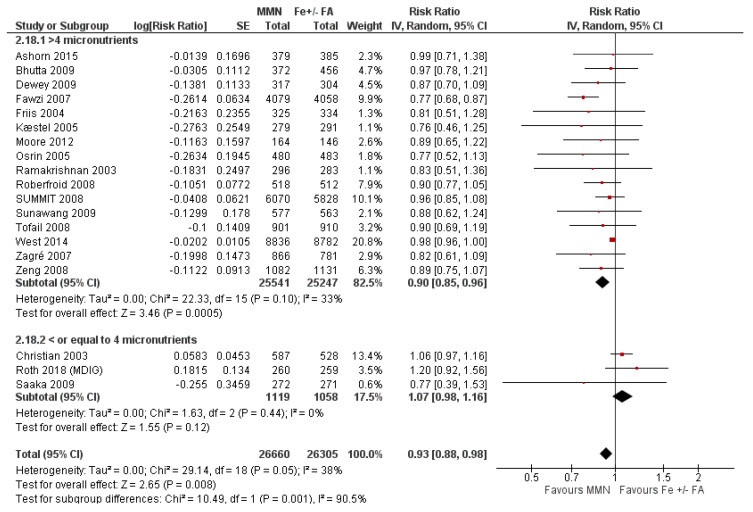
Forest plot of comparison MMN versus IFA supplementation, from baseline to post-intervention, on the risk of small-for-gestational-age (SGA) infants.

MMN supplementation showed a 9% reduction in the risk of stillbirths (average RR 0.91, 95% CI 0.86 to 0.98; studies = 22) (Figure 11.) and a 7% reduction in the risk of SGA infants (average RR 0.93, 95% CI 0.88 to 0.98; studies = 19) (Figure 12). For SGA infants, a post-hoc analysis showed a greater reductive effect among studies that used MMN supplements with > 4 micronutrients (average RR 0.90, 95% CI 0.85 to 0.96; studies = 16) compared to studies whose supplements had 3 or 4 components (average RR 1.07, 95% CI 0.98 to 1.16; studies = 3) (Figure 12).

MMN supplementation probably improved the reduction in preterm births although the upper limit of the CI just crossed the line of no effect (average RR 0.96, 95% CI 0.91 to 1.01; studies = 29). Interestingly, in a post-hoc analysis, studies that provided MMN containing only 3 or 4 micronutrients showed a slightly greater reduction in the risk of preterm births (average RR 0.93, 95% CI 0.84 to 1.02; studies = 11) compared to studies that gave MMN with >4 micronutrients (average RR 0.97, 95% CI 0.91 to 1.03; studies = 18), although for both subgroups, the upper limits of their confidence intervals just crossed the line of no effect.

#### 3.7.1. Child Health and Developmental Outcomes

**Figure 13:**
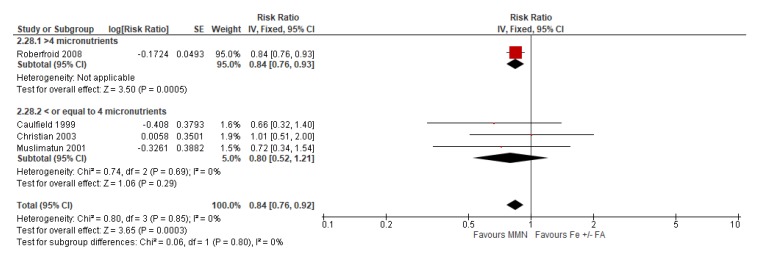
Forest plot of comparison MMN versus IFA supplementation, from baseline to post-intervention, on the risk of diarrhea in children.

In terms of child health and development outcomes, antenatal MMN supplementation showed a 16% reduction in the risk of diarrhea among children ages 6 months to under-five, when compared to IFA (average RR 0.84; 95% CI 0.76 to 0.92; studies = 4) (Figure 13). Further, MMN supplementation showed a slight improvement in child serum/plasma retinol concentration (average MD 0.06 umol/L, 95% CI 0.02 to 0.09; studies = 3). However, MMN supplementation showed no effect on hemoglobin concentration, zinc serum/plasma concentration or anemia in children. In terms of nutritional status indicators, MMN supplementation did not improve the risk of child wasting (average RR 1.02, 95% CI 0.88 to 1.18; studies = 5), stunting (average RR 0.99, 95% CI 0.92 to 1.07; studies = 7) and child underweight status (average RR 0.95, 95% CI 0.84 to 1.07; studies = 4).

MMN supplementation, compared to IFA, was the only comparison in this review to include studies that reported child development outcomes. Four child development scores were meta-analyzed: executive function, general intelligence, verbal comprehension and language, and motor function. These scores were conducted in children aged 6 months to early adolescence (10–14 years). Of the four development outcomes, MMN supplementation seemed to improve only executive function (standard mean difference (MD) 0.09, 95% CI 0.01 to 0.17; studies = 3) but showed little to no effect on the other scores: general intelligence (standard mean difference (MD) 0.00, 95% CI −0.06 to 0.07; studies = 8), verbal comprehension and language (standard MD 0.02, 95% CI −0.13 to 0.16; studies = 4), and motor function (standard MD −0.02, 95% CI −0.17 to 0.13; studies = 7).

#### 3.7.2. Subgroup Analyses for MMN vs. IFA Supplementation

Subgroup analyses were conducted for studies comparing MMN to IFA supplementation for maternal mortality, maternal anemia and perinatal mortality, in terms of MMN formulation (UNIMMAP vs. adapted UNIMMAP vs. non-UNIMMAP formulation), geographical region, duration of intervention and dose of iron (mg). No significant differences were identified between groups for all of the subgroup variables.

**Figure 14:**
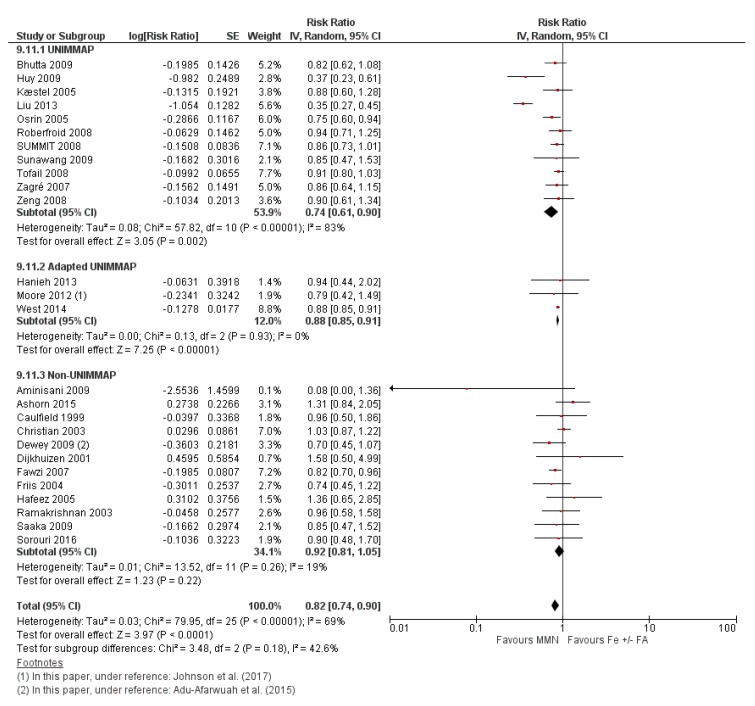
Forest plot of MMN versus IFA supplementation, a subgroup analysis of the risk of low birthweight infants by multiple micronutrient formulation (UNIMMAP versus adapted-UNIMMAP versus non-UNNIMAP).

The outcome of low birthweight showed significant differences between groups based on MMN formulation. In the subgroup of women who took the UNIMMAP formulation (average RR 0.74, 95% CI 0.61 to 0.90; studies = 11), compared to participants that used an adapted formulation (average RR 0.88, 95% CI 0.85 to 0.91; studies = 3) or non-UNIMMAP formulation (average RR 0.92, 95% CI 0.81 to 1.05; studies = 12), there was a greater reduction in the risk of having a low birthweight baby (P for subgroup differences = 0.18) (Figure 14).

As well, a greater reduction in the risk of low birthweight babies was evident amongst participants of studies conducted in the Western Pacific region when compared to studies conducted in other geographical regions (average RR 0.46, 95% CI 0.38 to 0.56; P for subgroup differences <0.00001). When considering the dose of iron provided in the intervention, studies that utilized an iron dose <60 mg showed a greater reduction in the risk of low birthweight (average RR 0.79, 95% CI 0.69 to 0.89; studies = 18) compared to those that used 60 mg of elemental iron in their MMN supplements (average RR 0.96, 95% CI 0.83 to 1.12; P for subgroup differences = 0.05).

### 3.8. Lipid-Based Nutrient Supplementation (LNS) Versus MMN Supplementation

Four RCTs assessed LNS supplementation, compared to MMN supplementation in pregnant women [78,80,84,88]. All studies were conducted in sub-Saharan Africa, specifically Malawi [78], Ghana [80], Burkina Faso [84], and Gambia [88]. In two studies, women were provided LNS supplementation until the end of pregnancy [84,88], whereas, in the other two studies, mothers were given supplements for up to 6 months postpartum [78,80].

**Figure 15:**
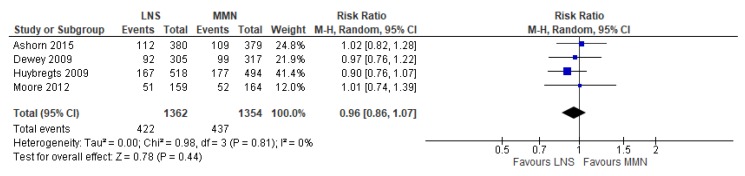
Forest plot for comparison LNS supplementation versus MMN from baseline to post-intervention for the risk of small-for-gestational age infants.

Of the primary outcomes, LNS supplementation showed no effect on the risk of having a low birthweight baby (average RR 0.92, 95% CI 0.75 to 1.13; studies = 4; GRADE: moderate-quality evidence) and did not impact perinatal mortality (average RR 1.01, 95% CI 0.65 to 1.65; studies = 3; GRADE: low-quality evidence). Among the secondary outcomes examined, LNS supplementation showed no effect on the risk of miscarriage (average RR 1.12, 95% CI 0.69 to 1.80; studies = 3), neonatal mortality (average RR 0.81, 95% CI 0.45 to 1.45; studies = 3) and preterm birth (average RR 1.15, 95% CI 0.93 to 1.42; studies = 4). LNS also did not show an effect on the risk of SGA infants, although the upper limits of the CI just crossed one (average RR 0.96; 95% CI 0.86 to 1.07) (Figure 15).

### 3.9. Sensitivity Analysis

Studies that were assessed with a high risk of bias in at least one domain or studies that were assessed as unclear risk in two or more domains were removed from the meta-analyses in a sensitivity analysis based on the risk of bias assessments. With the removal of one study, there was a 75% reduction in the risk of stillbirths (average RR 0.25, 95% CI 0.08 to 0.78; studies = 2). This is compared to the original effect estimate which showed no effect of LNS, compared to MMN, on stillbirths (average RR 0.47, 95% CI 0.12 to 1.81; studies = 3). For the rest of the comparisons, there were no significant changes to any of the outcomes.

A post-hoc sensitivity analysis was conducted to evaluate if any outcomes were affected by studies that declared a conflict of interest. In this review, five studies reported a conflict of interest (64, 78, 80, 104, 109); exclusion of these studies did not significantly affect any outcome.

## 4. Discussion

The purpose of this review was to evaluate multiple forms of micronutrient and vitamin supplementation during pregnancy on a multitude of outcomes, including maternal, fetal, and child outcomes. These included outcomes of morbidity, mortality, nutritional and biochemical status in both mother and child, and longitudinal child health and developmental outcomes, such as general intelligence index scores. This is the first systematic review that has attempted to compile evidence from efficacy and effectiveness studies evaluating the effects of micronutrient and vitamin supplementation on such a wide breadth of outcomes, conducted from 1995 onwards across all LMICs, in a singular report. With that, it must be noted that certain comparisons were not included in meta-analyses, although narratively synthesized, due to an insufficient number of eligible studies for meta-analysis. These separated comparisons include iodine, folic acid alone, vitamin B12 and vitamin D plus calcium compared to placebo or no micronutrient or vitamin. Still, the overall findings of this review suggest that supplementation improves key maternal, fetal, and child outcomes, and support the use of some of these strategies during pregnancy in low- and middle-income settings.

Notable improvements were observed with MMN supplementation, compared to IFA, for outcomes, such as stillbirths, SGA, and low birthweight babies. These improvements are corroborated by findings of other systematic reviews evaluating the use of MMN supplementation [32,33,34,35,36,37,42]. In terms of child health outcomes, MMN supplementation showed improvement in child serum/plasma retinol concentration and in the reduction of diarrhea incidence amongst children, which are new findings not previously reported elsewhere. MMN versus IFA supplementation was the only comparison in this review that, provided the number of studies, could meta-analyze child development outcomes, including general intelligence, verbal comprehension and language, motor function and executive function. The only development outcome that showed improvement with MMN supplementation was executive function, although far fewer studies contributed to this analysis compared to the other development outcomes. There was significant diversity in the tests and scores used to measure development, age of participants and time of assessment, lending to difficulty in pooling results effectively.

Post-hoc and subgroup analyses indicated a greater effect of MMN supplementation on low birthweight in studies that utilized a supplement that contained more than four micronutrient components compared to studies that utilized a supplement with three components, and UNIMMAP formulation compared to adapted UNIMMAP and non-UNIMMAP formulations, respectively. Reviews by Ramakrishnan et al. (2012) and Bhutta et al. (2012) compared supplementation of pregnant women with five or more micronutrients to supplementation with three or fewer micronutrients, and both reviews noted a stronger reduction in the risks of low birthweight and SGA infants in the former group [32,37]. These findings should underscore the consideration of MMN as the preferred standard of care (versus IFA) to address certain important maternal and infant outcomes.

Across all comparisons, micronutrient and vitamin supplementation had little to no effect on mortality outcomes (maternal, neonatal, perinatal, and infant mortality), which is consistent with other systematic reviews [19,31,32,34,35,37,38,39,40,41]. However, these findings differ from those reported in the meta-analysis of six RCTs upon which the WHO based their 2016 antenatal care guidelines [23]. In these guidelines, the WHO did not universally recommend the use of MMN supplementation due to an observed increased risk of neonatal mortality in the meta-analysis. A recent review by Sudfeld et al. re-ran the WHO meta-analysis (studies = 6) with the inclusion of additional and newer studies [42], and noted no increased risk of neonatal mortality with MMN supplementation (studies = 17). Similarly, an updated Cochrane review on MMN supplementation in pregnancy reported no negative impact of this intervention on neonatal mortality[35]. These findings further contribute to the increasing body of high-quality evidence in support of MMN supplementation [42].

For other comparisons, improvements were noted in only a few outcomes, indicating that micronutrient-specific supplementation could be used to address specific outcomes where they are highly prevalent in a population. IFA and iron supplementation both improved maternal anemia and low birthweight, which are similar findings to those reported in another recent review [31]. LNS supplementation, compared to MMN, demonstrated a possible slight reduction in the risk of SGA infants, but had no effect on preterm birth, stillbirths, and low birthweight infants; which are corroborated by findings in a systematic review by Das et al. [38]. However, it is important to note in this review, this intervention included only four studies, suggesting the need for further research comparing LNS to MMN supplementation and its effects on maternal and infant health. Further, the Cochrane review did report observed differences for low birthweight and SGA infants when comparing LNS to IFA supplementation [38], which was a comparison not included in this review. Although it is recognized that IFA is typically the standard of care, and/or may be given as a co-intervention when comparing LNS to placebo or no treatment, it was noted that eligible studies for this comparison reported dissimilar amounts of iron and folic acid between the LNS and IFA supplements. For the reason that this would not be a true comparison between LNS and placebo or no treatment, these studies were not meta-analyzed.

Other similarities and differences were observed between this review and other systematic reviews for certain outcomes following maternal supplementation with zinc, vitamin A, vitamin D, and calcium [19,39,41,120]. Compared to a recent review by Ota et al. (2015) which showed reduction in low birthweight babies and preterm births with zinc supplementation, this review showed minimal to no effect on the same outcomes [19]. However, the lack of any effect of zinc supplementation on the reduction of SGA infants was comparable across both reviews. Similar to a recent Cochrane review by McCauley et al. (2015) on vitamin A supplementation, very few studies were found to assess vitamin A compared to placebo [40]. This review observed no improvements in maternal mortality or in stillbirths with vitamin A supplementation in pregnant mothers, which is corroborated by the recent Cochrane review [40].

In terms of vitamin D supplementation, a possible reduction in the risk of preterm birth was observed, while other recent reviews showed little to minimal effect [39,120]. Further, Roth et al. (2017) showed a significant reduction in the risk of SGA infants, while this review showed no significant effect; the recent Cochrane review update by Palacios et al. (2019) did not evaluate this outcome [39,120]. Lastly, similarities were noted between this review and a recent Cochrane review by Hofmeyr et al. (2018) which examined calcium supplementation and possibly improved rates of pre-eclampsia/eclampsia among mothers [41]. This review indicated little to no improvement in low birthweight as a result of calcium supplementation compared to placebo; however, a recent systematic review found a 15% reduction in the risk of low birthweight infants [41].

The discrepancies described above point to differences in review design, wherein many other reviews included studies from high-income settings and also included participants that were unhealthy, had known micronutrient deficiencies, or a previous history of gestational hypertension; altogether, these overt differences in study populations are likely to modify the effects of the supplementation interventions examined [19,39,41,120]. These differences also highlight a limitation of this review in the exclusion of certain populations, such as pregnant women with anemia, micronutrient deficiencies, high risk pregnancy disorders or chronic illness. It is acknowledged that these conditions are highly prevalent in maternal populations living in LMICs. Moving forward, an important consideration for future research is to disaggregate results by maternal nutritional status at baseline. A recent review by Smith et al. reported a larger effect of MMN supplementation on birth outcomes in women with anemia, compared to non-anemic women [43]. Given the review’s eligibility criteria, there were insufficient studies to disaggregate data by such factors, and specific groups that may benefit most from certain forms of supplementation could not be identified.

Similarly, the ability to disaggregate results by other variables, such as maternal age, sex of infant, duration of intervention and dose of intervention was limited in this review given the lack of data availability. As such, this review was limited in conducting subgroup analyses for a few comparisons (zinc vs. placebo, iron vs. placebo and MMN vs. IFA), and for only a few primary outcomes. Importantly, this review cannot comment on recommended optimal doses of micronutrient supplementation and duration of supplementation. The majority of studies in this review recruited already pregnant women whose supplementation programme began within the first or second trimester; however, no study specifically recruited and supplemented women in the preconception period. This is a notable limitation given existing evidence supporting the importance of preconception health in mothers to reduce maternal, infant and child morbidity and mortality [121,122,123]. It is possible that the lack of effect of micronutrient and vitamin supplementation on mortality outcomes and remaining uncertainty surrounding the effects of supplementation on preterm births, miscarriage and congenital anomalies may be a result of supplementation implemented too late. This would have great implication in low- and middle-income settings where many women have poor nutritional status and health prior to conception.

Post-hoc exploratory analyses for both primary and secondary outcomes were conducted, and compared studies that provided strictly the micronutrient in question to studies that provided additional micronutrients, typically iron-folic acid and vitamin A. The aim of these analyses was to disaggregate possible differences in the true effects of the micronutrient itself from possible modifier effects from other micronutrients. Post-hoc analyses were conducted for zinc, iron, vitamin D and calcium supplementations compared to placebo, and did demonstrate differences in the true effects of the micronutrient itself. Maternal zinc serum/plasma concentration, maternal iron deficiency and pre-eclampsia/eclampsia showed greater improvements in studies that provided strictly zinc, iron and calcium respectively, compared to studies that provided additional micronutrients. For MMN vs. IFA, post-hoc analyses compared studies who provided MMN supplements containing >4 micronutrients to studies whose supplements contained only 3 or 4 components. Generally, the supplementation with >4 micronutrients performed better in terms of improving maternal and child health outcomes, such as low birthweight, stillbirths and SGA.

Another limitation of this review is the inability to examine data and comment on the pregnant adolescent population. Globally, 16 million girls ages 16 to 19 years, and 2.5 million girls under age 16, give birth every year, mainly in LMICs [124]. Special attention must be given to this demographic, as pregnant adolescents undergo significant physical and physiological changes due to puberty, which is further compounded by the nutritional demands and requirements of pregnancy. Unfortunately, many of these adolescent girls begin their pregnancy already malnourished, micronutrient-deficient and/or stunted. Poor health status at the start of pregnancy sets up both mother and future child to face poor delivery, birth and health outcomes. As such, finding a strategy to prevent or mitigate these deficiencies in adolescents is of utmost importance. Unfortunately, none of the included studies disaggregated data by age group; although they included adolescent mothers. This will be an important consideration for future updates and de novo research.

Similarly, birth outcomes were typically not disaggregated by sex, which limited this review from conducting subgroup analyses by sex of the infant. This is also an important consideration for future research, as previous evidence suggests differences between female and male infants for certain mortality and morbidity outcomes [33,109,125,126]. Further, a recent review by Smith et al., authors noted a 15% greater reduction in mortality in female infants compared to their male counterparts [43]. Studies postulate that these observed differences are a result of differences in birth size; with males being typically larger in the womb [109,127]. It is recommended that future updates and research consider sex disaggregation of key fetal and infant outcomes, including preterm birth, low birthweight, neonatal and infant mortality, stillbirths and SGA. It is important to note, however, that sex differences in infant outcomes should not be used as evidence to support or encourage selective supplementation of mothers during pregnancy. Rather, evidence regarding sex differences should be used to better support infant and child care and consider how these differences at infancy might affect long-term growth and development in the child.

Despite the numerous antenatal and maternal health programmes in LMICs, and the substantial number of studies included in this review, only two included studies were effectiveness trials [58,83] that met our criteria related to study design (RCT or quasi-experimental design). Recent evidence evaluating health and nutrition programmes to reduce stunting in LMICs noted that all programme evaluations were cross-sectional in design, with no true control and intervention areas or groups, limiting the causality of findings [128]. In this systematic review by Hossain et al. (2017) the importance of the use of both nutrition-specific interventions (e.g., micronutrient supplementation) and nutrition-sensitive interventions (e.g., maternal mental health and social safety nets) to reduce stunting was evident, inciting support for more robust designs for effectiveness studies and programmes [128]. As evaluation of micronutrient and vitamin supplementation continues, these types of studies and rigorous evaluations should be considered and included in future review updates.

## 5. Conclusions

Micronutrient and vitamin supplementation is a key intervention to promote maternal and child nutrition, health and well-being, and implementation should be continued, especially during pregnancy amongst mothers living in LMICs regardless of maternal nutritional status. Specifically, this comprehensive review indicates that MMN supplementation should be considered as the preferred option for standard prenatal care, compared to IFA, especially for outcomes such as stillbirths, SGA and low birthweight infants. Single micronutrient and vitamin supplementation also show improvements for specific outcomes, such as calcium on the risk of pre-eclampsia/eclampsia, and vitamin A on serum/plasma retinol concentration in mothers. LNS supplementation, compared to MMN, involved few studies in this review, highlighting the need for further research to better understand differences between the two types of supplementation.

Overall, the benefits of micronutrient and vitamin supplementation during pregnancy have been well corroborated by this review, along with many other systematic reviews. Given this, future research should consider conducting more detailed analyses to identify optimal dosages, supplement formulations, and duration of implementation of different micronutrient and vitamin supplements. These may also be tailored to specific groups of mothers, based on maternal nutritional status at baseline. Specific attention and consideration must be made to the pregnant adolescent population given their unique health and development needs, especially those living in low- and middle-income countries. While few studies reported longitudinal health outcomes, future research may also want to evaluate the effects of maternal micronutrient and vitamin supplementation during pregnancy on early childhood development and long-term health in under-five and school-aged children. Future research and implementation in these areas, while continuing our efforts in maternal, fetal and infant health, will not only support better child and adolescent health, but also future maternal health as these children and adolescents grow into the next generation of mothers.

## Figures and Tables

**Table 1:** Daily Recommended Dietary Allowance for Vitamins, Micronutrients and Macronutrients for Pregnancy.

Vitamins, Micronutrients and Macronutrients	Unit	Institute of Medicine Recommended Dietary Allowance (RDA) for Pregnancy		
		14–18 Years	19–30 Years	31–50 Years
Vitamin A (retinol)	µg	750	770	770
Vitamin B1 (thiamin)	mg	1.4	1.4	1.4
Vitamin B2 (riboflavin)	mg	1.4	1.4	1.4
Vitamin B3 (niacin)	mg	18	18	18
Vitamin B5 (pantothenic acid)	mg	*6*	*6*	*6*
Vitamin B6 (pyridoxine)	mg	1.9	1.9	1.9
Vitamin B7 (biotin)	µg	*30*	*30*	*30*
Vitamin B9 (folate)	µg	600	600	600
Vitamin B12 (cobalamine)	µg	2.6	2.6	2.6
Vitamin C (ascorbate)	mg	80	85	85
Vitamin D (cholecalciferol)	IU	15	15	15
Vitamin E (tocopherol acetate)	mg	15	15	15
Vitamin K (phytomenadione)	µg	*75*	*90*	*90*
Choline	mg	*450*	*450*	*450*
Calcium	mg	1300	1000	1000
Chromium	g	*29*	*30*	*30*
Copper	µg	1000	1000	1000
Fluoride	mg	*3*	*3*	*3*
Iodine	µg	220	220	220
Iron	mg	27	27	27
Magnesium	mg	400	350	360
Phosphorus	mg	1250	700	700
Selenium	µg	60	60	60
Zinc	mg	12	11	11
Potassium	mg	*2600*	*2900*	*2900*
Sodium	mg	*1500*	*1500*	*1500*
Chloride	g	*2.3*	*2.3*	*2.3*
Carbohydrate	g	175	175	175
Fat	g	Not determined	Not determined	Not determined
Linoleic Acid	g	*13*	*13*	*13*
α-Linoleic Acid	g	*1.4*	*1.4*	*1.4*
Protein	g	71	71	71

*Note: Adequate intakes are in *italics*.

**Table 2:** Inclusion and Exclusion Criteria.

**Inclusion Criteria**
Low- or middle-income country (LMIC) setting
Pregnant mothers of any age and parity, who are healthy. Though the aim is to include healthy pregnant woman, the likelihood of the women having one or more micronutrient deficiencies at baseline is common; however, women will not be excluded on this basis
Types of interventions ○ Single micronutrient supplementation (calcium, vitamin D, iodine, folic acid, iron, vitamin A, zinc, vitamin B12) compared to placebo ○ Iron folic acid (IFA) supplementation compared to folic acid (FA) alone or placebo ○ Vitamin D plus calcium supplementation compared to placebo ○ Multiple micronutrient (MMN) supplementation compared to IFA supplementation or placebo ○ Lipid-based nutrient (LNS) supplementation compared to MMN supplementation
Relevant study designs ○ Randomized controlled trials (RCTs) with a control arm ○ Quasi-experimental designs including: ▪ Natural experiments ▪ Controlled before/after studies ▪ Regression discontinuity designs ▪ Interrupted time series designs
Date collection in 1995 or later
**Exclusion Criteria**
Unhealthy population (e.g., populations with chronic or genetic diseases, such as human immunodeficiency virus (HIV), tuberculosis (TB) or metabolic disorders)
Irrelevant study designs: reviews and observational study designs (i.e., cross-sectional studies)

## Supplementary Materials

The following are available online at https://www.mdpi.com/2072-6643/12/2/491/s1, Table S1: Search Strategy; Table S2: Summary of Study Characteristics, Table S3: Descriptive Summary of Additional Studies (not included in meta-analyses), Figure S1: Additional Forest Plots.
