# Supplementary material for: Vitamin and Mineral Supplementation During Pregnancy on Maternal, Birth, Child Health and Development Outcomes in Low- and Middle-Income Countries: A Systematic Review and Meta-Analysis
Source: Nutrients. 2020 Feb 14;12(2):491. doi: 10.3390/nu12020491 (PMC7071347; doi:10.3390/nu12020491)
Supplement: Supplementary file 1 [file nutrients-12-00491-s001.pdf]

Supplementary Materials

Table S1. Search Strategy

Indicator	Searches
1	**Population:**<br>Pregnant women/ or Mothers/ or Maternal Age/ or Pregnancy in Adolescence/ or Pregnancy, Multiple/ or exp Perinatology/ or First Trimester, Pregnancy/ or Second Trimester, Pregnancy/ or Third Trimester, Pregnancy/ or Obstetrics/ or exp Maternal Behavior/ or Surrogate Mothers/ or Pregnancy Outcome/ or Prenatal Care/ or Perinatal Care/ or Postnatal Care/ or Postpartum Period/ or Lactation/ or Pregnancy Complications/ or (pregnan* or pregnan* wom#n* or mother* or mom* or surroga* or matern* or preconcept* or pre-concept* or "pre concept*" or periconcept* or peri-concept* or "peri concept* " or "multi*pregnan*" or "child birth " or child-birth or childbirth or "term birth or trimester" or (pregnan* and (expecting or reproductive age or women of reproductive age or WRA)) or partus or lactation or fetal or foetal or fetus or foetus or obstetric* or labo#r or childbear* or "child-bear*" or "gestation*" or (("reproductive age" or "wom#n of reproductive age") and pregnan*) or antenatal or ante-natal or "ante natal " or pre-natal or "pre natal " or prenatal or perinatal or peri-natal or "peri natal" or prepartum or pre-partum or "pre partum" or perinatology or peripartum or peri-partum or "peri partum " or puerper* or postpartum or post-partum or "post partum " or postnatal or post-natal or "post natal " or surrogate or "pregnant wom#n" or paturiency or child-carrying or "child carrying").mp. or ((pregnan* wom#n* or matern* or pregnan*) and ("postnatal care" or "post-partum")).tw,kf.
2	**Intervention 1: Single and Multiple Micronutrient**<br><br>Micronutrients/ or vitamins/ or minerals/ or exp iron/ or exp iron compounds/ or iron, dietary/ or vitamin A/ or exp iodine/ or exp zinc or exp zinc compounds/ or exp vitamin D/ or (micronutrient* or multinutrient* or multi-nutrient* or "multi*nutrient" or "multimicro-nutrient*" or "multimicronutrient*" or multivitamin* or "multi-vitamin*" or multimineral* or "multi-mineral*" or "multiple micro nutrient*" or "multiple micronutrient" or micro-nutrient* or MMN or "essential vitamins*" or minerals* or "m.v.i. pediatric" or "trace element*" or "trace mineral*" or "trace metal" or vitamin* or davitamin or "vitamin d" or "hydroxyvitamin d" or vitamin-d or "25 hydroxyvitamin d" or "25 hydroxy-vitamin d" or "25-hydroxyvitamin d" or "25-hydroxy-vitamin d" or "25-hydroxyvitamin d" or 25ohd or "25-oh-vitamin d" or 25-ohd or "vitamin d2" or vitamin-d2 or "25-hydroxyvitamin d2" or "25-hydroxy-vitamin d2" or "vitamin d3" or vitamin-d3 or "25 hydroxyvitamin d3" or "25 hydroxyvitamin d3" or "25-hydroxy-vitamin d3" or calcidiol or calcifediol or calcium or retinol* or retinal* or Retinaldehyde or retinoid or Retinoids or retinoic or beta-carotene or "beta carotene" or iron or "Fe(III)" or "Fe3+" or "iron(III) " or "Ferrous ion" or "Fe(II)" or "iron(II)" or "Fe2+" or "ferr* compounds" or zinc or "zn" or "zn acetate" or "zn sulfate" or "zn oxide" or iodine or "iod* compounds" or "ferr* compounds" or "folic acid" or "ergocalciferol derivative" or "ergocalciferol-D2" or cholecalciferol-D3 or "colecalciferol derivative" or iodiz* or "beta carotene" or "b-tene" or "beta carotin" or betacarotene).tw,kf
3	Exp Dietary supplements/ or tablets/ or syrup/ or capsules/ or (supplement* or nutraceutical* or nutriceutical* or neutraceutical* or capsule* or tablet* or syrup* or drop*).tw,kf
4	1 AND 2 AND 3
5	**Intervention 2: Lipid-nutrient supplementation**<br><br>exp Lipids/ or (lipid* or oil* or soy* or peanut* or whey* or sesame* or cashew* or chickpea* or protein* or butter* or fat or fats or fatty or "dairy product*" or "omega-3" or "omega 3" or "alpha-linolenic acid" or "docosahexaenoic acids" or "eicosapentaeonic acid" or "n-3 pufa" or "n3 pufa" or glyceride*).tw,kf

6	Dietary supplements/ or ("lipid based" or "lipid-based nutri*" or enrich* or emuls* or "Lipid Emulsions" or "Fat Emulsions" or "Intravenous Fat Emulsions" or powder* or spread* or paste* or LNS or iLiNS or supplement* or neutraceutical* or nutraceutical* or nutriceutical* or Nutributter* or Plumpy* or PlumpyNut or "ready to use" or "ready-to-use therapeutic food" or "ready-to-use supplementary food" or RUFF or RUSF or RUTF).tw,kf
7	1 AND 5 AND 6
8	**Intervention 3: Point of use fortification with micronutrient powders**<br><br>Exp Vitamins/ or exp minerals/ or exp micronutrients/
9	Powders/ or (Sprinkle* or powder* or foodlet* or "foodlet-based" or "crushable nutritabs" or "micronutrient powder" or "multiple micronutrient powder" or mnp or "drug dosage form" or "micronutrient powder*" or "MNP").tw,kf
10	("point-of-use" or enrich* or fortif* or "diet therapy" or supplement* or "diet treatment" or "diet additive" or "dietary therapy" or "dietary treatment" or nutraceutical* or nutriceutical* or neutraceutical*).tw,kf
11	**1 AND 8 AND 9 AND 10**
12	Developing Countries/ or developing country*.tw, kf or ("developing country" or "developing countries" or "developing nation" or "developing nations" or "developing population" or "developing populations" or "developing world" or "less developed country" or "less developed countries" or "less developed nation" or "less developed nations" or "less developed population" or "less developed populations" or "less developed world" or "lesser developed country" or "lesser developed countries" or "lesser developed nation" or "lesser developed nations" or "lesser developed population" or "lesser developed populations" or "lesser developed world" or "under developed country" or "under developed countries" or "under developed nation" or "under developed nations" or "under developed population" or "under developed populations" or "under developed world" or "underdeveloped country" or "underdeveloped countries" or "underdeveloped nation" or "underdeveloped nations" or "underdeveloped population" or "underdeveloped populations" or "underdeveloped world" or "middle income country" or "middle income countries" or "middle income nation" or "middle income nations" or "middle income population" or "middle income populations" or "low income country" or "low income countries" or "low income nation" or "low income nations" or "low income population" or "low income populations" or "lower income country" or "lower income countries" or "lower income nation" or "lower income nations" or "lower income population" or "lower income populations" or "underserved country" or "underserved countries" or "underserved nation" or "underserved nations" or "underserved population" or "underserved populations" or "underserved world" or "under served country" or "under served countries" or "under served nation" or "under served nations" or "under served population" or "under served populations" or "under served world" or "deprived country" or "deprived countries" or "deprived nation" or "deprived nations" or "deprived population" or "deprived populations" or "deprived world" or "poor country" or "poor countries" or "poor nation" or "poor nations" or "poor population" or "poor populations" or "poor world" or "poorer country" or "poorer countries" or "poorer nation" or "poorer nations" or "poorer population" or "poorer populations" or "poorer world" or "developing economy" or "developing economies" or "less developed economy" or "less developed economies" or "lesser developed economy" or "lesser developed economies" or "under developed economy" or "under developed economies" or "underdeveloped economy" or "underdeveloped economies" or "middle income economy" or "middle income economies" or "low income economy" or "low income economies" or "lower income economy" or "lower income economies" or "low gdp" or "low gnp" or "low gross domestic" or "low gross national" or "lower gdp" or "lower gnp" or "lower gross domestic" or "lower gross national" or lmic or lmics or "third world" or "lami country" or "lami countries" or "transitional country" or "transitional countries" or Africa or Asia or Caribbean Region or West Indies or South America or Latin America or Central America or Afghanistan or Albania or Algeria or

	Angola or Argentina or Armenia or Armenian or Azerbaijan or Bangladesh or Benin or Byelarus or Byelorussian or Belarus or Belorussian or Belorussia or Belize or Bhutan or Bolivia or Bosnia or Herzegovina or Hercegovina or Botswana or Brazil or Bulgaria or Burkina Faso or Burkina Fasso or Upper Volta or Burundi or Urundi or Cambodia or Khmer Republic or Kampuchea or Cameroon or CameroonsOR Cameron or Camerons or Cape Verde or Central African Republic or Chad or China or Colombia or Comoros or Comoro Islands or Comores or Mayotte or Congo or Zaire or Costa Rica or Cote d'Ivoire or Ivory Coast or Cuba or Djibouti or French Somaliland or Dominica or Dominican Republic or East Timor or East Timur or Timor Leste or Ecuador or Egypt or United Arab Republic or El Salvador or Eritrea or Ethiopia or Fiji or Gabon or Gabonese Republic or Gambia or Gaza or Georgia Republic or Georgian Republic or Ghana or Gold Coast or Grenada or Guatemala or Guinea or Guiana or Guyana or Haiti or Honduras or India or Maldives or Indonesia or Iran or Iraq or Isle of Man or Jamaica or Jordan or Kazakhstan or Kazakh or Kenya or Kiribati or Korea or Kosovo or Kyrgyzstan or Kirghizia or Kyrgyz Republic or Kirghiz or Kirgizstan or "Lao PDR" or Laos or Lebanon or Lesotho or Basutoland or Liberia or Libya or Macedonia or Madagascar or Malagasy Republic or Malaysia or Malaya or Malay or Sabah or Sarawak or Malawi or Nyasaland or Mali or Marshall Islands or Mauritania or Mauritius or Agalega Islands or Mexico or Micronesia or Middle East or Moldova or Moldovia or Moldovian or Mongolia or Montenegro or Morocco or Ifni or Mozambique or Myanmar or Myanma or Burma or Namibia or Nepal or Nicaragua or Niger or Nigeria or Muscat or Pakistan or Palau or Palestine or Panama or Paraguay or Peru or Philippines or Philipines or Phillipines or Phillippines or Romania or Rumania or Roumania or Russia or Russian or Rwanda or Ruanda or Saint Lucia or St Lucia or Saint Vincent or St Vincent or Grenadines or Samoa or Samoan Islands or Navigator Island or Navigator Islands or Sao Tome or Senegal or Serbia or Montenegro or Seychelles or Sierra Leone or Sri Lanka or Ceylon or Solomon Islands or Somalia or Sudan or Suriname or Surinam or Swaziland or Syria or Tajikistan or Tadzhikistan or Tadjikistan or Tadzhik or Tanzania or Thailand or Togo or Togolese Republic or Tonga or Tunisia or Turkey or Turkmenistan or Turkmen or Uganda or Ukraine or USSR or Soviet Union or Union of Soviet Socialist Republics or Uzbekistan or Uzbek or Vanuatu or New Hebrides or Venezuela or Vietnam or Viet Nam or West Bank or Yemen or Yugoslavia or Zambia or Zimbabwe or Rhodesia).tw,kf
13	4 AND 12
14	7 AND 12
15	11 AND 12
16	13 OR 14 OR 15
17	Limit 16 to yr “1995-current”

Supplementary Materials

Table S2. Summary of Study Characteristics

Author	Year	Country	World Region	Study Design	Sample Size	Included in Analysis	Comparisons	Intervention Received	Comparison/Control Received	Provision of Additional Micronutrients
Ahmad et al.	2015	Bangladesh	South Asia	RCT	56	✓	Zinc vs. placebo	Zinc (20 mg)	Placebo	---
Aminisani et al.	2009	Iran	Middle East & North Africa	RCT	196	✓	Zinc vs. placebo	Zinc (50 mg)	Placebo	IFA (Fe 30 mg, FA 1 mg)
Asemi et al.	2013	Iran	Middle East & North Africa	RCT	48	✓	Vitamin D vs. placebo	Vitamin D (400 IU)	Placebo	IFA (Fe 60 mg, FA 400 ug)
Asemi et al.	2016	Iran	Middle East & North Africa	RCT	46	✓	MMN vs. IFA	Calcium (475-600 mg) + Vitamin D (190-240 IU)	Placebo	IFA (Fe 50 mg, FA 400 ug)
Ashorn et al.	2015	Malawi	Sub-Saharan Africa	RCT	1391	✓	1) MMN vs. IFA<br>2) LNS vs. MMN	1) MMN (20 mg Fe)<br>2) LNS (20 g)	Fe 60 mg, FA 400 ug	---
Belizan et al.	1997	Argentina	Latin America & Caribbean	RCT	1194	✓	Calcium vs placebo	Calcium (500 mg x 4 tablets)	placebo	---
Bhutta et al.	2009	Pakistan	South Asia	cRCT	2378	✓	MMN vs IFA	MMN (Fe 30 mg)	Fe 60 mg, FA 400 ug	---
Castillo-Duran et al.	2001	Chile	Latin America & Caribbean	RCT	804	✓	Zinc vs. placebo	Zinc (20 mg)	Placebo	Fe (40 mg)
Caulfield et al.	1999	Peru	Latin America & Caribbean	RCT	538	✓	1) Zinc vs. placebo<br>2) MMN vs. IFA	MMN (Fe 60 mg, FA 250 ug, Zinc 15 mg)	Fe 60 mg, FA 250 ug	---
Charandabi et al.	2015	Iran	Middle East & North Africa	RCT	126	✓	1) Calcium + Vitamin D vs. placebo<br>2) Vitamin D vs. placebo	1) Calcium (300 mg) + Vitamin D (1000 IU)<br>2) Vitamin D (1000 IU)	Placebo	---
Choudhury et al.	2012	Bangladesh	South Asia	cRCT	478	✓	MMN vs. IFA	MMN (Fe 60 mg)	Fe 60 mg, FA 400 ug	---
Christian et al.	2003	Nepal	South Asia	cRCT	4926	✓	1) IFA vs. FA and Iron vs. placebo<br>2) MMN vs. IFA<br>3) Zinc vs. placebo	1) IFA (Fe 60 mg, FA 400 ug)<br>2) MMN (Fe 60 mg)<br>3) IFA + Zinc (Fe 60 mg, FA 400 ug, Zinc 30 mg)	1) FA (400 ug)<br>2) IFA (Fe 60 mg, FA 400 ug)<br>3) IFA (Fe 60 mg, FA 400 ug)	---
Cox et al.	2005	Ghana	Sub-Saharan Africa	RCT	98	✓	Vitamin A vs. placebo	Vitamin A (10,000 IU)	Placebo	---
Darling et al.	2017	Tanzania	Sub-Saharan Africa	RCT	2500	✓	1) Vitamin A vs. placebo<br>2) Zinc vs. placebo	1) Vitamin A (2500 IU)<br>2) Zinc (25 mg)	Placebo	---
Dewey et al.	2009	Ghana	Sub-Saharan Africa	RCT	1320	✓	1) MMN vs. IFA<br>2) LNS vs. MMN	1) MMN (Fe 20 mg)<br>2) LNS (20 g)	1) IFA (Fe 60 mg, FA 400 ug)<br>2) MMN (Fe 20 mg)	---
Dijkjuizen et al.	2001	Indonesia	East Asia & Pacific	RCT	229	✓	1) MMN vs. IFA<br>2) Zinc vs. placebo	1) MMN (Zinc 30 mg, Fe 30 mg, FA 400 ug)	1) IFA (Fe 30 mg, FA 400 ug)	---
Diogenes et al.	2013	Brazil	Latin America & Caribbean	RCT	84	—	Calcium + Vitamin D vs. placebo	Calcium (600 mg) + Vitmain D (200 IU)	Placebo	---
Duggan et al.	2014	India	South Asia	RCT	366	—	Vitamin B12 vs. placebo	Vitamin B12 (50 ug)	Placebo	IFA (Fe 60 mg, FA 500 ug)
Etheredge et al.	2015	Tanzania	Sub-Saharan Africa	RCT	1500	✓	Iron vs. placebo	Iron (60 mg)	Placebo	FA 5000 ug
Falahi et al.	2010	Iran	Middle East & North Africa	RCT	148	✓	Iron vs. placebo	Iron (60 mg)	Placebo	---
Fawzi et al.	2007	Tanzania	Sub-Saharan Africa	RCT	8428	✓	MMN vs. IFA	MMN	Placebo	IFA (Fe 60 mg, FA 250 ug)
Friis et al.	2004	Zimbabwe	Sub-Saharan Africa	RCT	1669	✓	MMN vs. IFA	MMN	Placebo	IFA (component amounts not reported)
Gowachiarpant et al.	2017	Thailand	East Asia & Pacific	RCT	832	—	Iodine vs. placebo	Iodine (200 ug)	Placebo	---
Hafeez et al.	2005	Pakistan	South Asia	RCT	242	✓	Zinc vs. placebo	Zinc (20 mg)	Placebo	IFA (component amounts not reported)
Hambidge et al.	2019	DRC, Guatemala, India & Pakistan	Multi-country	RCT	7376	—	LNS vs. placebo	LNS	Placebo	---
Hanieh et al.	2013	Vietnam	East Asia & Pacific	cRCT	833	✓	MMN vs. IFA	MMN (Fe 60 mg)	IFA (Fe 60 mg, FA 400 ug)	---
Hossain et al.	2012	Pakistan	South Asia	RCT	200	✓	Vitamin D vs. placebo	Vitamin D (4000 IU)	Placebo	Ferrous sulphate (200 mg) and calcium lactate (600 mg)
Huy et al.	2009	Vietnam	East Asia & Pacific	RCT	1579	✓	MMN vs. IFA	MMN (Fe 30 mg)	IFA (Fe 60 mg, FA 400 ug)	---
Huybregts et al.	2009	Burkina Faso	Sub-Saharan Africa	RCT	1296	✓	LNS vs. MMN	LNS	MMN (Fe 30 mg)	sulfadoxine-pyrimethamine (malaria prevention)
Jarjou et al.	2006	Gambia	Sub-Saharan Africa	RCT	155	—	Calcium vs. placebo	Calcium (1500 mg)	Placebo	---
Khan et al.	2016	Pakistan	South Asia	RCT	85	✓	Vitamin D vs. placebo	Vitamin D (4000 IU)	Placebo	---
Kirkwood et al.	2010	Ghana	Sub-Saharan Africa	cRCT	207781	✓	Vitamin A vs. placebo	Vitamin A (25,000 IU)	Placebo	---
Korkmaz et al.	2014	Turkey	Europe & Central Asia	RCT	108	—	Iron vs. placebo<br>Folic acid vs. placebo	Iron (60 mg)<br>Folic Acid (400 ug)	Placebo	---
Kumar et al.	2009	India	South Asia	RCT	552	✓	Calcium vs. placebo	Calcium (500 mg)	Placebo	---
Kaestel et al.	2005	Guinea-Bissau	Sub-Saharan Africa	RCT	2100	✓	MMN vs. IFA	MMN (Fe 30 mg)	IFA (Fe 60 mg, FA 400 ug)	---
Liu et al.	2013	China	East Asia & Pacific	RCT	18775	✓	1) IFA vs FA and Iron vs. placebo<br>2) MMN vs. IFA	1) IFA (Fe 30 mg, FA 400 ug)<br>2) MMN (Fe 30 mg)	1) FA (400 ug)<br>2) IFA (Fe 30 mg, FA 400 ug)	---
Lopez-Jaramillo et al.	1997	Ecuador	Latin America & Caribbean	RCT	260	✓	Calcium vs. placebo	Calcium (500 mg x 4 tablets)	Placebo	---
Menendez et al.	1995	Gambia	Sub-Saharan Africa	RCT	550	✓	1) Iron vs. placebo<br>2) IFA vs. FA	Iron (60 mg)	Placebo	FA (5000 ug)

Supplementary Materials

Table S2. Summary of Study Characteristics (Cont'd)

Merialdi et al.	2004	Peru	Latin America & Caribbean	RCT	242	✓	1) MMN vs. IFA<br>2) Zinc vs. placebo	MMN (Fe 60 mg, FA 250 ug, Zinc 25 mg)	IFA (Fe 60 mg, FA 250 ug)	---
Moore et al.	2012	Gambia	Sub-Saharan Africa	RCT	875	✓	1) MMN vs. IFA<br>2) LNS vs. MMN	1) MMN (Fe 60 mg)<br>2) LNS	1) IFA (Fe 60 mg, FA 400 ug)<br>2) MMN (Fe 60 mg)	---
Muslimatun et al.	2001	Indonesia	East Asia & Pacific	RCT	243	✓	1) MMN vs. IFA<br>2) Vitamin A vs. placebo	1) MMN (Fe 60 mg, FA 250 ug, Vitamin A 2400 retinol equivalents)	1) IFA (Fe 60 mg, FA 250 ug)	---
Naghshineh et al.	2016	Iran	Middle East & North Africa	RCT	140	✓	Vitamin D vs. placebo	Vitamin D (600 IU)	Placebo	---
Osendarp et al.	2000	Bangladesh	South Asia	RCT	559	✓	Zinc vs. placebo	Zinc (30 mg)	Placebo	---
Osrin et al.	2005	Nepal	South Asia	RCT	1200	✓	MMN vs. IFA	MMN (Fe 30 mg)	IFA (Fe 60 mg, FA 400 ug)	---
Ouladsahebmadarek et al.	2011	Iran	Middle East & North Africa	RCT	960	✓	Iron vs. placebo	Iron (30 mg)	Placebo	multivitamin tablet
Prawirohartono et al.	2011	Indonesia	East Asia & Pacific	RCT	2173	---	1) Vitamin A vs. placebo<br>2) Zinc vs. placebo	1) Vitamin A (2400 IU)<br>2) Zinc (20 mg)	Placebo	IFA (Fe 60 mg, FA 250 ug)
Preziosi et al.	1997	Niger	Sub-Saharan Africa	RCT	197	✓	Iron vs. placebo	Iron (100 mg)	Unclear if placebo was given, or nothing at all	---
Ramakrishnan et al.	2003	Mexico	Latin America & Caribbean	RCT	873	✓	MMN vs. Iron	MMN (Fe 60 mg)	Iron (60 mg)	---
Roberfroid et al.	2008	Burkina Faso	Sub-Saharan Africa	RCT	1426	✓	MMN vs. IFA	MMN (Fe 30 mg)	IFA (Fe 60 mg, FA 400 ug)	---
Roth et al.	2013	Bangladesh	South Asia	RCT	160	✓	1) Vitamin D vs. placebo<br>2) MMN vs. IFA	Vitamin D (35,000 IU)	Placebo	IFA (Fe 66 mg, FA 350 ug)
Roth et al.	2018	Bangladesh	South Asia	RCT	1300	✓	1) Vitamin D vs. placebo<br>2) MMN vs. IFA	Vitamin D (35,000 IU)	Placebo	IFA (Fe 66 mg, FA 350 ug)
Saaka et al.	2009	Ghana	Sub-Saharan Africa	RCT	600	✓	MMN vs. IFA	MMN (Fe 40 mg, Zinc 40 mg)	Placebo	FA 400 ug
Sabet et al.	2012	Iran	Middle East & North Africa	RCT	50	✓	Vitamin D vs. placebo	Vitamin D (100,000 IU 3 times per month)	Placebo	---
Sablok et al.	2015	India	South Asia	RCT	180	✓	Vitamin D vs. placebo	Vitamin D (60,000 IU if vitamin D sufficient; 120,000 IU if vitamin D deficient)	Placebo	---
Sahu et al.	2009	India	South Asia	RCT	139	✓	Vitamin D vs. placebo	Vitamin D (120,000 IU twice during pregnancy)	Placebo	---
Semba et al.	2001	Malawi	Sub-Saharan Africa	RCT	203	✓	1) MMN vs. IFA<br>2) Vitamin A vs. placebo	MMN (Vitamin A 10,000IU, Fe 30 mg, FA 400 ug)	IFA (Fe 30 mg, FA 400 ug)	---
Sorouri et al.	2016	Iran	Middle East & North Africa	RCT	540	✓	1) MMN vs. IFA<br>2) Zinc vs. placebo	MMN (Zinc 15 mg)	Placebo	IFA (Fe 30 mg, FA 400 ug)
SUMMIT et al.	2008	Indonesia	East Asia & Pacific	cRCT	31290	✓	MMN vs. IFA	MMN (Fe 30mg)	IFA (Fe 30 mg, FA 400 ug)	---
Sunawang et al.	2009	Indonesia	East Asia & Pacific	cRCT	843	✓	MMN vs. IFA	MMN (Fe 30 mg)	IFA (Fe 60 mg, FA 250 ug)	---
Taherian et al.	2002	Iran	Middle East & North Africa	RCT	990	---	Calcium + Vitamin D vs. placebo	Calcium (500mg) + Vitamin D (200 IU)	Placebo	---
Tanumihardjo et al.	2002	Indonesia	East Asia & Pacific	RCT	27	✓	1) Iron vs. placebo<br>2) Vitamin A vs. placebo	1) Iron (60 mg)<br>2) Vitamin A (8000 IU)	Placebo	---
Tofail et al.	2008	Bangladesh	South Asia	RCT	2472	✓	MMN vs. IFA	MMN (Fe 30mg)	IFA (Fe 30 mg, FA 400 ug)	---
Vaziri et al.	2016	Iran	Middle East & North Africa	RCT	127	✓	Vitamin D vs. placebo	Vitamin D (2000 IU)	Placebo	---
Villar et al.	2006	India, Peru, South Africa & Vietnam	Multi-country	RCT	8325	✓	1) Calcium vs. placebo<br>2) MMN vs. IFA	Calcium (1500 mg)	Placebo	IFA (component amounts not reported)
West et al.	1999	Nepal	South Asia	cRCT	30110	✓	Vitamin A vs. Placebo	Vitamin A (23,300 IU)	Placebo	5mg of dl-alpha-tocopherol as an antioxidant
West et al.	2011	Bangladesh	South Asia	cRCT	39632	✓	Vitamin A vs. placebo	Vitamin A (23,300 IU)	Placebo	---
West et al.	2014	Bangladesh	South Asia	cRCT	45000	✓	MMN vs. IFA	MMN (Fe 27 mg)	IFA (Fe 27mg, FA 600 ug)	---
Zagré et al.	2007	Niger	Sub-Saharan Africa	cRCT	3670	✓	MMN vs. IFA	MMN (Fe 30 mg)	IFA (Fe 60 mg, FA 400 ug)	---
Zeng et al.	2008	China	East Asia & Pacific	cRCT	3811	✓	1) IFA vs. FA and Iron vs. Placebo<br>2) MMN vs. IFA	1) IFA (Fe 60 mg, FA 400 ug)<br>2) MMN (Fe 30 mg)	1) FA (400 ug)<br>2) IFA (Fe 60 mg, FA 400 ug)	---
Zhao et al.	2015	China	East Asia & Pacific	RCT	2371	✓	IFA vs. FA and Iron vs. Placebo	IFA (Fe 60 mg, FA 400 ug)	FA (400 ug)	---
Ziaei et al.	2007	Iran	Middle East & North Africa	RCT	750	✓	Iron vs. Placebo and IFA vs. FA	Iron (50 mg)	Placebo	FA (1000 ug)
Ziaei et al.	2008	Iran	Middle East & North Africa	RCT	244	✓	Iron vs. placebo	Iron (50 mg)	Placebo	

Fe , Iron; FA, folic acid; MMN, multiple micronutrient; IFA, iron-folic acid; LNS, lipid-based nutrient supplement

Supplementary Materials

Table S3. Descriptive Summary of Additional Studies (not included in meta-analyses)

Author	Year	Country	World Region	Study Design	Sample Size	Reason for exclusion from meta-analysis	Intervention Received	Comparison/Control Received	Provision of Additional Micronutrients	Outcomes Reported & Findings
Diogenes et al.	2013	Brazil	Latin America & the Caribbean	RCT	84	No outcomes reported that could be pooled in a meta-analysis	Calcium plus vitamin D	Placebo	Iron Folic Acid	Outcomes: maternal bone measurements, total bone mineral content, total bone mineral density, serum vitamin D concentration.<br><br>Serum vitamin D concentration levels in mothers who received supplementation were higher than those in mothers in the placebo group. The authors postulated an effect of increased calcium intake during pregnancy on reduced postpartum bone loss in mothers.
Duggan et al.	2014	India	South Asia	RCT	366	Insufficient number of studies to form a meta-analysis	Vitamin B12	Placebo	Iron Folic Acid	Outcomes: mode of delivery - Caesarean section, low birthweight, mean birth weight, infants with intrauterine growth retardation, maternal vitamin B12 concentrations (plasma), vitamin B12 concentrations in breastmilk<br><br>Women in the intervention group had higher vitamin B12 plasma concentrations, and higher vitamin B12 concentrations in their breastmilk, than women in the placebo group. Mean birth weight did not differ significanty between intervention and control groups. In terms of IUGR incidence, 25% of mothers in the vitamin B12 group delivered an infant with IUGR, compared to 34% of mothers in the placerbo group (P=0.11)
Gowachirapant et al.	2017	India<br>Thailand	South Asia<br>East Asia & Pacific	RCT	832	Insufficient number of studies to form a meta-analysis	Iodine	Placebo	None	Outcomes: child developmental outcomes (verbal and performance IQ scores), child's auditory performance, child weight, child height, maternal urinary iodine concentration, maternal thyroid stimulating hormone (TSH) concentration, maternal total thyroxine (T4) concentration.<br><br>Authors noted no significant differences between the intervention and control groupin terms of any child development or health outcomes. Maternal urinary iodine concentration was higher in the iodine group than in the placebo group during pregnancy. These differences disappeared at 6-weeks postpartum. In terms of TSH and T4 levels in mothers, no significant differences were observed between the iodine and control groups.
Hambidge et al.	2019	Democratic Republic of Congo<br>Guatemala<br>India<br>Pakistan	Sub-Saharan Africa<br>Latin America & the Caribbean<br>South Asia<br>South Asia	RCT	7376	Insufficient number of studies to form a meta-analysis	LNS	Placebo	None	Outcomes: length-for-age (primary outcome), weight, head circumference, BMI, WAZ, HC-for-age, BMI for age, low birthweight, small-for-gestational age, preterm births<br><br>Authors noted no significant difference in length-for-age z scores between the two intervention arms in all sites; however, a small effect was observed in intervention group 1 compared to the placebo group in Guatemala.
Jarjou et al.	2006	Gambia	Sub-Saharan Africa	RCT	155	No outcomes reported that could be pooled in a meta-analysis	Calcium	Placebo	None	Outcomes: maternal vitamin D serum concentration, bone mineral accretion in infants, birthweight (grams), blood pressure, urine biomarkers and calcium concentration in breastmilk<br><br>Authors noted no significant differences between the intervention and control groups in terms of breastmilk concentrations of calcium, infant birth weight, infant growth or bone mineral status during the first year of life
Korkmaz et al.	2014	Turkey	Europe & Central Asia	RCT	108	No outcomes reported that could be pooled in a meta-analysis	Iron	Folate or Placebo	None	Outcomes: maternal weight, weight gain during pregnancy, serum albumin levels, oxidative stress markers (γ-glutamyl transferase)<br><br>Authors noted that the oxidative stress markers were significantly higher in the intervention group compared to placebo ($P<0.001$). Maternal weight and weight gain during pregnancy was lower in the intervention group (iron) compared to the folate intervention group and the placebo group ($P<0.05$). Mean serum albumin concentrations were similar across all groups.
Prawirohartono et al.	2011	Indonesia	East Asia & Pacific	RCT	2173	No outcomes reported that could be pooled in a meta-analysis	Vitamin A only<br>Zinc only<br>Vitamin A & Zinc combined	Placebo	None	Outcomes: weight-for-age (WAZ), height-for-age (HAZ), weight-for-height (WHZ); low birthweight, small-for-gestational age, preterm births<br><br>The study findings showed no effect of vitamin A or zinc, or combined vitamin or zinc, compared to placebo, on WAZ, HAZ and WHZ. For secondary outcomes (low birthweight, small-for-gestational age, preterm births), no significant differences were found between the intervention and control groups
Taherian et al.	2002	Iran	Middle East & North Africa	RCT	990	No outcomes reported that could be pooled in a meta-analysis	Calcium plus vitamin D	Placebo	None	Outcomes: pre-eclampsia<br><br>Authors noted a reduction in the prevalence of pre-eclampsia in women who received either a low-dose aspirin daily or only calcium-vitamin D supplements, compared to placebo. Between the low-dose aspirin group and the calcium-vitamin D group, no differences were observed in terms of pre-eclampsia

Supplementary Materials

Figure S1.

<u>Comparison: Vitamin A Supplementation vs. Placebo/No Intervention</u>

Maternal Mortality

Study or Subgroup	Vitamin A Events	Vitamin A Total	Placebo Events	Placebo Total	Weight	Risk Ratio M-H, Random, 95% CI
Kirkwood 2010	138	39601	148	39234	53.8%	0.92 [0.73, 1.16]
West 1999	24	6402	36	5984	21.2%	0.62 [0.37, 1.04]
West 2011	39	16369	34	16412	25.1%	1.15 [0.73, 1.82]
**Total (95% CI)**		**62372**		**61630**	**100.0%**	**0.90 [0.68, 1.18]**
Total events	201		218			

Heterogeneity: Tau² = 0.02; Chi² = 3.09, df = 2 (P = 0.21); I² = 35%

Test for overall effect: Z = 0.78 (P = 0.44)

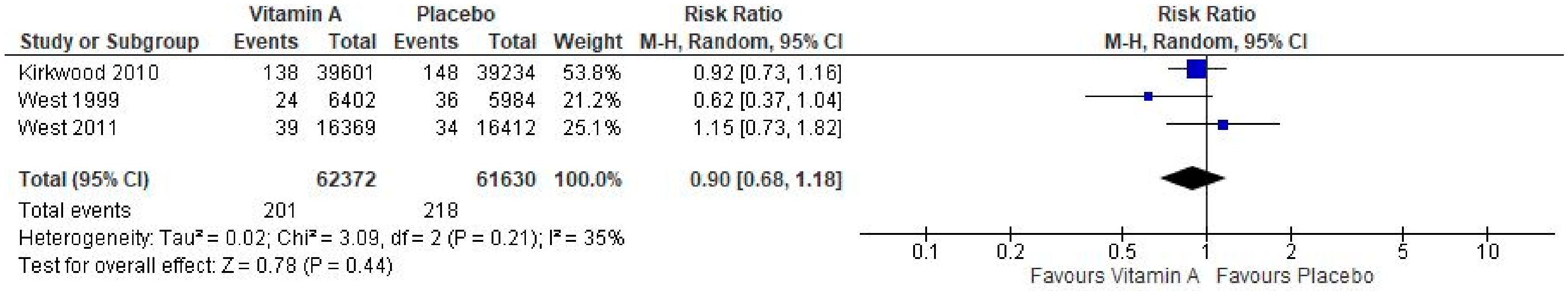

Stillbirths

Study or Subgroup	Vitamin A Events	Vitamin A Total	Placebo Events	Placebo Total	Weight	Risk Ratio M-H, Random, 95% CI
Kirkwood 2010 (1)	1241	38283	1183	37893	49.7%	1.04 [0.96, 1.12]
West 1999 (2)	524	5252	469	4800	21.8%	1.02 [0.91, 1.15]
West 2011 (3)	665	14450	703	14545	28.5%	0.95 [0.86, 1.06]
**Total (95% CI)**		**57985**		**57238**	**100.0%**	**1.01 [0.96, 1.07]**
Total events	2430		2355			

Heterogeneity: Tau² = 0.00; Chi² = 1.76, df = 2 (P = 0.42); I² = 0%

Test for overall effect: Z = 0.33 (P = 0.74)

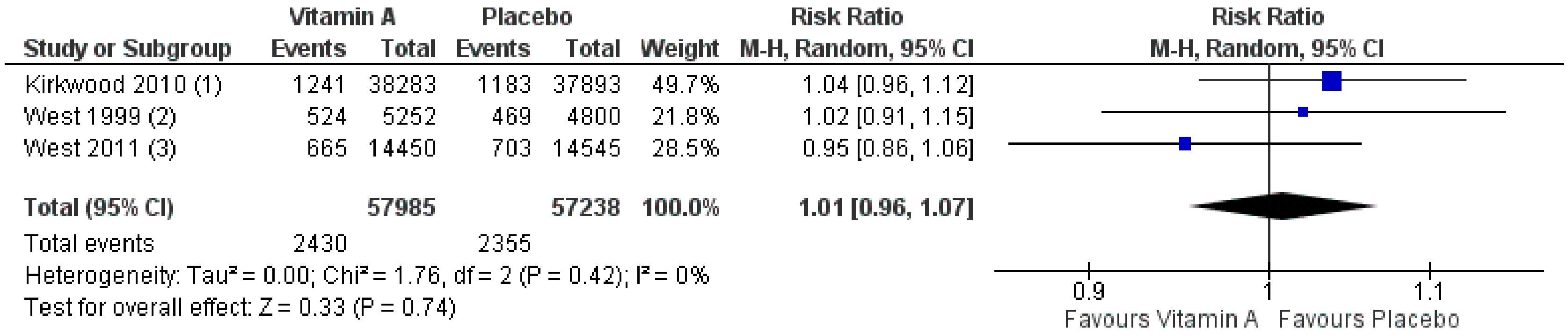

<u>Footnotes</u>

(1) *denominator calculated by adding # of stillbirths + # of pregnancies with live births (Deliveries)

(2) *denominator calculated by adding # of stillbirths + # of deliveries

(3) *denominator calculated by adding # of stillbirths + # of pregnancies with live births (Deliveries)

Supplementary Materials

Maternal serum/plasma hemoglobin concentration (umol/L)

	Vitamin A			Placebo				Mean Difference
Study or Subgroup	Mean	SD	Total	Mean	SD	Total	Weight	IV, Random, 95% CI
Cox 2005	113	11.7092	48	112.2	15.1304	50	19.1%	0.80 [-4.54, 6.14]
Darling 2017	104	23	661	106	22	671	38.8%	-2.00 [-4.42, 0.42]
Muslimatun 2001	114	11.8	71	110.6	10.92	53	26.4%	3.40 [-0.62, 7.42]
Semba 2001 (1)	4.7	36.5	63	7.3	16.59	52	7.3%	-2.60 [-12.68, 7.48]
Tanumihardjo 2002	109	6	7	104	11	7	8.4%	5.00 [-4.28, 14.28]
**Total (95% CI)**			**850**			**833**	**100.0%**	**0.51 [-2.42, 3.43]**

Heterogeneity: Tau² = 4.23; Chi² = 6.76, df = 4 (P = 0.15); I² = 41%

Test for overall effect: Z = 0.34 (P = 0.74)

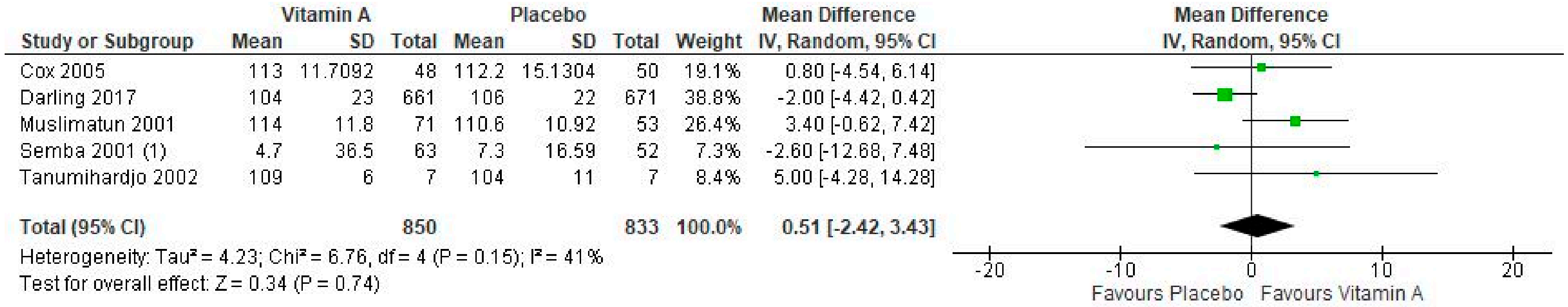

Footnotes

(1) SEM converted to SD; change score from enrollment to delivery was used

Supplementary Materials

Comparison: Zinc Supplementation vs. Placebo/No Intervention

Low birthweight

Study or Subgroup	Zinc Events	Zinc Total	Placebo Events	Placebo Total	Weight	Risk Ratio M-H, Random, 95% CI
**5.1.1 Zinc vs. Placebo (additional micronutrients)**						
Aminisani 2009	0	87	6	88	0.2%	0.08 [0.00, 1.36]
Castillo-Durán 2001	6	249	16	258	2.3%	0.39 [0.15, 0.98]
Caulfield 1999	17	488	17	469	4.3%	0.96 [0.50, 1.86]
Christian 2003	332	588	287	556	50.9%	1.09 [0.98, 1.22]
Dijkhuizen 2001	7	42	4	38	1.5%	1.58 [0.50, 4.99]
Hafeez 2005	15	121	11	121	3.5%	1.36 [0.65, 2.85]
Saaka 2009	26	272	21	271	6.0%	1.23 [0.71, 2.14]
Sorouri 2016	17	263	19	265	4.7%	0.90 [0.48, 1.70]
**Subtotal (95% CI)**		**2110**		**2066**	**73.4%**	**1.02 [0.80, 1.31]**
Total events	420		381			
Heterogeneity: Tau² = 0.03; Chi² = 9.73, df = 7 (P = 0.20); I² = 28%						
Test for overall effect: Z = 0.19 (P = 0.85)						
**5.1.2 Strictly Zinc vs. Placebo**						
Ahmad 2015	4	23	2	24	0.8%	2.09 [0.42, 10.32]
Osendarp 2000	89	194	87	216	25.8%	1.14 [0.91, 1.42]
**Subtotal (95% CI)**		**217**		**240**	**26.6%**	**1.15 [0.92, 1.44]**
Total events	93		89			
Heterogeneity: Tau² = 0.00; Chi² = 0.55, df = 1 (P = 0.46); I² = 0%						
Test for overall effect: Z = 1.26 (P = 0.21)						
**Total (95% CI)**		**2327**		**2306**	**100.0%**	**1.08 [0.94, 1.25]**
Total events	513		470			
Heterogeneity: Tau² = 0.01; Chi² = 10.46, df = 9 (P = 0.31); I² = 14%						
Test for overall effect: Z = 1.12 (P = 0.26)						
Test for subgroup differences: Chi² = 0.49, df = 1 (P = 0.48), I² = 0%						

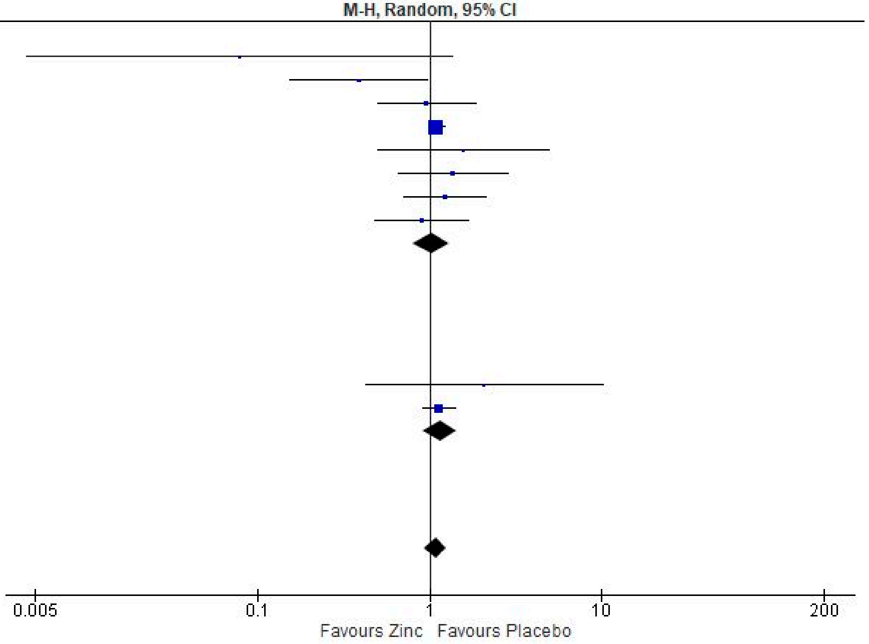

Preeclampsia/eclampsia

Study or Subgroup	Zinc Events	Zinc Total	Placebo Events	Placebo Total	Weight	Risk Ratio M-H, Random, 95% CI
Aminisani 2009 (1)	3	89	5	90	21.3%	0.61 [0.15, 2.46]
Castillo-Durán 2001	2	249	2	258	11.0%	1.04 [0.15, 7.30]
Sorouri 2016	13	270	11	270	67.8%	1.18 [0.54, 2.59]
**Total (95% CI)**		**608**		**618**	**100.0%**	**1.01 [0.53, 1.93]**
Total events	18		18			
Heterogeneity: Tau² = 0.00; Chi² = 0.66, df = 2 (P = 0.72); I² = 0%						
Test for overall effect: Z = 0.03 (P = 0.97)						

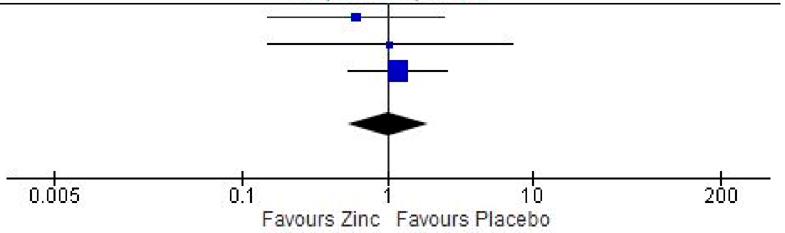

Footnotes

(1) *this includes eclampsia cases

Supplementary Materials
Preterm births

Study or Subgroup	Zinc Events	Zinc Total	Placebo Events	Placebo Total	Weight	Risk Ratio M-H, Random, 95% CI
**5.4.1 Zinc vs. Placebo (additional micronutrients)**						
Aminisani 2009	9	89	7	90	3.9%	1.30 [0.51, 3.34]
Castillo-Durán 2001	14	249	30	258	8.3%	0.48 [0.26, 0.89]
Caulfield 1999	29	521	30	495	11.5%	0.92 [0.56, 1.51]
Christian 2003	127	628	137	593	29.1%	0.88 [0.71, 1.08]
Dijkhuizen 2001	3	48	4	42	1.8%	0.66 [0.16, 2.77]
Hafeez 2005 (1)	22	121	10	121	6.5%	2.20 [1.09, 4.45]
Merialdi 2004	7	109	5	113	2.8%	1.45 [0.47, 4.44]
Saaka 2009	40	272	39	271	15.1%	1.02 [0.68, 1.54]
Sorouri 2016	14	270	15	270	6.4%	0.93 [0.46, 1.90]
**Subtotal (95% CI)**		**2307**		**2253**	**85.4%**	**0.96 [0.76, 1.21]**
Total events	265		277			
Heterogeneity: Tau² = 0.04; Chi² = 12.00, df = 8 (P = 0.15); I² = 33%						
Test for overall effect: Z = 0.37 (P = 0.71)						
**5.4.2 Strictly Zinc vs. Placebo**						
Ahmad 2015	1	23	2	24	0.7%	0.52 [0.05, 5.37]
Osendarp 2000	34	194	34	216	13.9%	1.11 [0.72, 1.72]
**Subtotal (95% CI)**		**217**		**240**	**14.6%**	**1.09 [0.71, 1.66]**
Total events	35		36			
Heterogeneity: Tau² = 0.00; Chi² = 0.39, df = 1 (P = 0.53); I² = 0%						
Test for overall effect: Z = 0.38 (P = 0.71)						
**Total (95% CI)**		**2524**		**2493**	**100.0%**	**0.97 [0.80, 1.17]**
Total events	300		313			
Heterogeneity: Tau² = 0.02; Chi² = 12.87, df = 10 (P = 0.23); I² = 22%						
Test for overall effect: Z = 0.35 (P = 0.73)						
Test for subgroup differences: Chi² = 0.26, df = 1 (P = 0.61), I² = 0%						

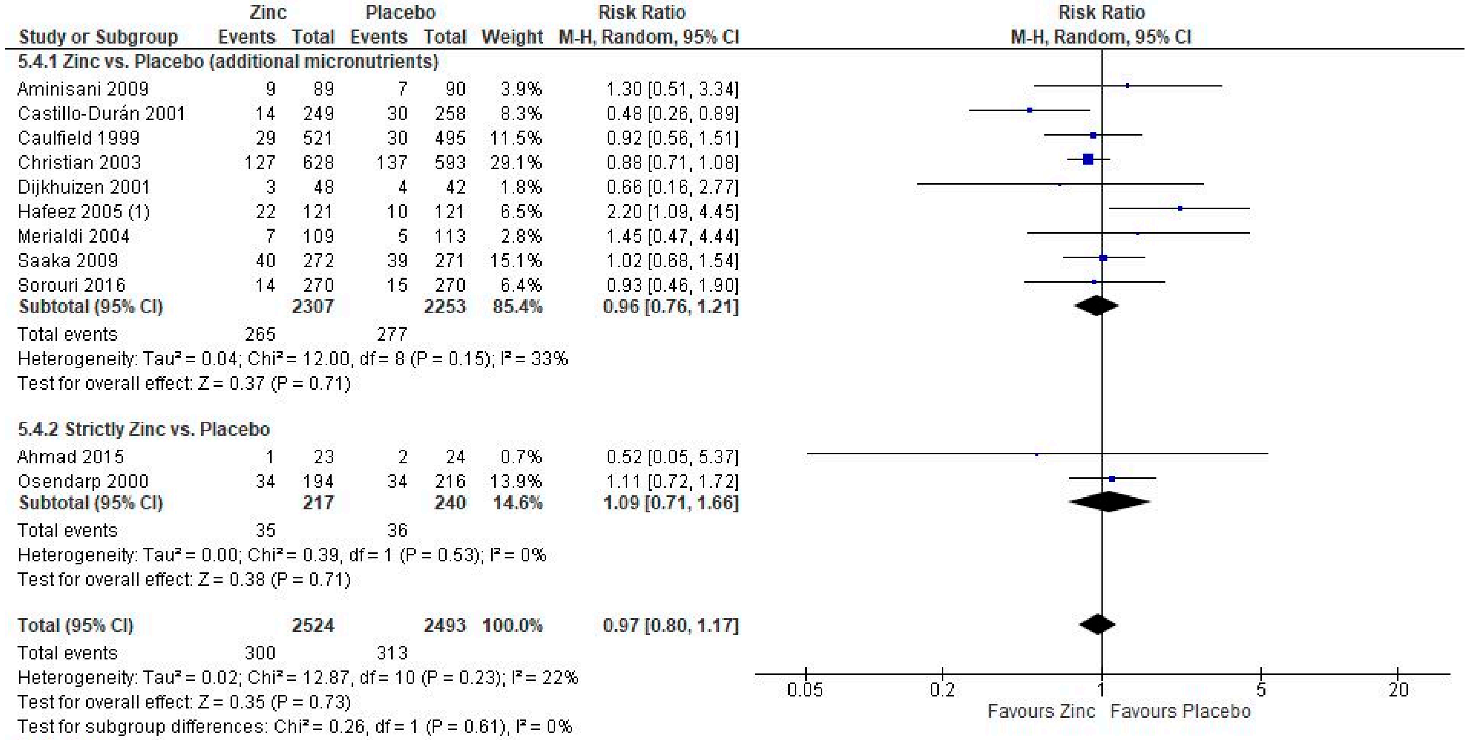

Footnotes
(1) *N event values for control and intervention group were rounded

Supplementary Materials

Small-for-gestational age babies

Study or Subgroup	Zinc Events	Zinc Total	Placebo Events	Placebo Total	Weight	Risk Ratio M-H, Random, 95% CI
**5.5.1 Zinc vs. Placebo (additional micronutrients)**						
Christian 2003	354	628	306	593	53.5%	1.09 [0.98, 1.21]
Saaka 2009	14	272	18	271	1.3%	0.77 [0.39, 1.53]
**Subtotal (95% CI)**		**900**		**864**	**54.7%**	**1.08 [0.98, 1.20]**
Total events	368		324			
Heterogeneity: Tau² = 0.00; Chi² = 0.99, df = 1 (P = 0.32); I² = 0%						
Test for overall effect: Z = 1.53 (P = 0.12)						
**5.5.2 Strictly Zinc vs. Placebo**						
Osendarp 2000	145	194	161	216	45.3%	1.00 [0.90, 1.12]
**Subtotal (95% CI)**		**194**		**216**	**45.3%**	**1.00 [0.90, 1.12]**
Total events	145		161			
Heterogeneity: Not applicable						
Test for overall effect: Z = 0.05 (P = 0.96)						
**Total (95% CI)**		**1094**		**1080**	**100.0%**	**1.05 [0.97, 1.13]**
Total events	513		485			
Heterogeneity: Tau² = 0.00; Chi² = 1.98, df = 2 (P = 0.37); I² = 0%						
Test for overall effect: Z = 1.17 (P = 0.24)						
Test for subgroup differences: Chi² = 0.99, df = 1 (P = 0.32), I² = 0%						

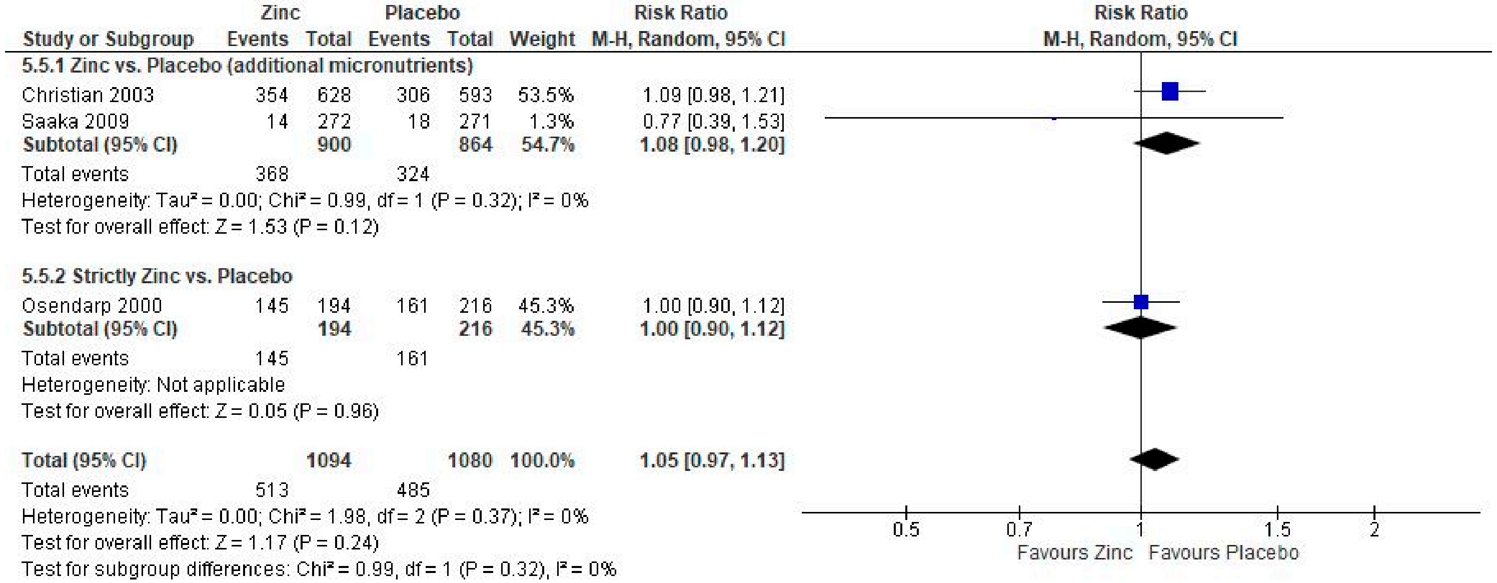

Supplementary Materials

Subgroup analysis: Low birthweight babies (geographical region)

Study or Subgroup	Zinc Events	Zinc Total	Placebo Events	Placebo Total	Weight	Risk Ratio M-H, Random, 95% CI
**10.2.1 South-East Asia**						
Ahmad 2015	4	23	2	24	0.3%	2.09 [0.42, 10.32]
Christian 2003	332	588	287	556	77.1%	1.09 [0.98, 1.22]
Dijkhuizen 2001	7	42	4	38	0.7%	1.58 [0.50, 4.99]
Hafeez 2005	15	121	11	121	1.6%	1.36 [0.65, 2.85]
Osendarp 2000	89	194	87	216	17.9%	1.14 [0.91, 1.42]
**Subtotal (95% CI)**		**968**		**955**	**97.7%**	**1.11 [1.01, 1.22]**
Total events	447		391			
Heterogeneity: Tau² = 0.00; Chi² = 1.42, df = 4 (P = 0.84); I² = 0%						
Test for overall effect: Z = 2.17 (P = 0.03)						
**10.2.2 Eastern Mediterranean**						
Aminisani 2009	0	87	6	88	0.1%	0.08 [0.00, 1.36]
Sorouri 2016	17	263	19	265	2.2%	0.90 [0.48, 1.70]
**Subtotal (95% CI)**		**350**		**353**	**2.3%**	**0.39 [0.04, 4.18]**
Total events	17		25			
Heterogeneity: Tau² = 2.14; Chi² = 2.92, df = 1 (P = 0.09); I² = 66%						
Test for overall effect: Z = 0.78 (P = 0.43)						
**Total (95% CI)**		**1318**		**1308**	**100.0%**	**1.10 [1.00, 1.21]**
Total events	464		416			
Heterogeneity: Tau² = 0.00; Chi² = 5.13, df = 6 (P = 0.53); I² = 0%						
Test for overall effect: Z = 2.04 (P = 0.04)						
Test for subgroup differences: Chi² = 0.75, df = 1 (P = 0.39), I² = 0%						

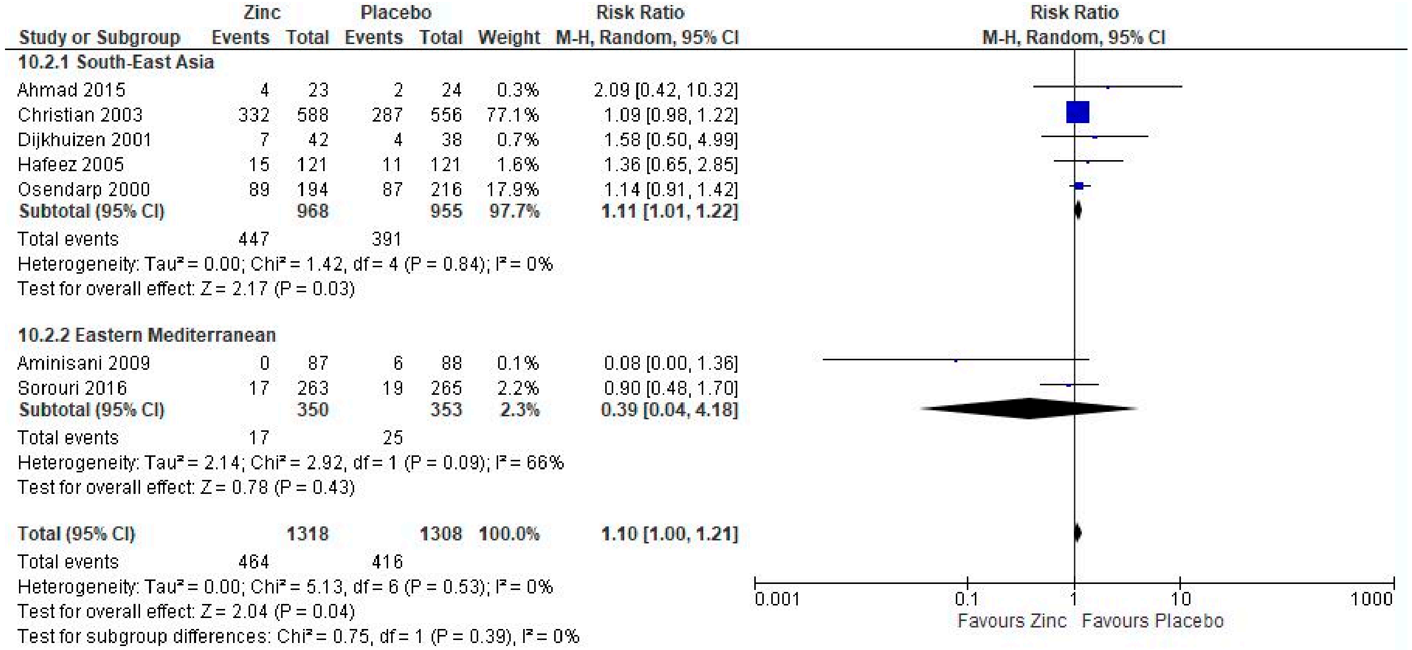

Supplementary Materials

Comparison: Iron Supplementation vs. Placebo/No Intervention

Maternal hemoglobin concentration (g/L)

	Iron			Placebo				Mean Difference
**Study or Subgroup**	**Mean**	**SD**	**Total**	**Mean**	**SD**	**Total**	**Weight**	**IV, Random, 95% CI**
**6.6.1 Iron vs. Placebo (additional micronutrients)**								
Christian 2003	-1.6	20.3	149	-14.4	19.4	146	8.6%	12.80 [8.27, 17.33]
Etheredge 2015	118	20	481	109	19	501	9.4%	9.00 [6.56, 11.44]
Liu 2013	122	9	5913	122	9	5896	9.8%	0.00 [-0.32, 0.32]
Menendez 1995	103.733	15.1809	199	95.622	16	196	9.2%	8.11 [5.03, 11.19]
Ouladsahebmadarek 2011	134.6	7.5	410	124.8	9.1	372	9.7%	9.80 [8.62, 10.98]
Zeng 2008	110.1	14.8	149	105.3	14.2	168	9.2%	4.80 [1.60, 8.00]
Zhao 2015	122	14.6	814	117	7.2	802	9.7%	5.00 [3.88, 6.12]
Ziaei 2007	137.5	10.5	370	125.6	12.4	357	9.6%	11.90 [10.23, 13.57]
**Subtotal (95% CI)**			**8485**			**8438**	**75.2%**	**7.59 [3.43, 11.75]**
Heterogeneity: Tau² = 34.46; Chi² = 540.98, df = 7 (P < 0.00001); I² = 99%								
Test for overall effect: Z = 3.58 (P = 0.0003)								
**6.6.2 Strictly Iron vs. Placebo**								
Falahi 2010	123.2	8.8	70	120.9	7.9	78	9.3%	2.30 [-0.41, 5.01]
Tanumihardjo 2002	118	6.7	5	104	11	7	5.7%	14.00 [3.96, 24.04]
Ziaei 2008	138.8	4.5	100	127.8	4.7	105	9.7%	11.00 [9.74, 12.26]
**Subtotal (95% CI)**			**175**			**190**	**24.8%**	**8.38 [1.16, 15.60]**
Heterogeneity: Tau² = 33.90; Chi² = 33.41, df = 2 (P < 0.00001); I² = 94%								
Test for overall effect: Z = 2.27 (P = 0.02)								
**Total (95% CI)**			**8660**			**8628**	**100.0%**	**7.80 [4.08, 11.52]**
Heterogeneity: Tau² = 36.63; Chi² = 749.82, df = 10 (P < 0.00001); I² = 99%								
Test for overall effect: Z = 4.11 (P < 0.0001)								
Test for subgroup differences: Chi² = 0.03, df = 1 (P = 0.85), I² = 0%								

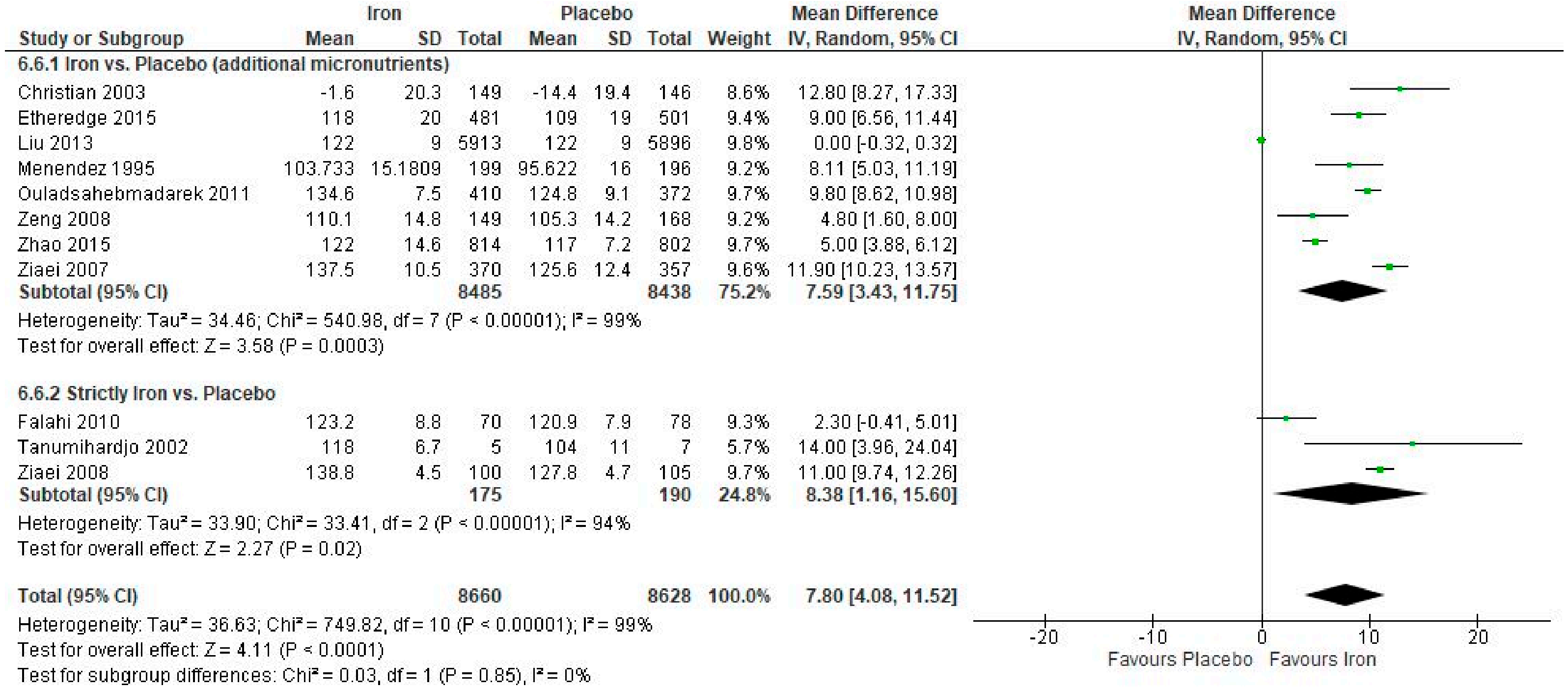

Supplementary Materials

Maternal serum/plasma ferritin concentration (ug/L)

	Iron			Placebo				Mean Difference
Study or Subgroup	Mean	SD	Total	Mean	SD	Total	Weight	IV, Random, 95% CI
**6.7.1 Iron vs. Placebo (additional micronutrients)**								
Christian 2003	2.9	17.2	127	-6.7	20.1	126	12.3%	9.60 [4.99, 14.21]
Etheredge 2015	92.5	171.1	483	54.1	90.3	509	10.3%	38.40 [21.24, 55.56]
Liu 2013	16.7	11.8577	278	11.3	7.6779	282	12.4%	5.40 [3.74, 7.06]
Menendez 1995	103.57	8.7721	237	63.179	5.8112	240	12.4%	40.39 [39.05, 41.73]
Ouladsahebmadarek 2011	269.1	427.2	410	92.6	119.6	372	5.4%	176.50 [133.40, 219.60]
Zhao 2015	15.3	11.6281	814	11.1	7.2136	802	12.4%	4.20 [3.26, 5.14]
**Subtotal (95% CI)**			**2349**			**2331**	**65.2%**	**32.87 [15.39, 50.34]**
Heterogeneity: Tau² = 420.90; Chi² = 2081.12, df = 5 (P < 0.00001); I² = 100%								
Test for overall effect: Z = 3.69 (P = 0.0002)								
**6.7.2 Strictly Iron vs. Placebo**								
Falahi 2010	28.1	18.8	70	22.1	17.3	78	12.1%	6.00 [0.16, 11.84]
Tanumihardjo 2002	43.8	18.3	5	27.6	8.6	7	10.3%	16.20 [-1.06, 33.46]
Ziaei 2008	26.18	11.17	100	19.08	10.71	105	12.4%	7.10 [4.10, 10.10]
**Subtotal (95% CI)**			**175**			**190**	**34.8%**	**7.09 [4.45, 9.72]**
Heterogeneity: Tau² = 0.00; Chi² = 1.20, df = 2 (P = 0.55); I² = 0%								
Test for overall effect: Z = 5.27 (P < 0.00001)								
**Total (95% CI)**			**2524**			**2521**	**100.0%**	**24.14 [10.83, 37.45]**
Heterogeneity: Tau² = 370.94; Chi² = 2108.94, df = 8 (P < 0.00001); I² = 100%								
Test for overall effect: Z = 3.55 (P = 0.0004)								
Test for subgroup differences: Chi² = 8.17, df = 1 (P = 0.004), I² = 87.8%								

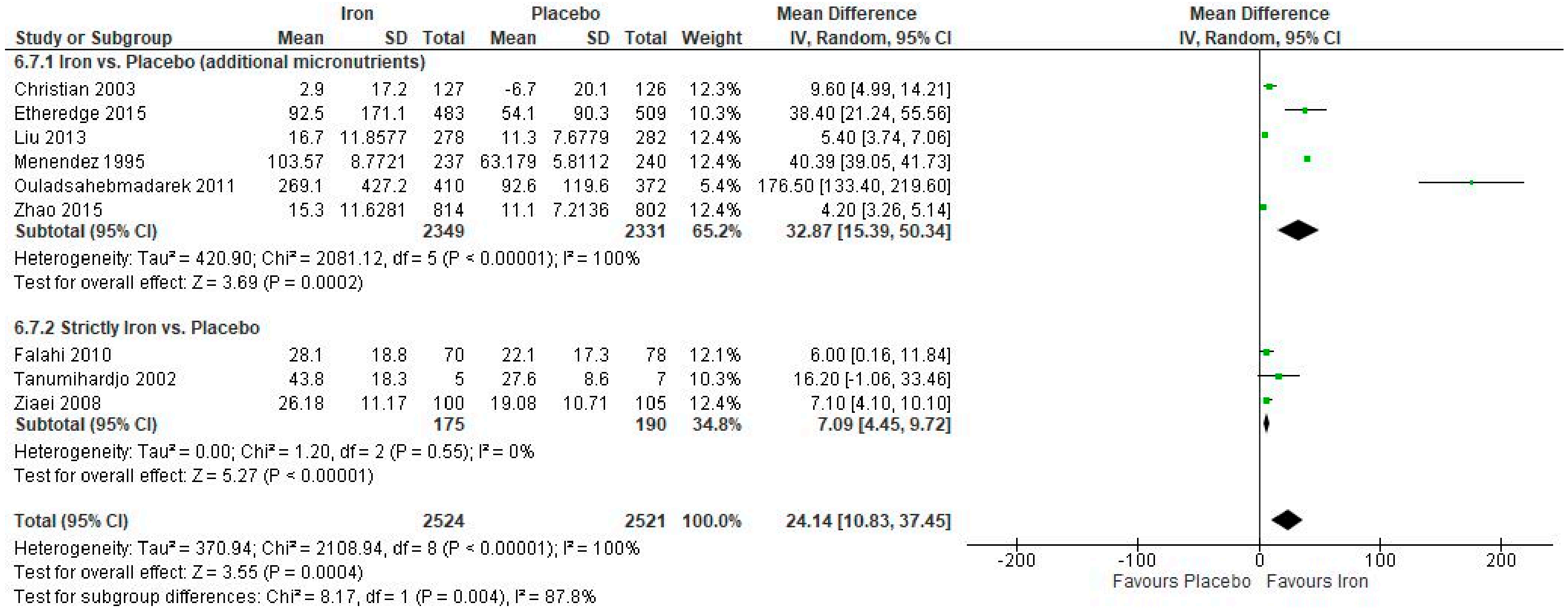

Supplementary Materials

Maternal iron deficiency

Study or Subgroup	Iron Events	Iron Total	Placebo Events	Placebo Total	Weight	Risk Ratio M-H, Random, 95% CI
**6.9.1 Iron vs. Placebo (additional micronutrients)**						
Liu 2013 (1)	98	278	168	282	32.8%	0.59 [0.49, 0.71]
Zhao 2015 (2)	462	815	618	802	36.6%	0.74 [0.69, 0.79]
**Subtotal (95% CI)**		**1093**		**1084**	**69.4%**	**0.67 [0.54, 0.83]**
Total events	560		786			
Heterogeneity: Tau² = 0.02; Chi² = 4.78, df = 1 (P = 0.03); I² = 79%						
Test for overall effect: Z = 3.64 (P = 0.0003)						
**6.9.2 Strictly Iron vs. Placebo**						
Falahi 2010 (3)	7	70	22	78	10.8%	0.35 [0.16, 0.78]
Preziosi 1997 (4)	17	99	50	98	19.7%	0.34 [0.21, 0.54]
**Subtotal (95% CI)**		**169**		**176**	**30.6%**	**0.34 [0.23, 0.51]**
Total events	24		72			
Heterogeneity: Tau² = 0.00; Chi² = 0.01, df = 1 (P = 0.91); I² = 0%						
Test for overall effect: Z = 5.19 (P < 0.00001)						
**Total (95% CI)**		**1262**		**1260**	**100.0%**	**0.54 [0.40, 0.74]**
Total events	584		858			
Heterogeneity: Tau² = 0.07; Chi² = 18.40, df = 3 (P = 0.0004); I² = 84%						
Test for overall effect: Z = 3.91 (P < 0.0001)						
Test for subgroup differences: Chi² = 8.32, df = 1 (P = 0.004), I² = 88.0%						

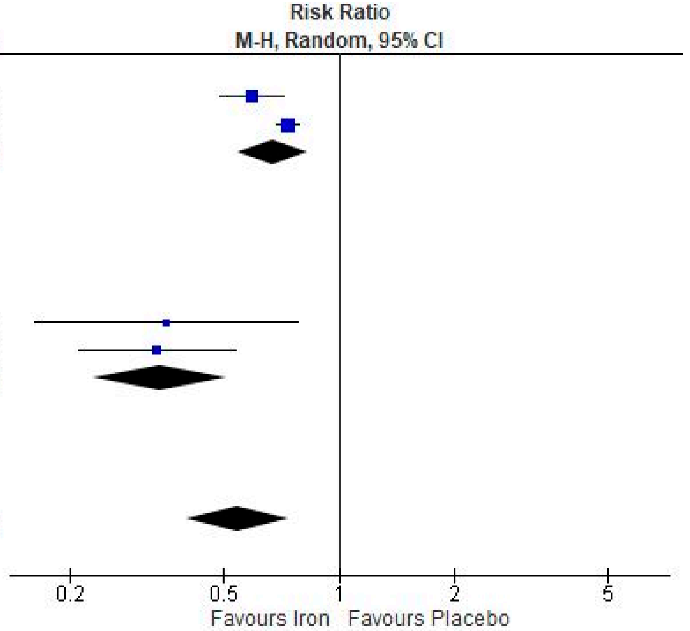

Footnotes

(1) Defined as serum ferritin <12ug/L

(2) Defined as serum ferritin <15ug/L

(3) Defined as serum ferritin <12 ug/L

(4) Defined as serum ferritin <12ug/L

Neonatal mortality

Study or Subgroup	Iron Events	Iron Total	Placebo Events	Placebo Total	Weight	Risk Ratio M-H, Random, 95% CI
Christian 2003	24	777	24	772	32.8%	0.99 [0.57, 1.73]
Liu 2013	32	5926	29	5906	36.5%	1.10 [0.67, 1.82]
Zeng 2008	16	1154	33	1259	30.7%	0.53 [0.29, 0.96]
**Total (95% CI)**		**7857**		**7937**	**100.0%**	**0.85 [0.55, 1.31]**
Total events	72		86			
Heterogeneity: Tau² = 0.07; Chi² = 3.78, df = 2 (P = 0.15); I² = 47%						
Test for overall effect: Z = 0.73 (P = 0.46)						

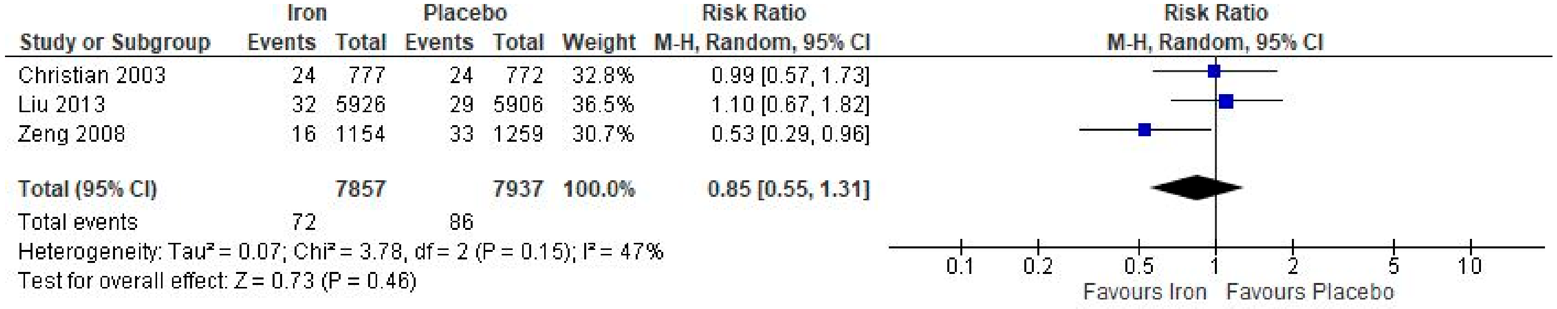

Supplementary Materials

Infant mortality

Study or Subgroup	Iron Events	Iron Total	Placebo Events	Placebo Total	Weight	Risk Ratio M-H, Random, 95% CI
Christian 2003	40	772	34	777	38.0%	1.18 [0.76, 1.85]
Etheredge 2015	20	675	16	692	18.0%	1.28 [0.67, 2.45]
Liu 2013	44	5926	45	5906	44.1%	0.97 [0.64, 1.47]
**Total (95% CI)**		**7373**		**7375**	**100.0%**	**1.10 [0.84, 1.45]**
Total events	104		95			

Heterogeneity: Tau² = 0.00; Chi² = 0.65, df = 2 (P = 0.72); I² = 0%

Test for overall effect: Z = 0.69 (P = 0.49)

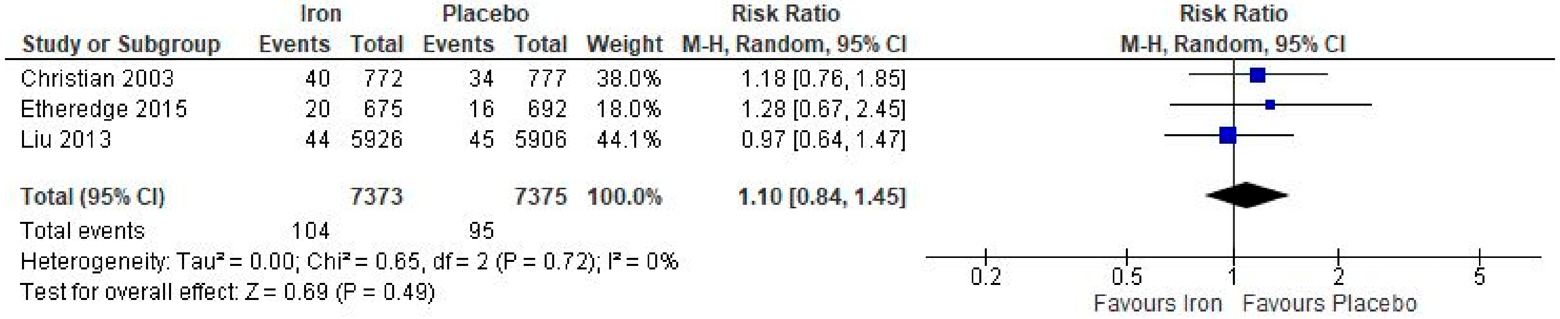

Preterm births

Study or Subgroup	Iron Events	Iron Total	Placebo Events	Placebo Total	Weight	Risk Ratio M-H, Random, 95% CI
Christian 2003	633	635	628	628	19.7%	1.00 [0.99, 1.00]
Etheredge 2015	100	665	113	685	18.4%	0.91 [0.71, 1.17]
Liu 2013	340	5926	353	5906	19.3%	0.96 [0.83, 1.11]
Ouladsahebmadarek 2011	16	410	18	372	12.9%	0.81 [0.42, 1.56]
Zeng 2008	59	1183	79	1282	17.5%	0.81 [0.58, 1.12]
Ziaei 2007	17	370	13	357	12.3%	1.26 [0.62, 2.56]
**Total (95% CI)**		**9189**		**9230**	**100.0%**	**0.94 [0.63, 1.41]**
Total events	1165		1204			

Heterogeneity: Tau² = 0.21; Chi² = 132.28, df = 5 (P < 0.00001); I² = 96%

Test for overall effect: Z = 0.30 (P = 0.76)

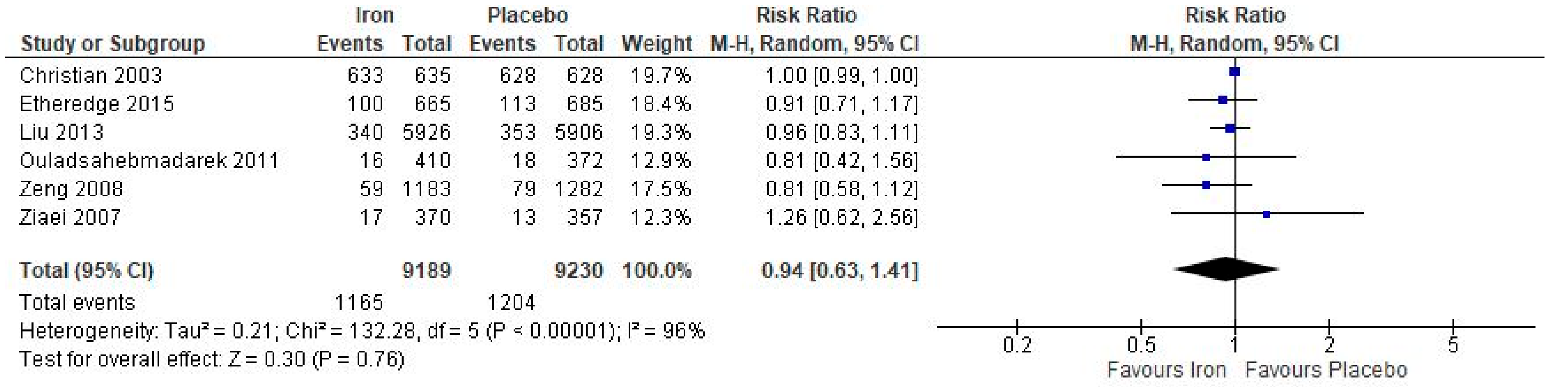

Supplementary Materials

Small-for-gestational-age babies

Study or Subgroup	Iron Events	Iron Total	Placebo Events	Placebo Total	Weight	Risk Ratio M-H, Random, 95% CI
Christian 2003	328	635	363	628	34.6%	0.89 [0.81, 0.99]
Etheredge 2015	101	641	105	648	21.8%	0.97 [0.76, 1.25]
Zeng 2008	278	1565	280	1705	30.3%	1.08 [0.93, 1.26]
Ziaei 2007	57	370	36	357	13.3%	1.53 [1.03, 2.26]
**Total (95% CI)**		**3211**		**3338**	**100.0%**	**1.04 [0.87, 1.24]**
Total events	764		784			

Heterogeneity: Tau² = 0.02; Chi² = 10.37, df = 3 (P = 0.02); I² = 71%
Test for overall effect: Z = 0.39 (P = 0.69)

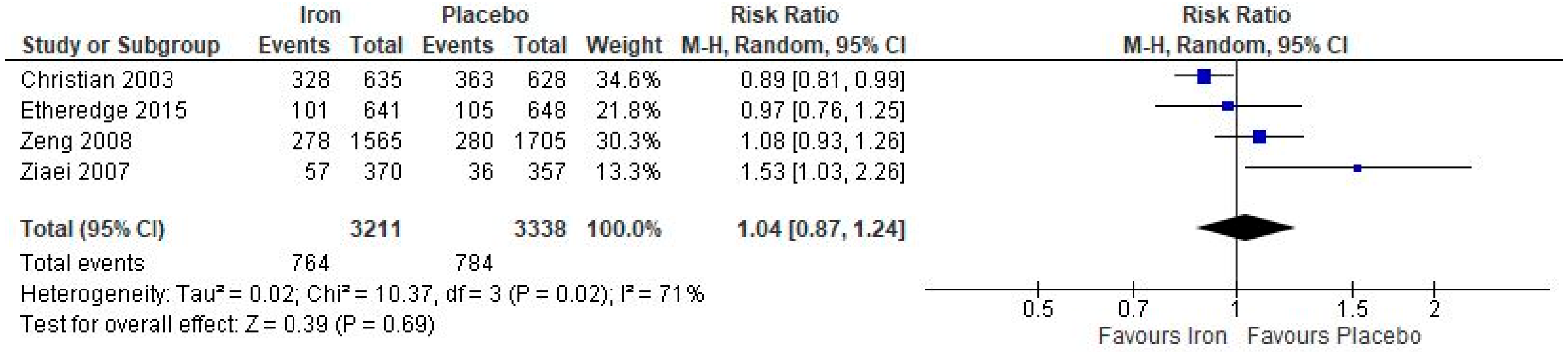

Perinatal mortality

Study or Subgroup	Iron Events	Iron Total	Placebo Events	Placebo Total	Weight	Risk Ratio M-H, Random, 95% CI
Christian 2003	50	814	56	801	31.2%	0.88 [0.61, 1.27]
Liu 2013	52	5954	52	5934	29.0%	1.00 [0.68, 1.46]
Zeng 2008	57	1546	76	1688	37.5%	0.82 [0.58, 1.15]
Ziaei 2007	3	370	6	357	2.2%	0.48 [0.12, 1.91]
**Total (95% CI)**		**8684**		**8780**	**100.0%**	**0.88 [0.71, 1.08]**
Total events	162		190			

Heterogeneity: Tau² = 0.00; Chi² = 1.31, df = 3 (P = 0.73); I² = 0%
Test for overall effect: Z = 1.26 (P = 0.21)

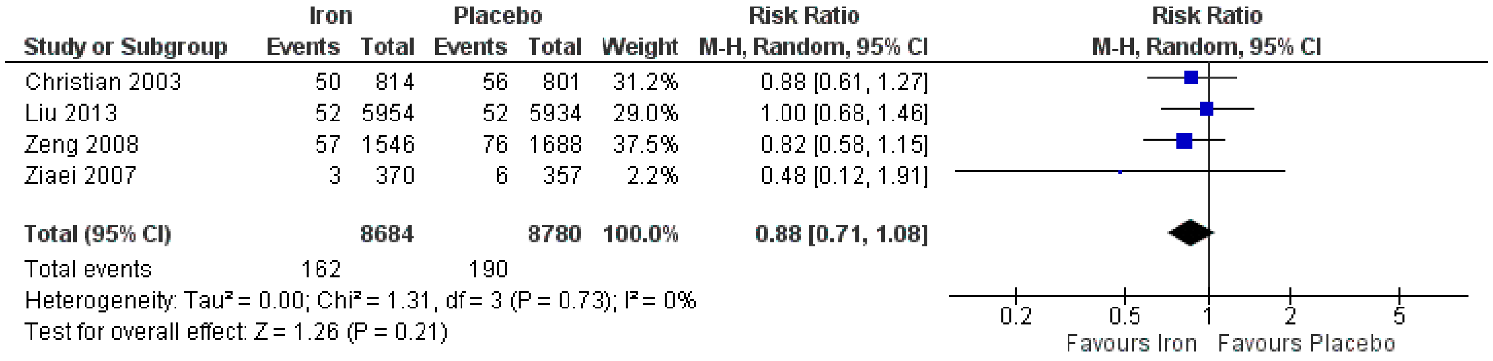

Pre-eclampsia/eclampsia

Study or Subgroup	Iron Events	Iron Total	Placebo Events	Placebo Total	Weight	Risk Ratio M-H, Random, 95% CI
Christian 2003	11	639	9	625	36.7%	1.20 [0.50, 2.86]
Ouladsahebmadarek 2011	16	410	10	372	46.3%	1.45 [0.67, 3.16]
Ziaei 2007	10	370	3	357	17.0%	3.22 [0.89, 11.59]
**Total (95% CI)**		**1419**		**1354**	**100.0%**	**1.55 [0.91, 2.63]**
Total events	37		22			

Heterogeneity: Tau² = 0.00; Chi² = 1.62, df = 2 (P = 0.44); I² = 0%
Test for overall effect: Z = 1.62 (P = 0.11)

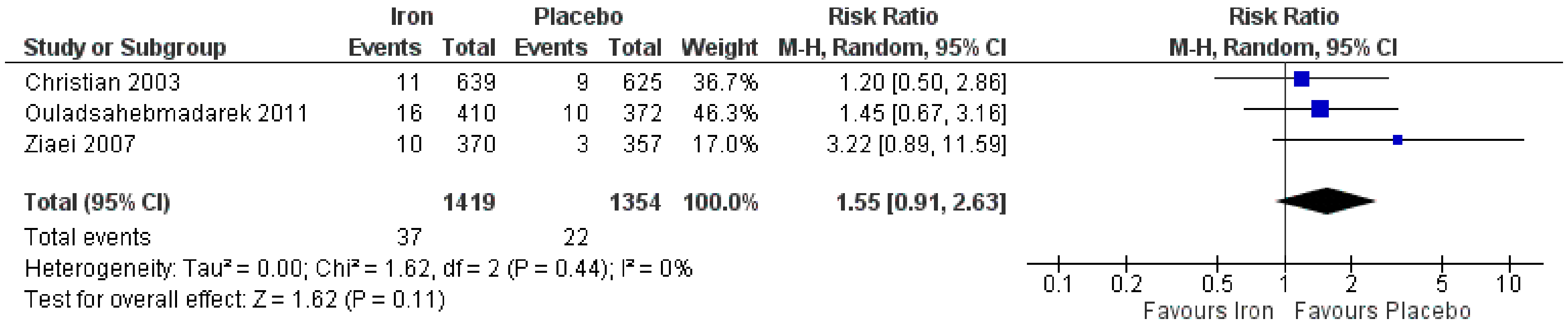

Supplementary Materials

Maternal transferrin receptor concentration (mg/L)

Study or Subgroup	Iron Mean	Iron SD	Iron Total	Placebo Mean	Placebo SD	Placebo Total	Weight	Mean Difference IV, Random, 95% CI
Christian 2003	2.08	0.6	126	1.51	0.39	129	36.2%	0.57 [0.45, 0.69]
Liu 2013	3.99	4.6068	278	4.79	4.6921	282	27.4%	-0.80 [-1.57, -0.03]
Zhao 2015	2.02	0.7268	814	2.42	0.8656	802	36.4%	-0.40 [-0.48, -0.32]
**Total (95% CI)**			**1218**			**1213**	**100.0%**	**-0.16 [-0.96, 0.65]**

Heterogeneity: Tau² = 0.46; Chi² = 170.35, df = 2 (P < 0.00001); I² = 99%

Test for overall effect: Z = 0.39 (P = 0.70)

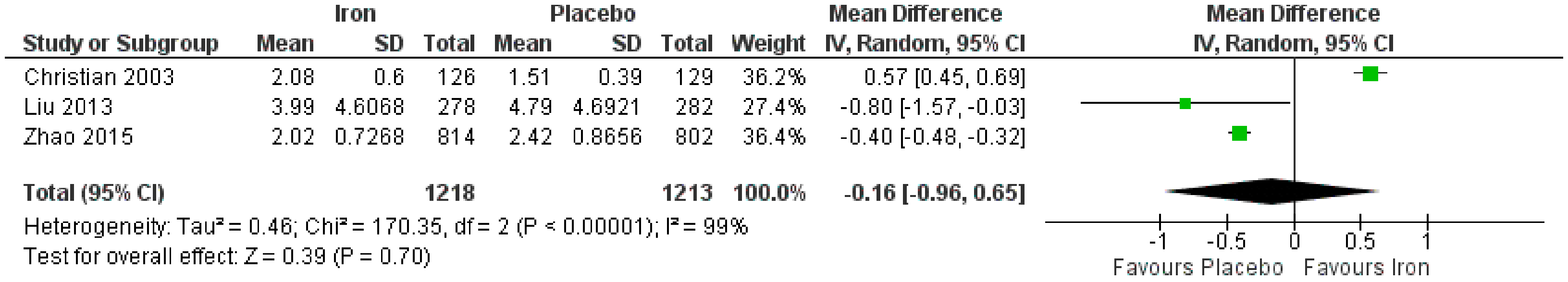

Subgroup Analysis: Maternal Anemia (geographical region)

Study or Subgroup	Iron Events	Iron Total	Placebo Events	Placebo Total	Weight	Risk Ratio M-H, Random, 95% CI
**11.1.1 Africa**						
Etheredge 2015	144	481	248	501	21.1%	0.60 [0.51, 0.71]
Preziosi 1997	41	99	69	98	17.8%	0.59 [0.45, 0.77]
**Subtotal (95% CI)**		**580**		**599**	**38.9%**	**0.60 [0.52, 0.69]**
Total events	185		317			
Heterogeneity: Tau² = 0.00; Chi² = 0.03, df = 1 (P = 0.86); I² = 0%						
Test for overall effect: Z = 7.20 (P < 0.00001)						
**11.1.2 Western Pacific**						
Liu 2013	327	5913	452	5896	21.8%	0.72 [0.63, 0.83]
Zeng 2008	87	278	133	218	19.9%	0.51 [0.42, 0.63]
Zhao 2015	109	1179	201	800	19.5%	0.37 [0.30, 0.46]
**Subtotal (95% CI)**		**7370**		**6914**	**61.1%**	**0.52 [0.35, 0.77]**
Total events	523		786			
Heterogeneity: Tau² = 0.12; Chi² = 28.30, df = 2 (P < 0.00001); I² = 93%						
Test for overall effect: Z = 3.23 (P = 0.001)						
**Total (95% CI)**		**7950**		**7513**	**100.0%**	**0.55 [0.44, 0.69]**
Total events	708		1103			

Heterogeneity: Tau² = 0.06; Chi² = 28.55, df = 4 (P < 0.00001); I² = 86%

Test for overall effect: Z = 5.20 (P < 0.00001)

Test for subgroup differences: Chi² = 0.47, df = 1 (P = 0.49), I² = 0%

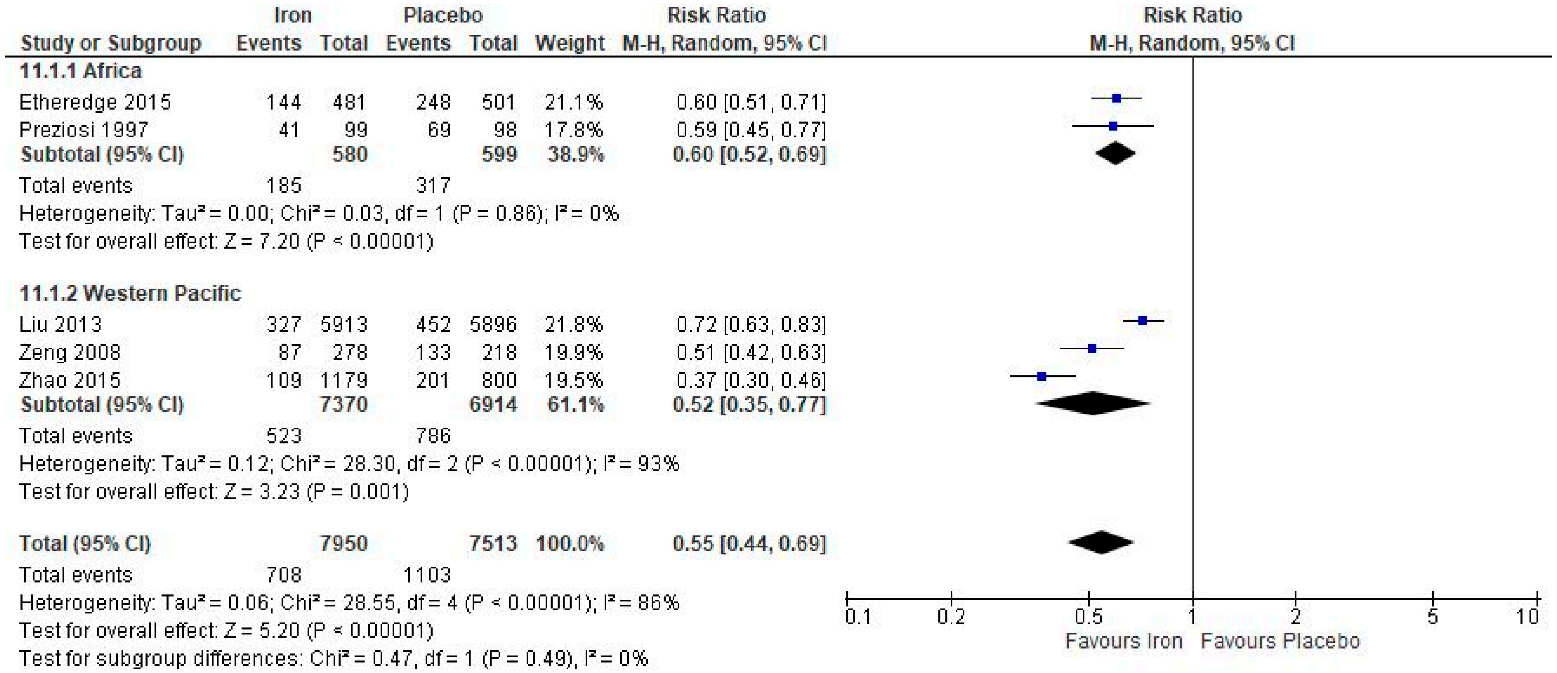

Supplementary Materials

Subgroup Analysis: Maternal anemia (dose of iron, mg)

Study or Subgroup	Iron Events	Iron Total	Placebo Events	Placebo Total	Weight	Risk Ratio M-H, Random, 95% CI
**11.2.1 > or equal to 60 mg of Fe**						
Etheredge 2015	144	481	248	501	18.1%	0.60 [0.51, 0.71]
Preziosi 1997	41	99	69	98	15.2%	0.59 [0.45, 0.77]
Zeng 2008	87	278	133	218	17.0%	0.51 [0.42, 0.63]
Zhao 2015	109	1179	201	800	16.7%	0.37 [0.30, 0.46]
**Subtotal (95% CI)**		**2037**		**1617**	**67.0%**	**0.51 [0.41, 0.64]**
Total events	381		651			
Heterogeneity: Tau² = 0.04; Chi² = 14.40, df = 3 (P = 0.002); I² = 79%						
Test for overall effect: Z = 5.80 (P < 0.00001)						
**11.2.2 < 60 mg of Fe**						
Christian 2003	38	136	89	138	14.4%	0.43 [0.32, 0.58]
Liu 2013	327	5913	452	5896	18.7%	0.72 [0.63, 0.83]
**Subtotal (95% CI)**		**6049**		**6034**	**33.0%**	**0.57 [0.34, 0.94]**
Total events	365		541			
Heterogeneity: Tau² = 0.12; Chi² = 9.45, df = 1 (P = 0.002); I² = 89%						
Test for overall effect: Z = 2.20 (P = 0.03)						
**Total (95% CI)**		**8086**		**7651**	**100.0%**	**0.53 [0.43, 0.65]**
Total events	746		1192			
Heterogeneity: Tau² = 0.06; Chi² = 32.11, df = 5 (P < 0.00001); I² = 84%						
Test for overall effect: Z = 5.96 (P < 0.00001)						
Test for subgroup differences: Chi² = 0.15, df = 1 (P = 0.69), I² = 0%						

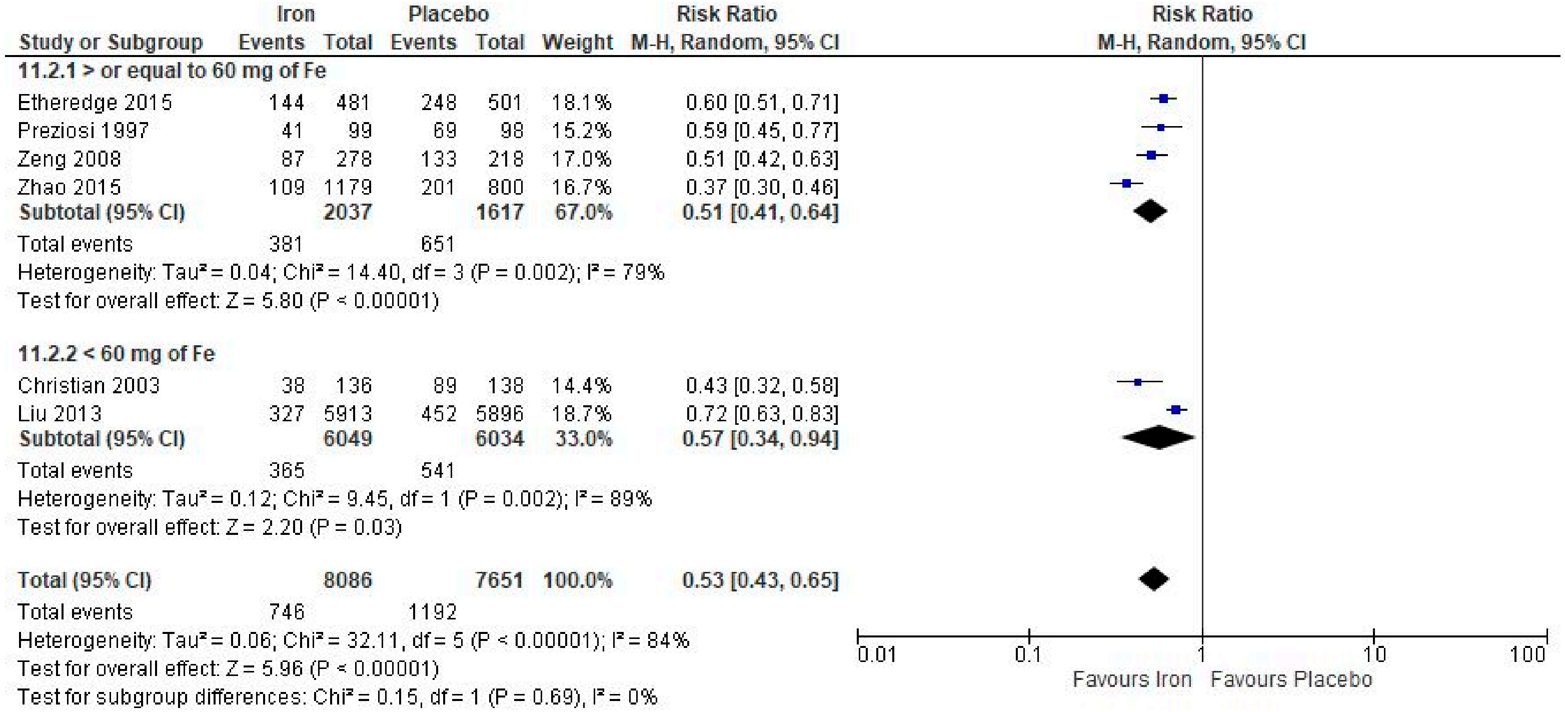

Supplementary Materials

<u>Comparison: Vitamin D Supplementation vs. Placebo/No Intervention</u>

Small-for-gestational-age infants

Study or Subgroup	Vitamin D Events	Vitamin D Total	Placebo Events	Placebo Total	Weight	Risk Ratio M-H, Random, 95% CI
**7.2.1 Vit D vs. Placebo (additional micronutrients)**						
Hossain 2012	19	86	18	89	31.4%	1.09 [0.62, 1.94]
Roth 2018 (MDIG)	84	252	72	259	46.7%	1.20 [0.92, 1.56]
**Subtotal (95% CI)**		**338**		**348**	**78.1%**	**1.18 [0.93, 1.50]**
Total events	103		90			
Heterogeneity: Tau² = 0.00; Chi² = 0.08, df = 1 (P = 0.77); I² = 0%						
Test for overall effect: Z = 1.36 (P = 0.17)						
**7.2.2 Strictly Vit D vs. Placebo**						
Sablok 2015	9	108	11	57	21.9%	0.43 [0.19, 0.98]
**Subtotal (95% CI)**		**108**		**57**	**21.9%**	**0.43 [0.19, 0.98]**
Total events	9		11			
Heterogeneity: Not applicable						
Test for overall effect: Z = 2.01 (P = 0.04)						
**Total (95% CI)**		**446**		**405**	**100.0%**	**0.93 [0.57, 1.53]**
Total events	112		101			
Heterogeneity: Tau² = 0.12; Chi² = 5.42, df = 2 (P = 0.07); I² = 63%						
Test for overall effect: Z = 0.28 (P = 0.78)						
Test for subgroup differences: Chi² = 5.32, df = 1 (P = 0.02), I² = 81.2%						

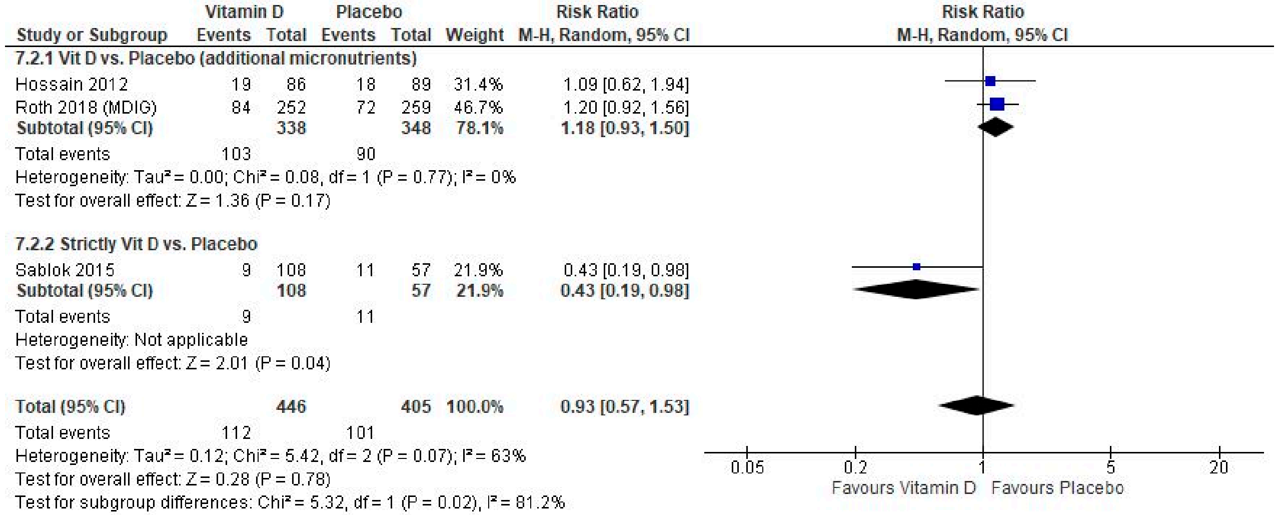

Caesarean section as a mode of delivery

Study or Subgroup	Vitamin D Events	Vitamin D Total	Placebo Events	Placebo Total	Weight	Risk Ratio M-H, Random, 95% CI
**7.5.1 Vit D vs. Placebo (additional micronutrients)**						
Charandabi 2015	30	42	26	42	13.7%	1.15 [0.85, 1.56]
Hossain 2012	49	86	46	89	17.2%	1.10 [0.84, 1.45]
Roth 2013 (AViDD)	44	73	44	74	18.1%	1.01 [0.78, 1.32]
Roth 2018 (MDIG)	127	260	121	259	39.3%	1.05 [0.87, 1.25]
**Subtotal (95% CI)**		**461**		**464**	**88.3%**	**1.07 [0.95, 1.20]**
Total events	250		237			
Heterogeneity: Tau² = 0.00; Chi² = 0.50, df = 3 (P = 0.92); I² = 0%						
Test for overall effect: Z = 1.04 (P = 0.30)						
**7.5.2 Strictly Vit D vs. Placebo**						
Naghshineh 2016	34	68	36	70	11.7%	0.97 [0.70, 1.35]
**Subtotal (95% CI)**		**68**		**70**	**11.7%**	**0.97 [0.70, 1.35]**
Total events	34		36			
Heterogeneity: Not applicable						
Test for overall effect: Z = 0.17 (P = 0.87)						
**Total (95% CI)**		**529**		**534**	**100.0%**	**1.05 [0.94, 1.18]**
Total events	284		273			
Heterogeneity: Tau² = 0.00; Chi² = 0.77, df = 4 (P = 0.94); I² = 0%						
Test for overall effect: Z = 0.92 (P = 0.36)						
Test for subgroup differences: Chi² = 0.26, df = 1 (P = 0.61), I² = 0%						

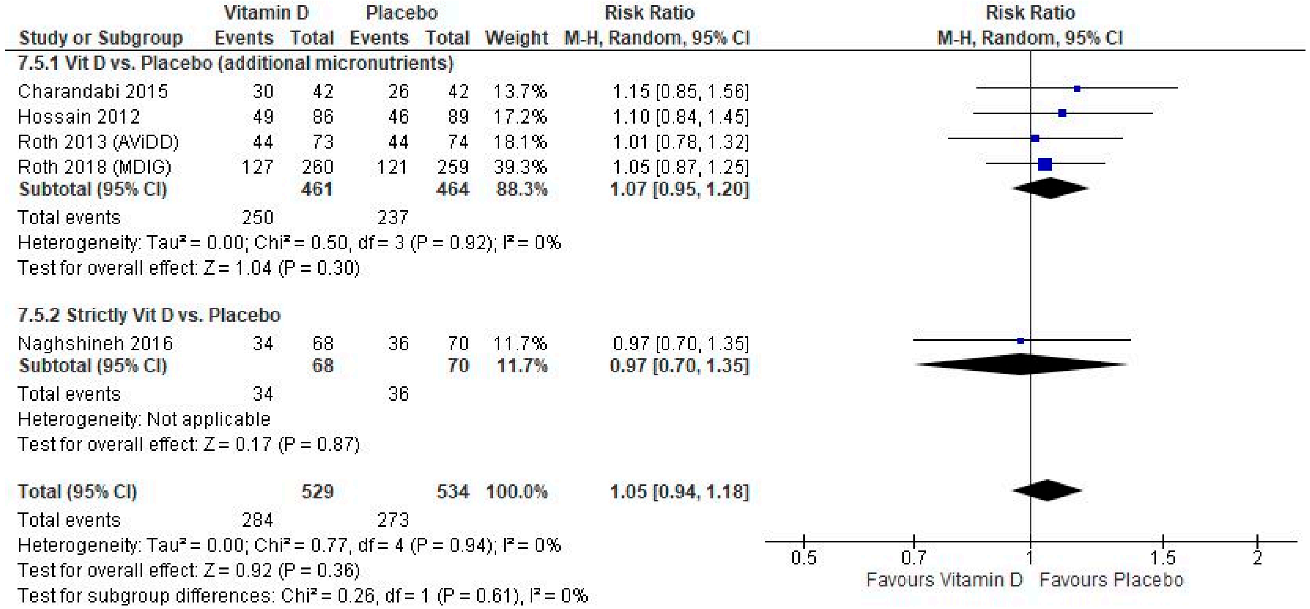

Supplementary Materials

Maternal serum/plasma calcium concentration (mg/dL)

Study or Subgroup	Vitamin D Mean	SD	Total	Placebo Mean	SD	Total	Weight	Mean Difference IV, Random, 95% CI
Asemi 2013	8.88	0.13	24	9.05	0.07	24	28.0%	-0.17 [-0.23, -0.11]
Roth 2013 (AViDD)	9.3	0.4	67	9.26	0.44	63	23.0%	0.04 [-0.10, 0.18]
Roth 2018 (MDIG)	9.14	0.48	199	9.06	0.44	206	26.5%	0.08 [-0.01, 0.17]
Sahu 2009	9.16	0.84	35	9.24	0.72	14	7.6%	-0.08 [-0.55, 0.39]
Vaziri 2016	8.11	0.8	62	8.33	0.77	65	14.9%	-0.22 [-0.49, 0.05]
**Total (95% CI)**			**387**			**372**	**100.0%**	**-0.06 [-0.21, 0.09]**

Heterogeneity: Tau² = 0.02; Chi² = 24.73, df = 4 (P < 0.0001); I² = 84%
Test for overall effect: Z = 0.73 (P = 0.47)

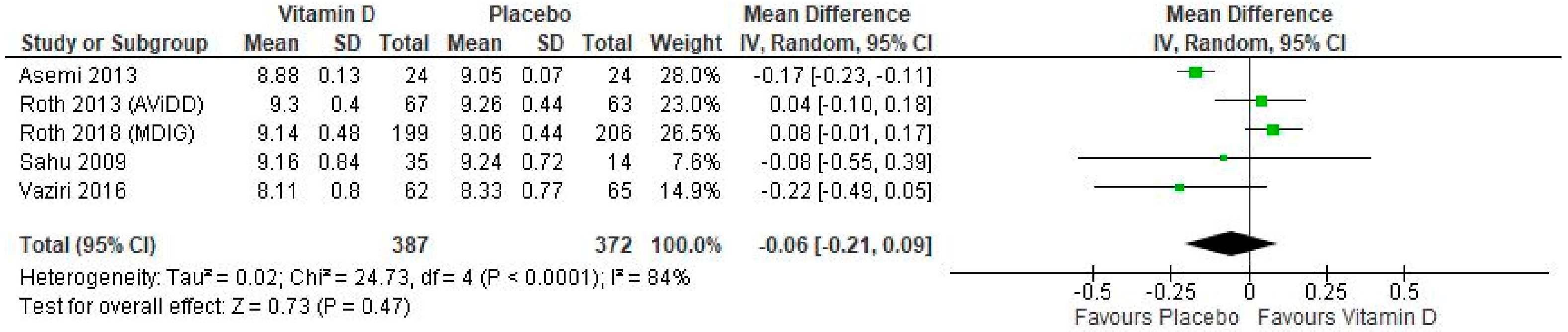

Maternal serum/plasma vitamin D concentration (nmol/L)

Study or Subgroup	Vitamin D Mean	SD	Total	Placebo Mean	SD	Total	Weight	Mean Difference IV, Random, 95% CI
**7.3.1 Vit D vs. Placebo (additional micronutrients)**								
Asemi 2013	53.66	4.49	24	33.2	2.75	24	11.7%	20.46 [18.35, 22.57]
Hossain 2012	45.68	27.46	86	17.22	17.47	89	11.6%	28.46 [21.61, 35.31]
Roth 2013 (AViDD)	134.4	30.7	80	38.4	18.1	80	11.6%	96.00 [88.19, 103.81]
Roth 2018 (MDIG)	110.7	28	118	23.7	13.9	128	11.6%	87.00 [81.40, 92.60]
Sahu 2009	60.87	70.63	35	24.53	14.69	14	10.3%	36.34 [11.71, 60.97]
Vaziri 2016	45.03	23.94	62	30	14.53	65	11.6%	15.03 [8.10, 21.96]
**Subtotal (95% CI)**			**405**			**400**	**68.4%**	**47.36 [17.95, 76.76]**
Heterogeneity: Tau² = 1317.92; Chi² = 767.92, df = 5 (P < 0.00001); I² = 99%								
Test for overall effect: Z = 3.16 (P = 0.002)								
**7.3.2 Strictly Vit D vs. Placebo**								
Khan 2016	38.34	18.99	32	28.35	11.76	40	11.6%	9.99 [2.47, 17.51]
Sabet 2012	153.38	74.88	25	73.38	39.94	25	9.4%	80.00 [46.73, 113.27]
Sablok 2015	80.2	51.53	108	46.11	74.21	57	10.6%	34.09 [12.51, 55.67]
**Subtotal (95% CI)**			**165**			**122**	**31.6%**	**38.12 [2.94, 73.30]**
Heterogeneity: Tau² = 837.86; Chi² = 19.35, df = 2 (P < 0.0001); I² = 90%								
Test for overall effect: Z = 2.12 (P = 0.03)								
**Total (95% CI)**			**570**			**522**	**100.0%**	**44.70 [21.94, 67.45]**

Heterogeneity: Tau² = 1149.40; Chi² = 805.88, df = 8 (P < 0.00001); I² = 99%
Test for overall effect: Z = 3.85 (P = 0.0001)
Test for subgroup differences: Chi² = 0.16, df = 1 (P = 0.69), I² = 0%

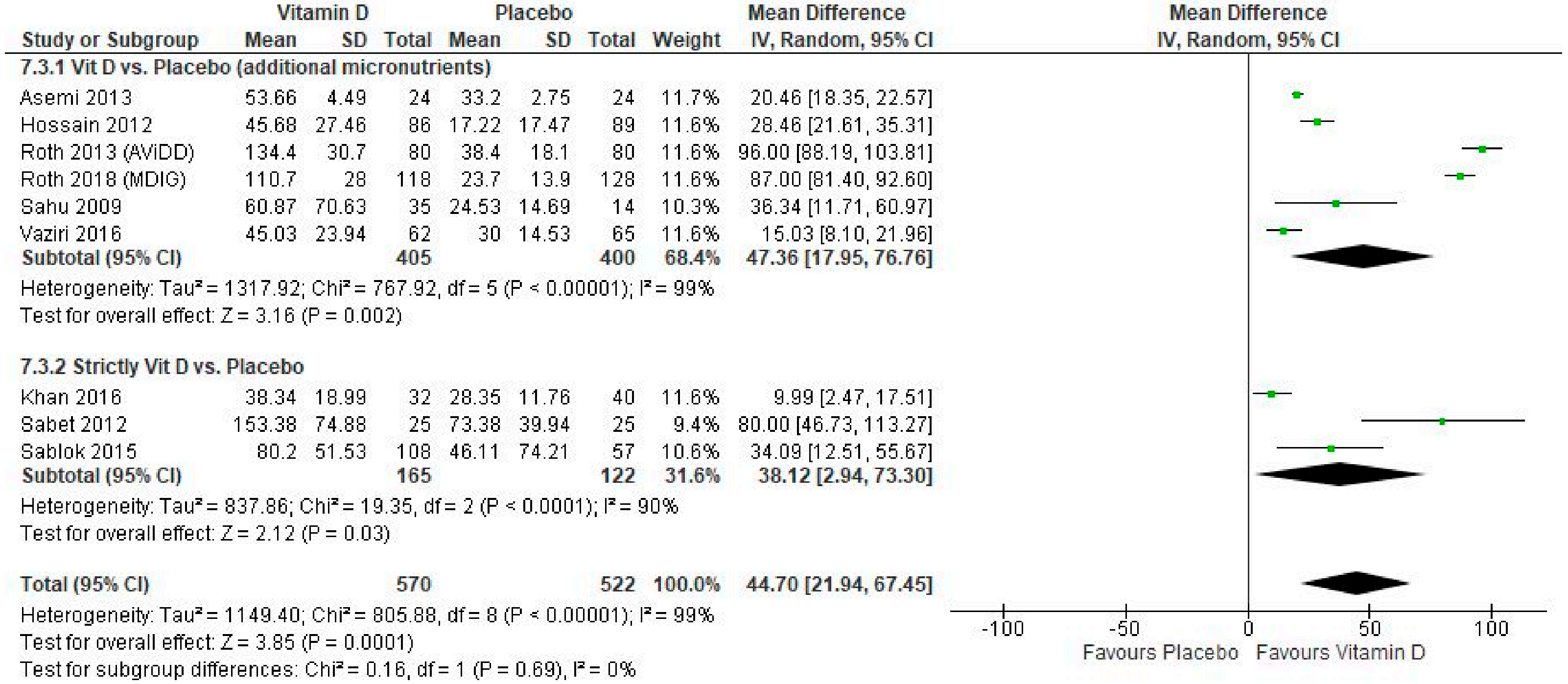

Supplementary Materials

<u>Comparison: Calcium Supplementation vs. Placebo/No Intervention</u>

Low birthweight

Study or Subgroup	Calcium Events	Calcium Total	Placebo Events	Placebo Total	Weight	Risk Ratio M-H, Random, 95% CI
**8.1.1 Calcium vs. Placebo (additional micronutrients)**						
Villar 2006	512	3930	524	3938	16.0%	0.98 [0.87, 1.10]
**Subtotal (95% CI)**		**3930**		**3938**	**16.0%**	**0.98 [0.87, 1.10]**
Total events	512		524			
Heterogeneity: Not applicable						
Test for overall effect: Z = 0.36 (P = 0.72)						
**8.1.2 Strictly Calcium vs. Placebo**						
Belizán 1997	31	547	41	559	1.0%	0.77 [0.49, 1.21]
Kumar 2009	251	273	232	251	83.0%	0.99 [0.95, 1.05]
**Subtotal (95% CI)**		**820**		**810**	**84.0%**	**0.92 [0.66, 1.29]**
Total events	282		273			
Heterogeneity: Tau² = 0.04; Chi² = 2.55, df = 1 (P = 0.11); I² = 61%						
Test for overall effect: Z = 0.49 (P = 0.63)						
**Total (95% CI)**		**4750**		**4748**	**100.0%**	**0.99 [0.95, 1.04]**
Total events	794		797			
Heterogeneity: Tau² = 0.00; Chi² = 1.74, df = 2 (P = 0.42); I² = 0%						
Test for overall effect: Z = 0.45 (P = 0.65)						
Test for subgroup differences: Chi² = 0.12, df = 1 (P = 0.73), I² = 0%						

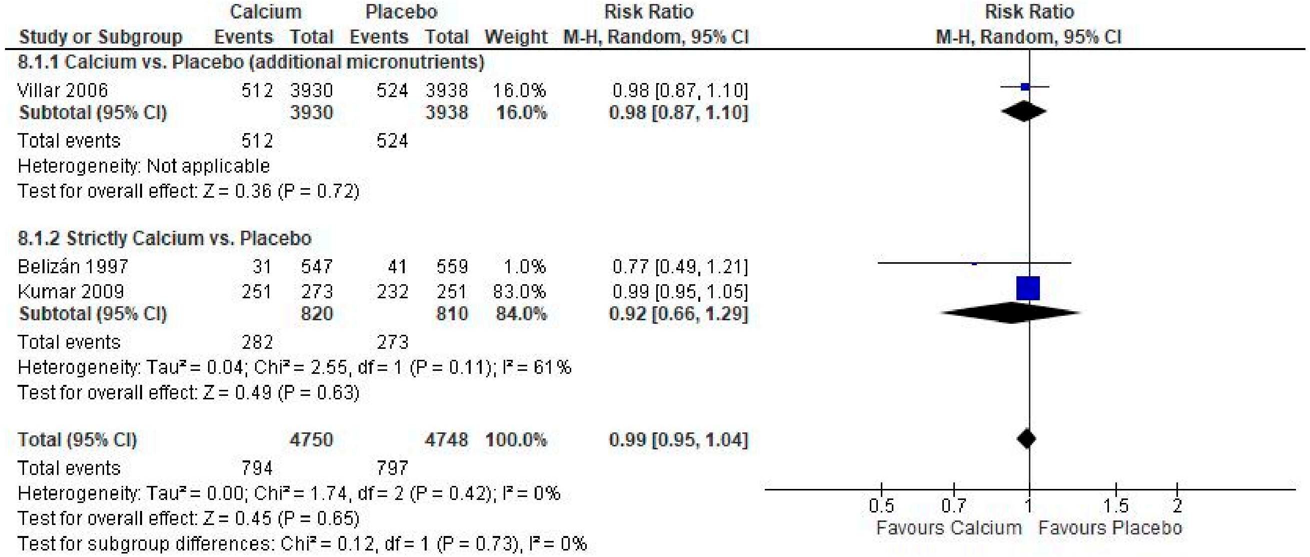

Stillbirths

Study or Subgroup	Calcium Events	Calcium Total	Placebo Events	Placebo Total	Weight	Risk Ratio M-H, Random, 95% CI
**8.3.1 Calcium vs. Placebo (additional micronutrients)**						
Villar 2006	142	4181	166	4197	92.9%	0.86 [0.69, 1.07]
**Subtotal (95% CI)**		**4181**		**4197**	**92.9%**	**0.86 [0.69, 1.07]**
Total events	142		166			
Heterogeneity: Not applicable						
Test for overall effect: Z = 1.36 (P = 0.17)						
**8.3.2 Strictly Calcium vs. Placebo**						
Belizán 1997	6	558	7	567	3.8%	0.87 [0.29, 2.58]
Kumar 2009	6	273	5	251	3.3%	1.10 [0.34, 3.57]
López-Jaramillo 1997	0	125	0	135		Not estimable
**Subtotal (95% CI)**		**956**		**953**	**7.1%**	**0.97 [0.44, 2.15]**
Total events	12		12			
Heterogeneity: Tau² = 0.00; Chi² = 0.08, df = 1 (P = 0.77); I² = 0%						
Test for overall effect: Z = 0.07 (P = 0.94)						
**Total (95% CI)**		**5137**		**5150**	**100.0%**	**0.87 [0.70, 1.07]**
Total events	154		178			
Heterogeneity: Tau² = 0.00; Chi² = 0.17, df = 2 (P = 0.92); I² = 0%						
Test for overall effect: Z = 1.33 (P = 0.18)						
Test for subgroup differences: Chi² = 0.09, df = 1 (P = 0.77), I² = 0%						

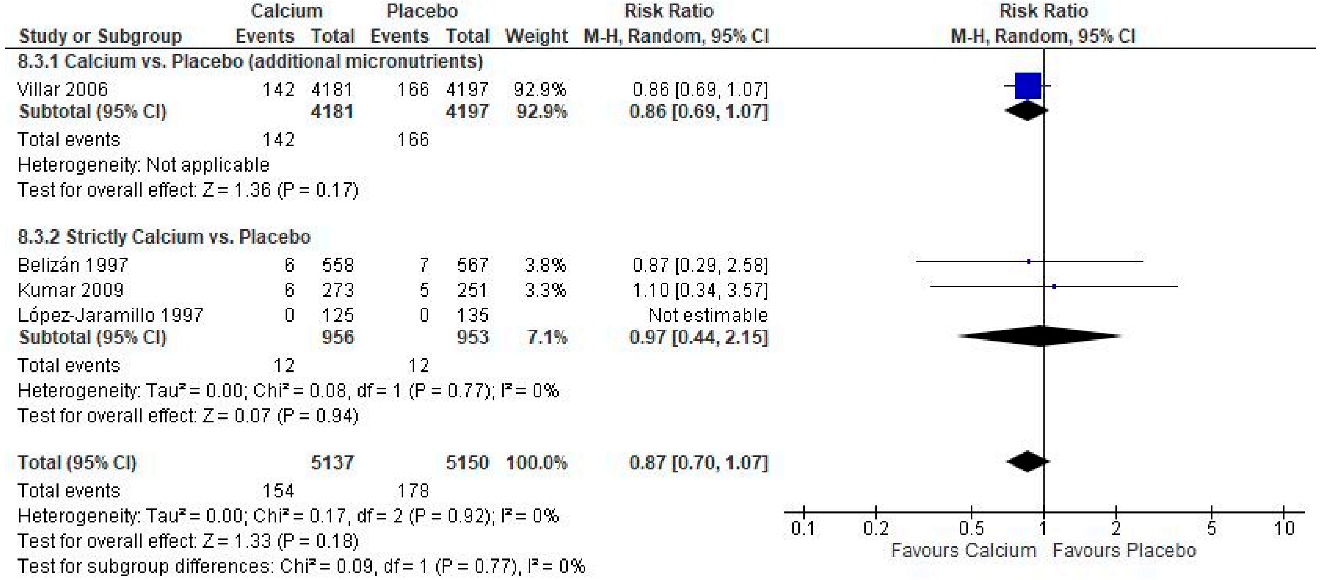

Supplementary Materials

Preterm births

Study or Subgroup	Calcium Events	Calcium Total	Placebo Events	Placebo Total	Weight	Risk Ratio M-H, Random, 95% CI
**8.4.1 Calcium vs. Placebo (additional micronutrients)**						
Villar 2006	398	4038	436	4042	61.5%	0.91 [0.80, 1.04]
**Subtotal (95% CI)**		**4038**		**4042**	**61.5%**	**0.91 [0.80, 1.04]**
Total events	398		436			
Heterogeneity: Not applicable						
Test for overall effect: Z = 1.37 (P = 0.17)						
**8.4.2 Strictly Calcium vs. Placebo**						
Belizán 1997	33	527	37	542	21.8%	0.92 [0.58, 1.44]
Kumar 2009	19	273	32	251	16.8%	0.55 [0.32, 0.94]
López-Jaramillo 1997	0	125	0	135		Not estimable
**Subtotal (95% CI)**		**925**		**928**	**38.5%**	**0.72 [0.44, 1.20]**
Total events	52		69			
Heterogeneity: Tau² = 0.07; Chi² = 2.07, df = 1 (P = 0.15); I² = 52%						
Test for overall effect: Z = 1.25 (P = 0.21)						
**Total (95% CI)**		**4963**		**4970**	**100.0%**	**0.84 [0.65, 1.08]**
Total events	450		505			
Heterogeneity: Tau² = 0.02; Chi² = 3.31, df = 2 (P = 0.19); I² = 40%						
Test for overall effect: Z = 1.36 (P = 0.17)						
Test for subgroup differences: Chi² = 0.77, df = 1 (P = 0.38), I² = 0%						

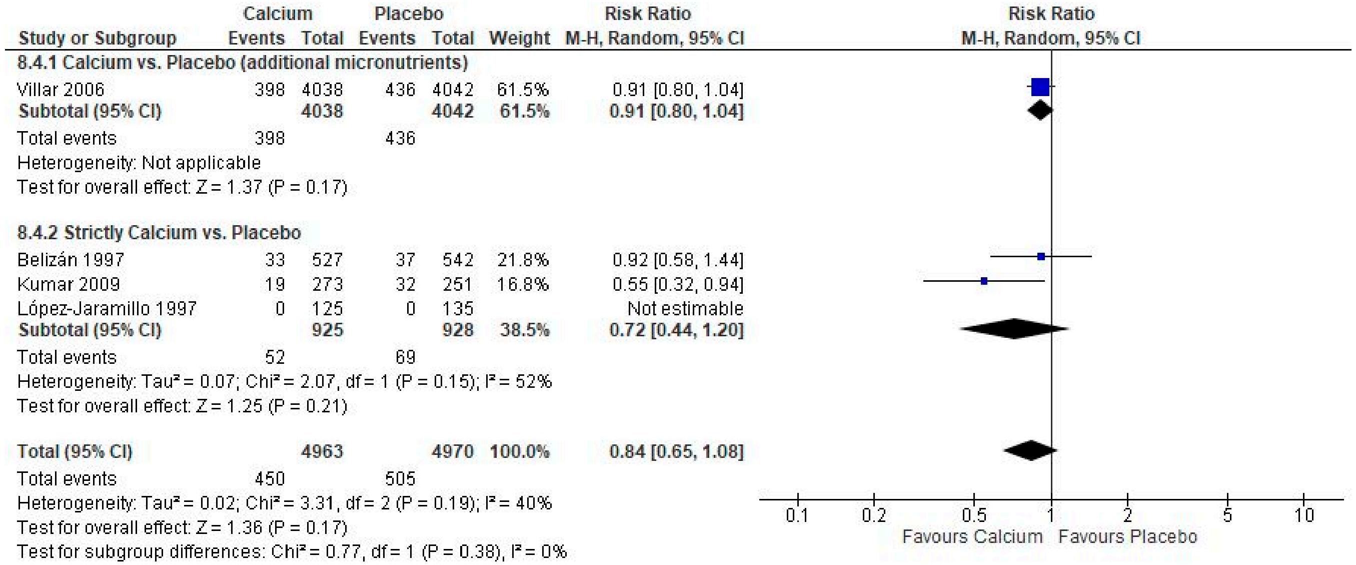

Caesarean section as a mode of delivery

Study or Subgroup	Calcium Events	Calcium Total	Placebo Events	Placebo Total	Weight	Risk Ratio M-H, Random, 95% CI
**8.5.1 Calcium vs. Placebo (additional micronutrients)**						
Villar 2006	809	4181	854	4197	71.4%	0.95 [0.87, 1.04]
**Subtotal (95% CI)**		**4181**		**4197**	**71.4%**	**0.95 [0.87, 1.04]**
Total events	809		854			
Heterogeneity: Not applicable						
Test for overall effect: Z = 1.15 (P = 0.25)						
**8.5.2 Strictly Calcium vs. Placebo**						
Belizán 1997	60	544	66	554	18.1%	0.93 [0.67, 1.29]
Kumar 2009	41	273	27	251	10.5%	1.40 [0.89, 2.20]
**Subtotal (95% CI)**		**817**		**805**	**28.6%**	**1.10 [0.74, 1.64]**
Total events	101		93			
Heterogeneity: Tau² = 0.04; Chi² = 2.06, df = 1 (P = 0.15); I² = 51%						
Test for overall effect: Z = 0.48 (P = 0.63)						
**Total (95% CI)**		**4998**		**5002**	**100.0%**	**0.99 [0.84, 1.15]**
Total events	910		947			
Heterogeneity: Tau² = 0.01; Chi² = 2.70, df = 2 (P = 0.26); I² = 26%						
Test for overall effect: Z = 0.19 (P = 0.85)						
Test for subgroup differences: Chi² = 0.50, df = 1 (P = 0.48), I² = 0%						

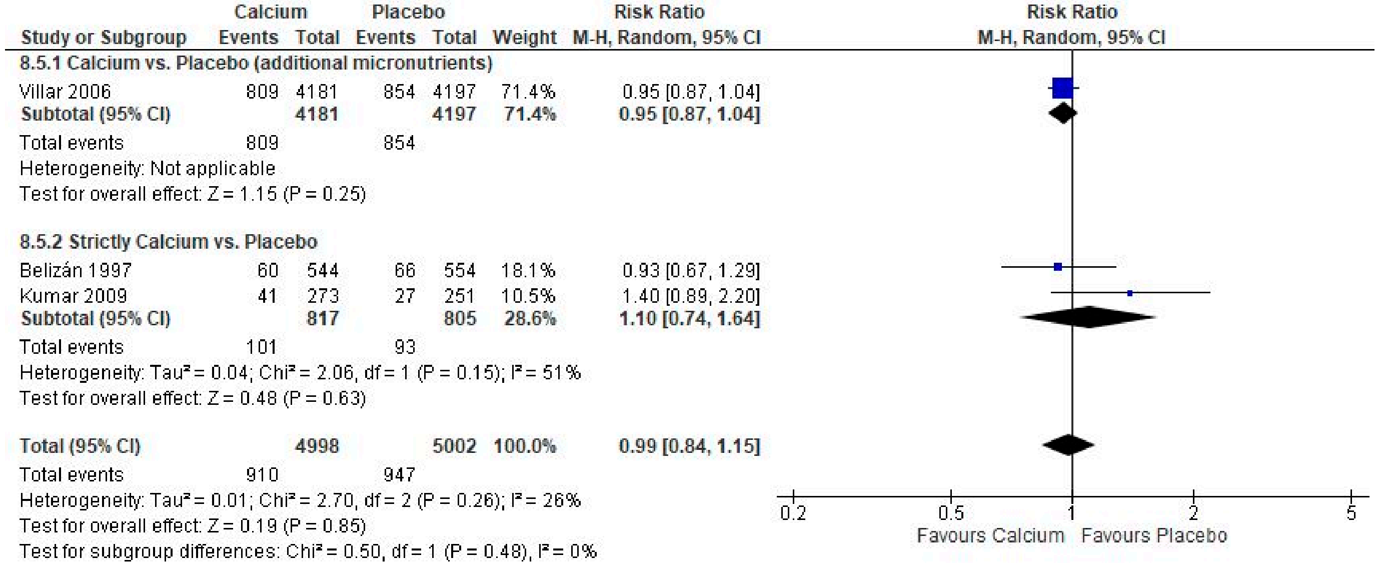

Supplementary Materials

Comparison: Iron-Folic Acid Supplementation vs. Folic-Acid Supplementation/Placebo

Perinatal mortality

	IFA		FA			Risk Ratio
**Study or Subgroup**	**Events**	**Total**	**Events**	**Total**	**Weight**	**M-H, Random, 95% CI**
Christian 2003 (1)	50	814	56	801	31.2%	0.88 [0.61, 1.27]
Liu 2013 (2)	52	5954	52	5934	29.0%	1.00 [0.68, 1.46]
Zeng 2008	57	1546	76	1688	37.5%	0.82 [0.58, 1.15]
Ziaei 2007	3	370	6	357	2.2%	0.48 [0.12, 1.91]
**Total (95% CI)**		**8684**		**8780**	**100.0%**	**0.88 [0.71, 1.08]**
Total events	162		190			

Heterogeneity: Tau² = 0.00; Chi² = 1.31, df = 3 (P = 0.73); I² = 0%
Test for overall effect: Z = 1.26 (P = 0.21)

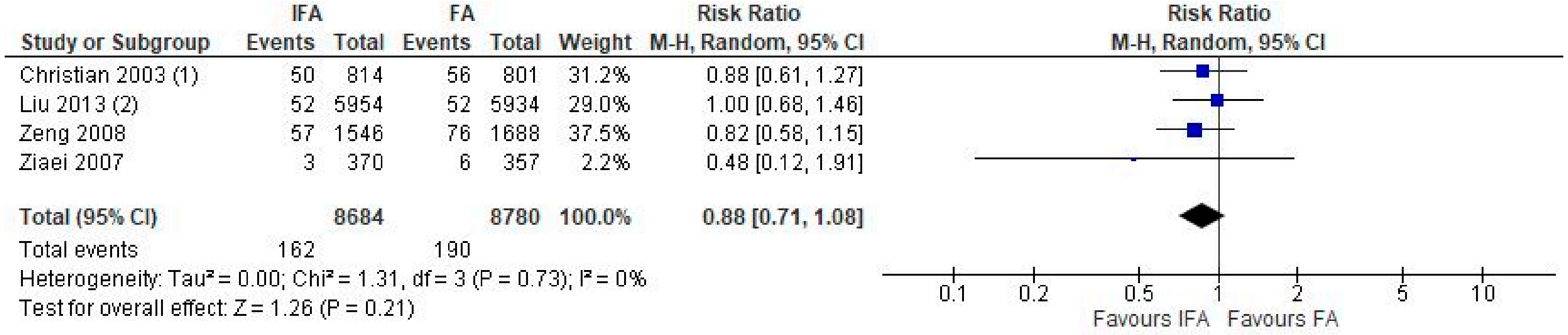

Footnotes
(1) Defined as: Stillbirth (from 28 weeks of gestation to delivery) and early neonatal death (from birth to 6 days after delivery)
(2) Defined as: Stillbirth (from 28 weeks of gestation to delivery) and early neonatal death (from birth to 6 days after delivery)

Maternal hemoglobin concentration (g/L)

	IFA			FA				Mean Difference
**Study or Subgroup**	**Mean**	**SD**	**Total**	**Mean**	**SD**	**Total**	**Weight**	**IV, Random, 95% CI**
Christian 2003	-1.6	20.3	149	-14.4	19.4	146	12.9%	12.80 [8.27, 17.33]
Etheredge 2015	1	22	473	-7	20	495	14.4%	8.00 [5.35, 10.65]
Liu 2013	122	9	5913	122	9	5896	15.2%	0.00 [-0.32, 0.32]
Menendez 1995	105.6298	19.7803	181	98.5795	17.186	176	13.5%	7.05 [3.21, 10.89]
Zeng 2008	110.1	14.8	149	105.3	14.2	168	14.0%	4.80 [1.60, 8.00]
Zhao 2015	122	14.6	814	117	7.2	802	15.1%	5.00 [3.88, 6.12]
Ziaei 2007	137.5	10.5	370	125.6	12.4	357	14.9%	11.90 [10.23, 13.57]
**Total (95% CI)**			**8049**			**8040**	**100.0%**	**6.95 [2.80, 11.11]**

Heterogeneity: Tau² = 29.46; Chi² = 316.25, df = 6 (P < 0.00001); I² = 98%
Test for overall effect: Z = 3.28 (P = 0.001)

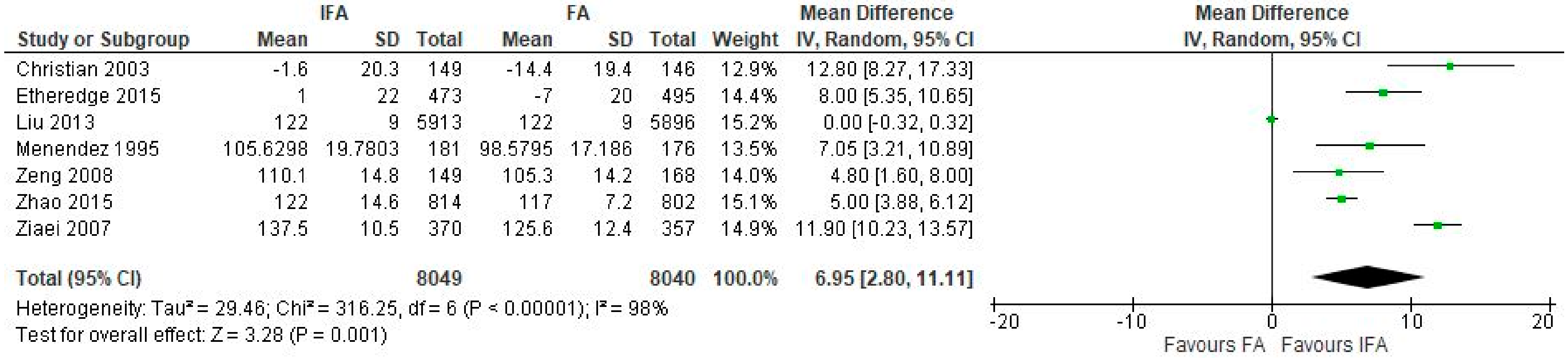

Supplementary Materials

Maternal serum/plasma ferritin concentration (ug/L)

Study or Subgroup	IFA Mean	IFA SD	IFA Total	FA Mean	FA SD	FA Total	Weight	Mean Difference IV, Random, 95% CI
Christian 2003	13.46	2.05	126	6.96	2.08	129	22.3%	6.50 [5.99, 7.01]
Etheredge 2015	41.3	295.6	483	11.3	92.1	503	11.1%	30.00 [2.44, 57.56]
Liu 2013	16.7	11.8577	278	11.3	7.6779	282	22.2%	5.40 [3.74, 7.06]
Menendez 1995	103.57	8.7721	237	63.179	5.8112	240	22.2%	40.39 [39.05, 41.73]
Zhao 2015	15.3	11.6281	814	11.1	7.2136	802	22.3%	4.20 [3.26, 5.14]
**Total (95% CI)**			**1938**			**1956**	**100.0%**	**15.87 [2.96, 28.79]**

Heterogeneity: Tau² = 194.91; Chi² = 2330.05, df = 4 (P < 0.00001); I² = 100%
Test for overall effect: Z = 2.41 (P = 0.02)

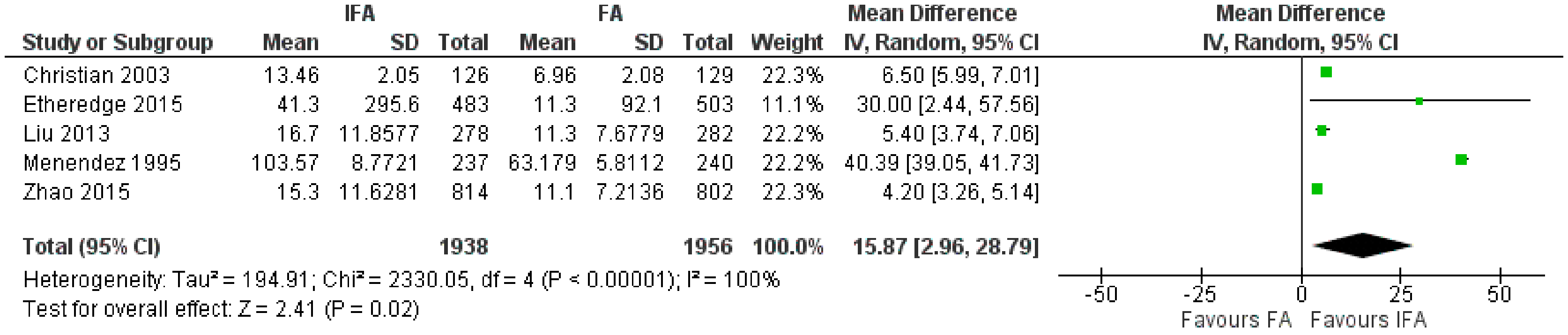

Maternal serum/plasma transferrin receptor concentration (mg/L)

Study or Subgroup	IFA Mean	IFA SD	IFA Total	FA Mean	FA SD	FA Total	Weight	Mean Difference IV, Random, 95% CI
Christian 2003	2.08	0.6	126	1.51	0.39	129	36.2%	0.57 [0.45, 0.69]
Liu 2013	3.99	4.6068	278	4.79	4.6921	282	27.4%	-0.80 [-1.57, -0.03]
Zhao 2015	2.02	0.7268	814	2.42	0.8656	802	36.4%	-0.40 [-0.48, -0.32]
**Total (95% CI)**			**1218**			**1213**	**100.0%**	**-0.16 [-0.96, 0.65]**

Heterogeneity: Tau² = 0.46; Chi² = 170.35, df = 2 (P < 0.00001); I² = 99%
Test for overall effect: Z = 0.39 (P = 0.70)

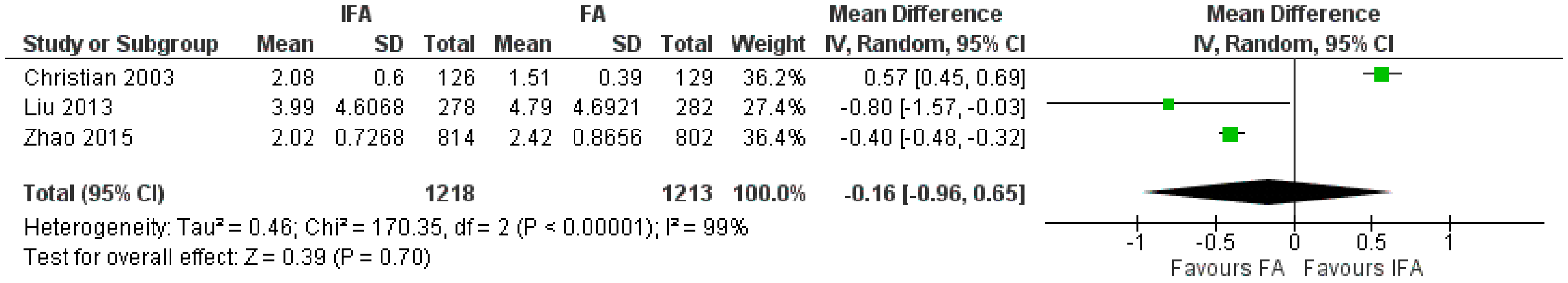

Neonatal mortality

Study or Subgroup	IFA Events	IFA Total	FA Events	FA Total	Weight	Risk Ratio M-H, Random, 95% CI
Christian 2003	24	777	24	772	32.8%	0.99 [0.57, 1.73]
Liu 2013	32	5926	29	5906	36.5%	1.10 [0.67, 1.82]
Zeng 2008	16	1154	33	1259	30.7%	0.53 [0.29, 0.96]
**Total (95% CI)**		**7857**		**7937**	**100.0%**	**0.85 [0.55, 1.31]**
Total events	72		86			

Heterogeneity: Tau² = 0.07; Chi² = 3.78, df = 2 (P = 0.15); I² = 47%
Test for overall effect: Z = 0.73 (P = 0.46)

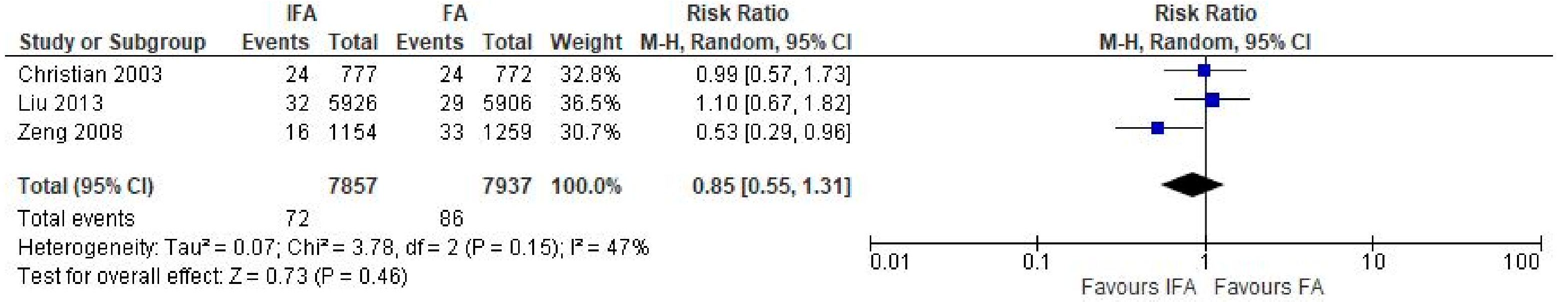

Supplementary Materials

Preterm births

Study or Subgroup	IFA Events	IFA Total	FA Events	FA Total	Weight	Risk Ratio M-H, Random, 95% CI
Christian 2003	633	635	628	628	23.0%	1.00 [0.99, 1.00]
Etheredge 2015	100	665	113	685	21.2%	0.91 [0.71, 1.17]
Liu 2013	340	5926	353	5906	22.3%	0.96 [0.83, 1.11]
Zeng 2008	59	1183	79	1282	20.0%	0.81 [0.58, 1.12]
Ziaei 2007	17	370	13	357	13.5%	1.26 [0.62, 2.56]
**Total (95% CI)**		**8779**		**8858**	**100.0%**	**0.96 [0.64, 1.44]**
Total events	1149		1186			

Heterogeneity: $Tau^2 = 0.18$; $Chi^2 = 111.03$, $df = 4$ ($P < 0.00001$); $I^2 = 96\%$

Test for overall effect: $Z = 0.20$ ($P = 0.84$)

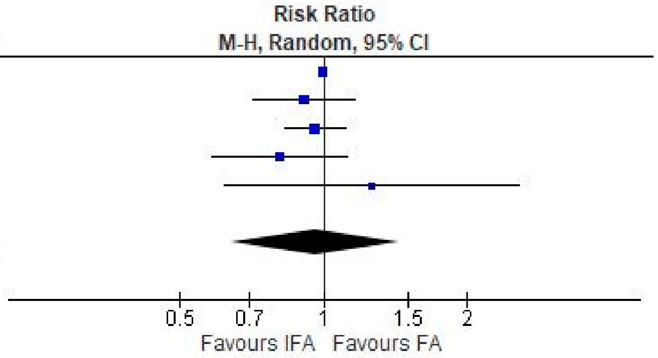

Small-for-gestational-age infants

Study or Subgroup	IFA Events	IFA Total	FA Events	FA Total	Weight	Risk Ratio M-H, Random, 95% CI
Christian 2003	328	635	363	628	34.6%	0.89 [0.81, 0.99]
Etheredge 2015 (1)	101	641	105	648	21.8%	0.97 [0.76, 1.25]
Zeng 2008	278	1565	280	1705	30.3%	1.08 [0.93, 1.26]
Ziaei 2007	57	370	36	357	13.3%	1.53 [1.03, 2.26]
**Total (95% CI)**		**3211**		**3338**	**100.0%**	**1.04 [0.87, 1.24]**
Total events	764		784			

Heterogeneity: $Tau^2 = 0.02$; $Chi^2 = 10.37$, $df = 3$ ($P = 0.02$); $I^2 = 71\%$

Test for overall effect: $Z = 0.39$ ($P = 0.69$)

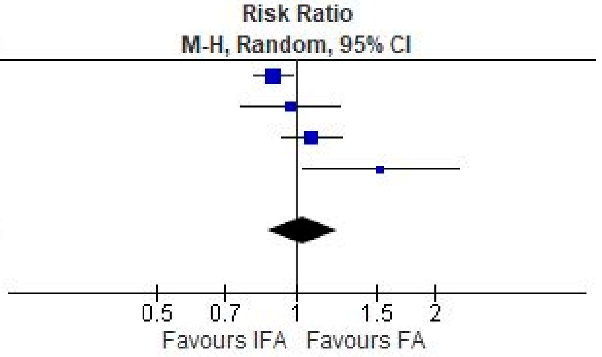

Footnotes

(1) Based on INTERGROWTH-21st Standard

Supplementary Materials

Infant mortality

	IFA		FA			Risk Ratio
**Study or Subgroup**	**Events**	**Total**	**Events**	**Total**	**Weight**	**M-H, Random, 95% CI**
Christian 2003	40	772	34	777	38.0%	1.18 [0.76, 1.85]
Etheredge 2015	20	675	16	692	18.0%	1.28 [0.67, 2.45]
Liu 2013	44	5926	45	5906	44.1%	0.97 [0.64, 1.47]
**Total (95% CI)**		**7373**		**7375**	**100.0%**	**1.10 [0.84, 1.45]**
Total events	104		95			

Heterogeneity: Tau² = 0.00; Chi² = 0.65, df = 2 (P = 0.72); I² = 0%

Test for overall effect: Z = 0.69 (P = 0.49)

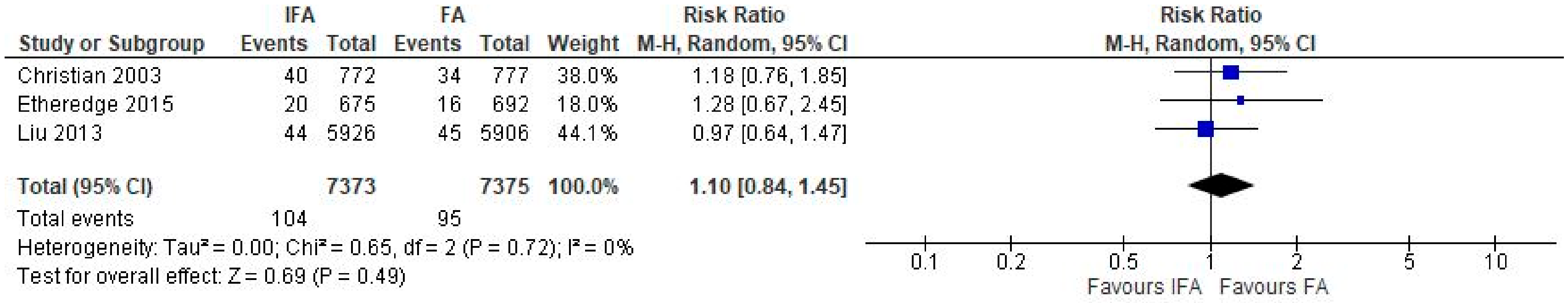

Supplementary Materials

Comparison: Multiple Micronutrient (MMN) Supplementation vs. Iron-Folic Acid (IFA) Supplementation

Maternal mortality

Study or Subgroup	log[Risk Ratio]	SE	MMN Total	Fe+/- FA Total	Weight	Risk Ratio IV, Random, 95% CI
Ashorn 2015	-0.3268	0.7603	465	460	6.3%	0.72 [0.16, 3.20]
Dewey 2009	1.1032	1.1527	354	349	2.8%	3.01 [0.31, 28.86]
Kæstel 2005	-0.5711	0.6236	520	526	9.4%	0.56 [0.17, 1.92]
Roth 2018 (MDIG)	-1.1025	1.6306	260	259	1.4%	0.33 [0.01, 8.11]
SUMMIT 2008	0.02955	0.2427	11916	11705	62.1%	1.03 [0.64, 1.66]
West 2014	0.5768	0.5577	22405	22162	11.8%	1.78 [0.60, 5.31]
Zagré 2007	0.1906	0.7652	1893	1777	6.2%	1.21 [0.27, 5.42]
**Total (95% CI)**			**37813**	**37238**	**100.0%**	**1.04 [0.71, 1.51]**

Heterogeneity: Tau² = 0.00; Chi² = 3.50, df = 6 (P = 0.74); I² = 0%

Test for overall effect: Z = 0.20 (P = 0.84)

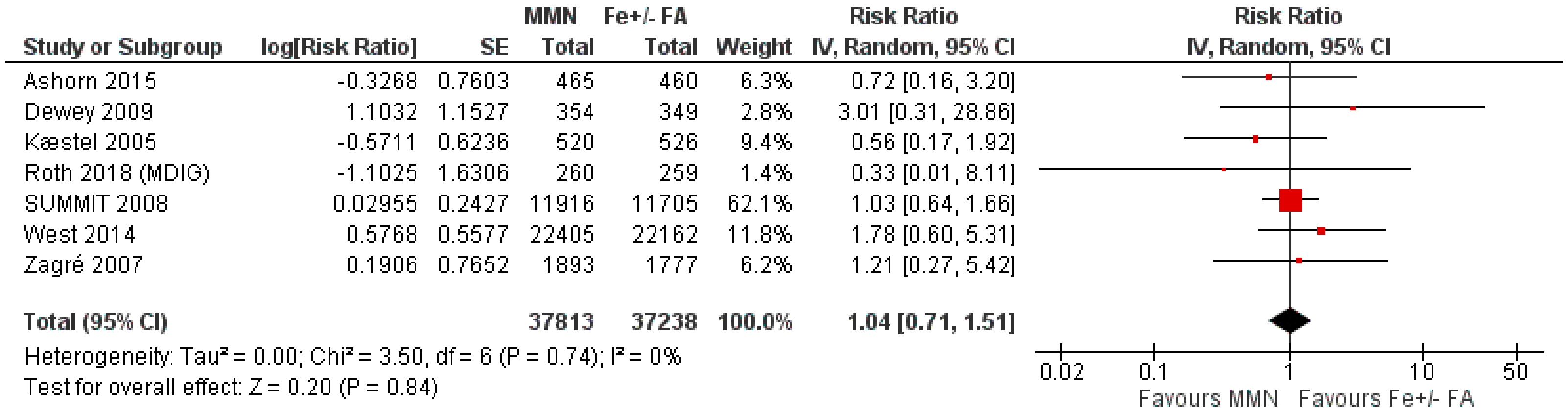

Supplementary Materials

Perinatal Mortality

Study or Subgroup	log[Risk Ratio]	SE	MMN Total	Fe with or without FA Total	Weight	Risk Ratio IV, Random, 95% CI
**2.5.1 >4 micronutrients**						
Ashorn 2015	-0.5793	0.3715	430	427	2.0%	0.56 [0.27, 1.16]
Bhutta 2009	0.2927	0.1545	372	416	7.9%	1.34 [0.99, 1.81]
Dewey 2009	-0.2374	0.4683	354	349	1.3%	0.79 [0.31, 1.97]
Fawzi 2007	-0.1278	0.0884	3982	4061	13.9%	0.88 [0.74, 1.05]
Kæstel 2005	-0.1884	0.2044	546	554	5.4%	0.83 [0.55, 1.24]
Liu 2013	-0.0619	0.1961	5942	5954	5.7%	0.94 [0.64, 1.38]
Osrin 2005	0.1914	0.2751	600	600	3.3%	1.21 [0.71, 2.08]
Ramakrishnan 2003	0.2151	0.6601	571	568	0.7%	1.24 [0.34, 4.52]
Roberfroid 2008	0.7245	0.3329	632	628	2.4%	2.06 [1.07, 3.96]
SUMMIT 2008	-0.10536	0.06651	14532	14239	16.5%	0.90 [0.79, 1.03]
Sunawang 2009	-0.1406	0.281	401	365	3.2%	0.87 [0.50, 1.51]
Tofail 2008	-0.1195	0.1834	740	738	6.3%	0.89 [0.62, 1.27]
West 2014	-0.0726	0.0399	15022	14858	19.6%	0.93 [0.86, 1.01]
Zeng 2008	0.3195	0.1942	1532	1546	5.8%	1.38 [0.94, 2.01]
**Subtotal (95% CI)**			**45656**	**45303**	**93.7%**	**0.97 [0.88, 1.07]**
Heterogeneity: Tau² = 0.01; Chi² = 19.42, df = 13 (P = 0.11); I² = 33%						
Test for overall effect: Z = 0.66 (P = 0.51)						
**2.5.2 < or equal to 4 micronutrients**						
Christian 2003	0.3436	0.1856	919	801	6.2%	1.41 [0.98, 2.03]
Dijkhuizen 2001	2.0666	1.476	48	42	0.1%	7.90 [0.44, 142.52]
**Subtotal (95% CI)**			**967**	**843**	**6.3%**	**1.79 [0.56, 5.76]**
Heterogeneity: Tau² = 0.38; Chi² = 1.34, df = 1 (P = 0.25); I² = 25%						
Test for overall effect: Z = 0.98 (P = 0.33)						
**Total (95% CI)**			**46623**	**46146**	**100.0%**	**1.00 [0.90, 1.11]**
Heterogeneity: Tau² = 0.01; Chi² = 26.13, df = 15 (P = 0.04); I² = 43%						
Test for overall effect: Z = 0.03 (P = 0.98)						
Test for subgroup differences: Chi² = 1.06, df = 1 (P = 0.30), I² = 5.7%						

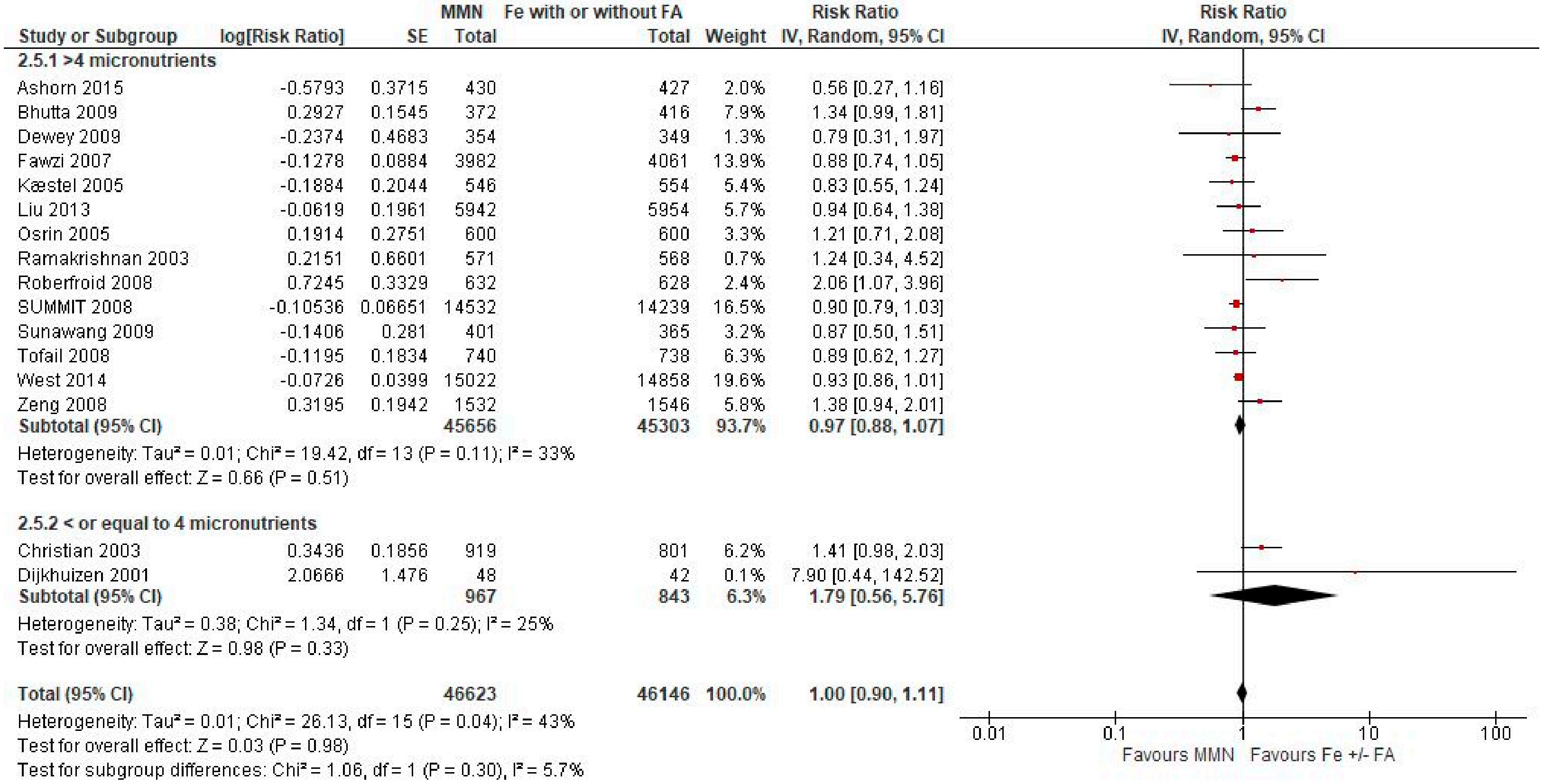

Supplementary Materials

Maternal anemia

Study or Subgroup	log[Risk Ratio]	SE	MMN Total	Fe+/- FA Total	Weight	Risk Ratio IV, Random, 95% CI
**2.2.1 >4 micronutrients**						
Ashorn 2015	0.1317	0.0789	465	460	9.2%	1.14 [0.98, 1.33]
Bhutta 2009	-0.1038	0.0969	233	261	7.7%	0.90 [0.75, 1.09]
Dewey 2009 (1)	0.5188	0.2161	291	270	2.7%	1.68 [1.10, 2.57]
Hanieh 2013	0.3454	0.2465	246	278	2.2%	1.41 [0.87, 2.29]
Huy 2009	0.292	0.3564	211	148	1.1%	1.34 [0.67, 2.69]
Liu 2013	-0.0108	0.0763	5904	5913	9.4%	0.99 [0.85, 1.15]
Moore 2012 (2)	-0.2076	0.2075	75	75	2.9%	0.81 [0.54, 1.22]
Osrin 2005	-0.1373	0.1023	537	517	7.3%	0.87 [0.71, 1.07]
Ramakrishnan 2003	0.1178	0.1295	142	148	5.6%	1.13 [0.87, 1.45]
Roberfroid 2008	0.0327	0.0954	146	156	7.8%	1.03 [0.86, 1.25]
SUMMIT 2008	0.0236	0.0249	2315	2016	13.8%	1.02 [0.98, 1.08]
West 2014	0.0108	0.0623	775	819	10.7%	1.01 [0.89, 1.14]
Zeng 2008	-0.0571	0.1319	145	149	5.5%	0.94 [0.73, 1.22]
**Subtotal (95% CI)**			**11485**	**11210**	**85.8%**	**1.02 [0.96, 1.08]**
Heterogeneity: Tau² = 0.00; Chi² = 15.99, df = 12 (P = 0.19); I² = 25%						
Test for overall effect: Z = 0.71 (P = 0.48)						
**2.2.2 < or equal to 4 micronutrients**						
Choudhury 2012	0.2099	0.1255	207	198	5.8%	1.23 [0.96, 1.58]
Christian 2003	0.3716	0.1999	183	136	3.0%	1.45 [0.98, 2.15]
Muslimatun 2001 (3)	-0.4701	0.1345	71	66	5.4%	0.62 [0.48, 0.81]
**Subtotal (95% CI)**			**461**	**400**	**14.2%**	**1.03 [0.61, 1.72]**
Heterogeneity: Tau² = 0.18; Chi² = 18.39, df = 2 (P = 0.0001); I² = 89%						
Test for overall effect: Z = 0.10 (P = 0.92)						
**Total (95% CI)**			**11946**	**11610**	**100.0%**	**1.02 [0.95, 1.10]**
Heterogeneity: Tau² = 0.01; Chi² = 34.65, df = 15 (P = 0.003); I² = 57%						
Test for overall effect: Z = 0.52 (P = 0.60)						
Test for subgroup differences: Chi² = 0.00, df = 1 (P = 0.98), I² = 0%						

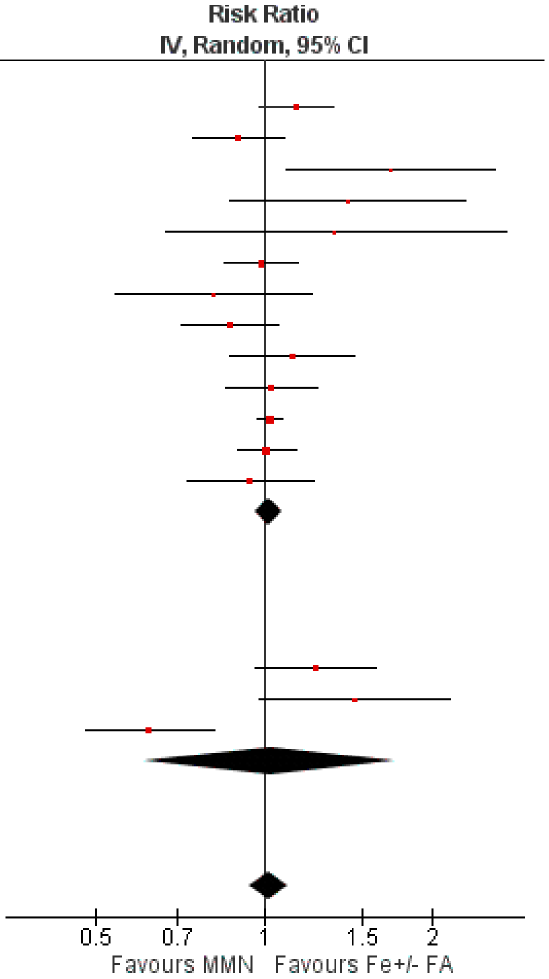

Footnotes

(1) in this paper, under reference: Adu-Afarwuah et al, (2015)

(2) In this paper, under reference: Johnson et al. (2017)

(3) Weekly MMN supplementation vs. daily iron supplementation

Supplementary Materials

Preterm births

Study or Subgroup	log[Risk Ratio]	SE	MMN Total	Fe +/- FA Total	Weight	Risk Ratio IV, Random, 95% CI
**2.17.1 >4 micronutrients**						
Ashorn 2015	-0.2132	0.2281	433	434	1.3%	0.81 [0.52, 1.26]
Bhutta 2009	0.0677	0.1358	372	456	3.1%	1.07 [0.82, 1.40]
Dewey 2009 (1)	0.4296	0.2862	305	318	0.8%	1.54 [0.88, 2.69]
Fawzi 2007	0.01	0.0532	4000	3988	9.8%	1.01 [0.91, 1.12]
Friis 2004	-0.2357	0.1847	564	542	1.8%	0.79 [0.55, 1.13]
Hanieh 2013	0.1524	0.1786	294	308	2.0%	1.16 [0.82, 1.65]
Kæstel 2005	0.0421	0.1541	279	291	2.5%	1.04 [0.77, 1.41]
Liu 2013	-0.0943	0.0786	5916	5926	6.7%	0.91 [0.78, 1.06]
Moore 2012 (2)	-0.4039	0.7551	164	146	0.1%	0.67 [0.15, 2.93]
Osrin 2005	-0.1441	0.1905	571	568	1.7%	0.87 [0.60, 1.26]
Ramakrishnan 2003	0.1336	0.2882	323	322	0.8%	1.14 [0.65, 2.01]
Roberfroid 2008	0.055	0.1438	607	604	2.8%	1.06 [0.80, 1.40]
SUMMIT 2008	-0.0005	0.0219	14373	14053	14.5%	1.00 [0.96, 1.04]
Sunawang 2009	0.0801	0.098	692	676	5.1%	1.08 [0.89, 1.31]
Tofail 2008	-0.2663	0.1333	901	910	3.2%	0.77 [0.59, 0.99]
West 2014	-0.1625	0.0309	11718	11594	13.2%	0.85 [0.80, 0.90]
Zagré 2007	0.0259	0.0587	1039	937	9.0%	1.03 [0.91, 1.15]
Zeng 2008	0.0598	0.1723	1150	1183	2.1%	1.06 [0.76, 1.49]
**Subtotal (95% CI)**			**43701**	**43256**	**80.6%**	**0.97 [0.91, 1.03]**
Heterogeneity: Tau² = 0.01; Chi² = 34.10, df = 17 (P = 0.008); I² = 50%						
Test for overall effect: Z = 0.96 (P = 0.33)						
**2.17.2 < or equal to 4 micronutrients**						
Aminisani 2009	0.2625	0.4813	89	90	0.3%	1.30 [0.51, 3.34]
Caulfield 1999	-0.0851	0.2527	521	495	1.0%	0.92 [0.56, 1.51]
Christian 2003	-0.1393	0.0895	705	635	5.7%	0.87 [0.73, 1.04]
Dijkhuizen 2001	-0.4212	0.734	48	42	0.1%	0.66 [0.16, 2.77]
Hafeez 2005 (3)	0.7885	0.3591	121	121	0.5%	2.20 [1.09, 4.45]
Merialdi 2004	0.3725	0.5699	109	113	0.2%	1.45 [0.47, 4.43]
Roth 2013 (AViDD)	-0.5782	0.4388	72	75	0.4%	0.56 [0.24, 1.33]
Roth 2018 (MDIG)	0.06	0.2685	252	247	0.9%	1.06 [0.63, 1.80]
Saaka 2009	0.0216	0.208	272	271	1.5%	1.02 [0.68, 1.54]
Sorouri 2016	-0.069	0.3615	270	270	0.5%	0.93 [0.46, 1.90]
Villar 2006	-0.0902	0.0657	4038	4042	8.1%	0.91 [0.80, 1.04]
**Subtotal (95% CI)**			**6497**	**6401**	**19.4%**	**0.93 [0.84, 1.02]**
Heterogeneity: Tau² = 0.00; Chi² = 9.46, df = 10 (P = 0.49); I² = 0%						
Test for overall effect: Z = 1.63 (P = 0.10)						
**Total (95% CI)**			**50198**	**49657**	**100.0%**	**0.96 [0.91, 1.01]**
Heterogeneity: Tau² = 0.00; Chi² = 44.15, df = 28 (P = 0.03); I² = 37%						
Test for overall effect: Z = 1.43 (P = 0.15)						
Test for subgroup differences: Chi² = 0.69, df = 1 (P = 0.41), I² = 0%						

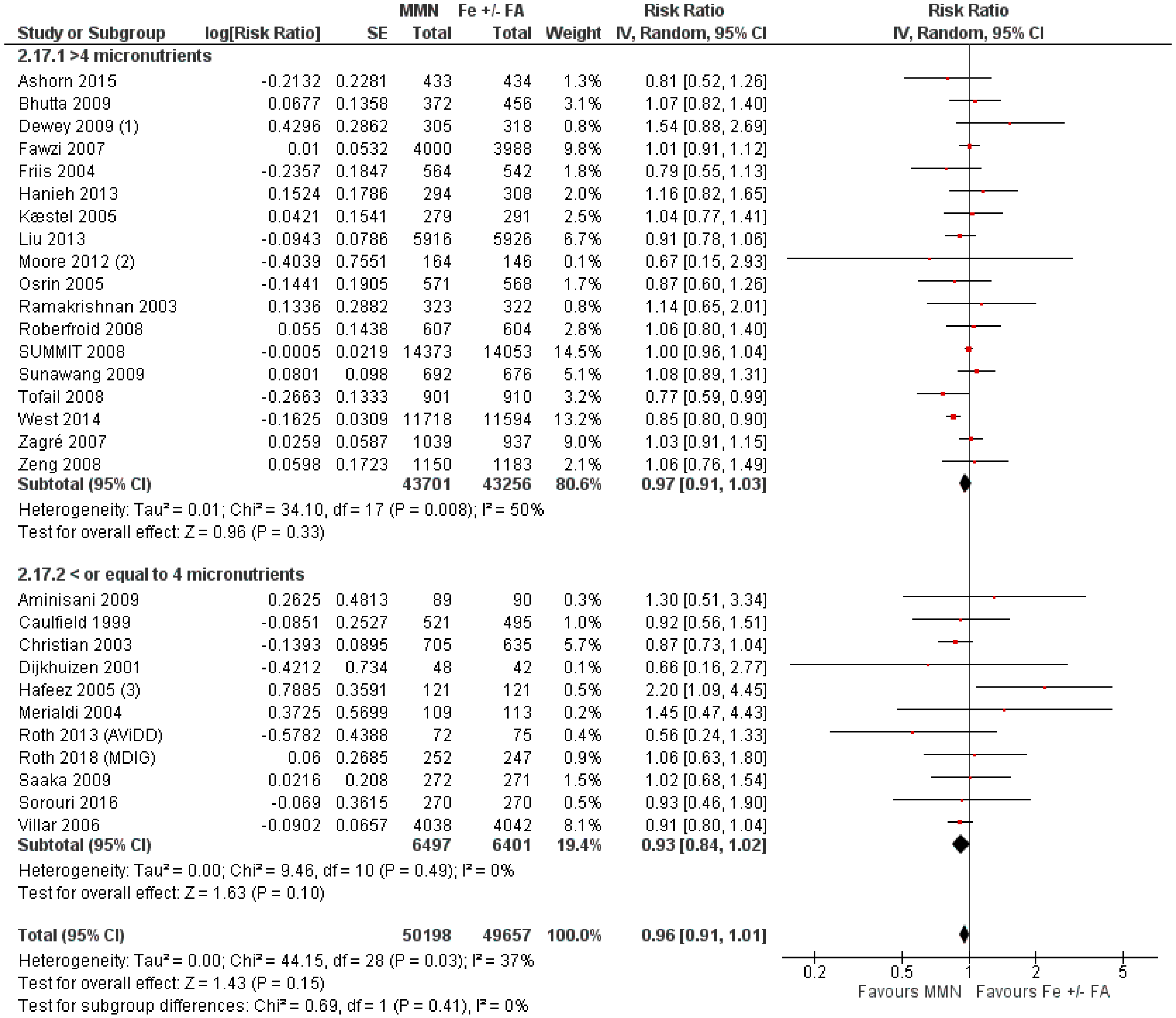

Footnotes

(1) In this paper, under reference: Adu-Afarwuah et al. (2015)

(2) In this paper, under reference: Johnson et al. (2017)

(3) *N event values for control and intervention group were rounded

Supplementary Materials

Child serum/plasma retinol concentration (umol/L)

Study or Subgroup	MMN Mean	MMN SD	MMN Total	Fe +/- FA Mean	Fe +/- FA SD	Fe +/- FA Total	Weight	Mean Difference IV, Random, 95% CI
**2.31.1 >4 micronutrients**								
Tofail 2008	0.906	0.259	349	0.86	0.252	331	33.9%	0.05 [0.01, 0.08]
**Subtotal (95% CI)**			**349**			**331**	**33.9%**	**0.05 [0.01, 0.08]**
Heterogeneity: Not applicable								
Test for overall effect: Z = 2.35 (P = 0.02)								
**2.31.2 < or equal to 4 micronutrients**								
Dijkhuizen 2001	0.62	0.16	34	0.62	0.18	33	15.3%	0.00 [-0.08, 0.08]
Muslimatun 2001	0.62	0.02	58	0.54	0.02	63	50.8%	0.08 [0.07, 0.09]
**Subtotal (95% CI)**			**92**			**96**	**66.1%**	**0.05 [-0.02, 0.13]**
Heterogeneity: Tau² = 0.00; Chi² = 3.66, df = 1 (P = 0.06); I² = 73%								
Test for overall effect: Z = 1.32 (P = 0.19)								
**Total (95% CI)**			**441**			**427**	**100.0%**	**0.06 [0.02, 0.09]**
Heterogeneity: Tau² = 0.00; Chi² = 6.47, df = 2 (P = 0.04); I² = 69%								
Test for overall effect: Z = 2.90 (P = 0.004)								
Test for subgroup differences: Chi² = 0.01, df = 1 (P = 0.91), I² = 0%								

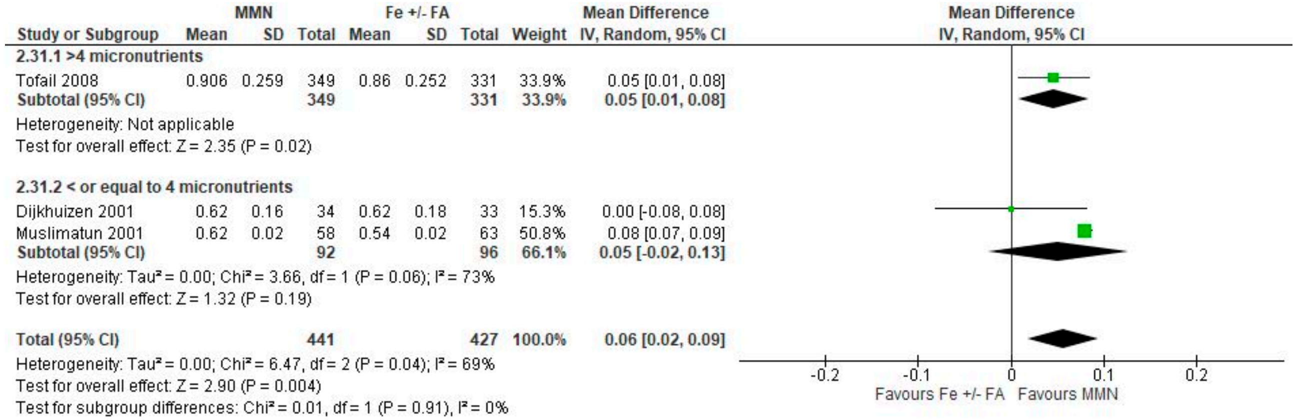

Child Wasting

Study or Subgroup	MMN Events	MMN Total	Fe +/- FA Events	Fe +/- FA Total	Weight	Risk Ratio M-H, Random, 95% CI
Huy 2009	18	203	25	211	6.1%	0.75 [0.42, 1.33]
Osrin 2005	29	462	25	455	7.4%	1.14 [0.68, 1.92]
Roth 2018 (MDIG) (1)	16	151	9	172	3.4%	2.03 [0.92, 4.45]
West 2014	820	3728	808	3689	81.8%	1.00 [0.92, 1.09]
Zeng 2008 (2)	4	285	5	315	1.2%	0.88 [0.24, 3.26]
**Total (95% CI)**		**4829**		**4842**	**100.0%**	**1.02 [0.88, 1.18]**
Total events	887		872			
Heterogeneity: Tau² = 0.00; Chi² = 4.32, df = 4 (P = 0.36); I² = 7%						
Test for overall effect: Z = 0.24 (P = 0.81)						

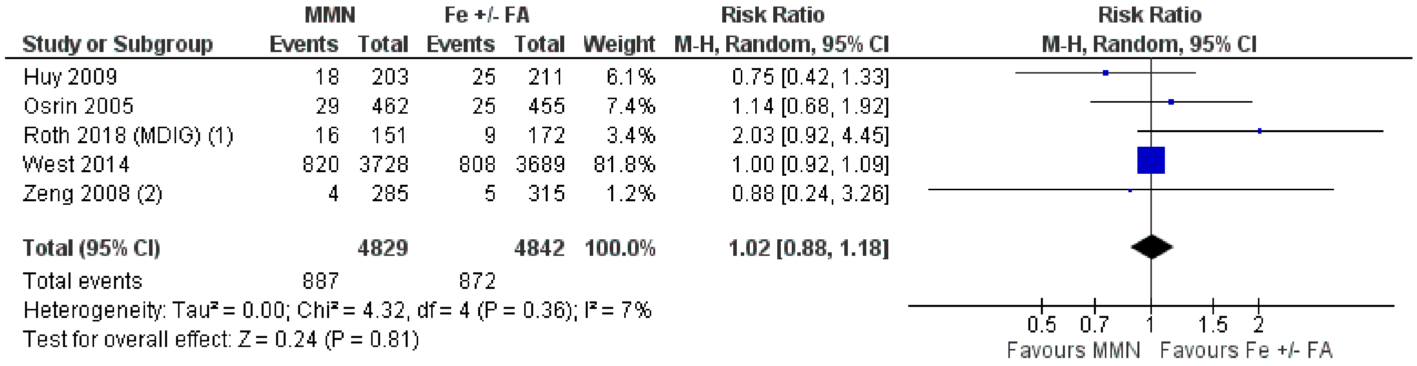

Footnotes

(1) measured at one year of age

(2) followup measured at 30 months

Supplementary Materials

Child stunting

Study or Subgroup	MMN Events	MMN Total	Fe +/- FA Events	Fe +/- FA Total	Weight	Risk Ratio M-H, Random, 95% CI
Hanieh 2013	18	256	18	269	1.5%	1.05 [0.56, 1.97]
Huy 2009	42	203	62	211	4.8%	0.70 [0.50, 0.99]
Osrin 2005	262	462	272	455	26.3%	0.95 [0.85, 1.06]
Roth 2018 (MDIG) (1)	26	151	28	172	2.4%	1.06 [0.65, 1.72]
Tofail 2008	199	550	165	518	15.5%	1.14 [0.96, 1.34]
West 2014	1842	3728	1789	3689	46.0%	1.02 [0.97, 1.07]
Zeng 2008 (2)	35	285	47	315	3.4%	0.82 [0.55, 1.24]
**Total (95% CI)**		**5635**		**5629**	**100.0%**	**0.99 [0.92, 1.07]**
Total events	2424		2381			

Heterogeneity: Tau² = 0.00; Chi² = 8.58, df = 6 (P = 0.20); I² = 30%
Test for overall effect: Z = 0.17 (P = 0.86)

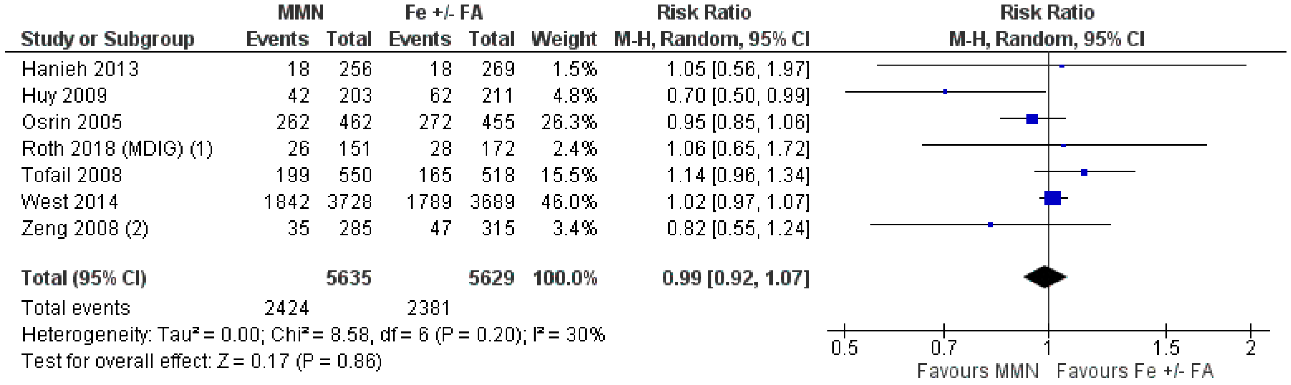

Footnotes
(1) measured at one year of age
(2) followup measured at 30 months

Child underweight status

Study or Subgroup	MMN Events	MMN Total	Fe with or without FA Events	Fe with or without FA Total	Weight	Risk Ratio M-H, Random, 95% CI
Huy 2009	67	203	92	211	17.0%	0.76 [0.59, 0.97]
Osrin 2005	169	462	171	453	27.6%	0.97 [0.82, 1.15]
West 2014	1808	3728	1763	3689	53.5%	1.01 [0.97, 1.06]
Zeng 2008	8	267	11	306	1.8%	0.83 [0.34, 2.04]
**Total (95% CI)**		**4660**		**4659**	**100.0%**	**0.95 [0.84, 1.07]**
Total events	2052		2037			

Heterogeneity: Tau² = 0.01; Chi² = 5.48, df = 3 (P = 0.14); I² = 45%
Test for overall effect: Z = 0.83 (P = 0.41)

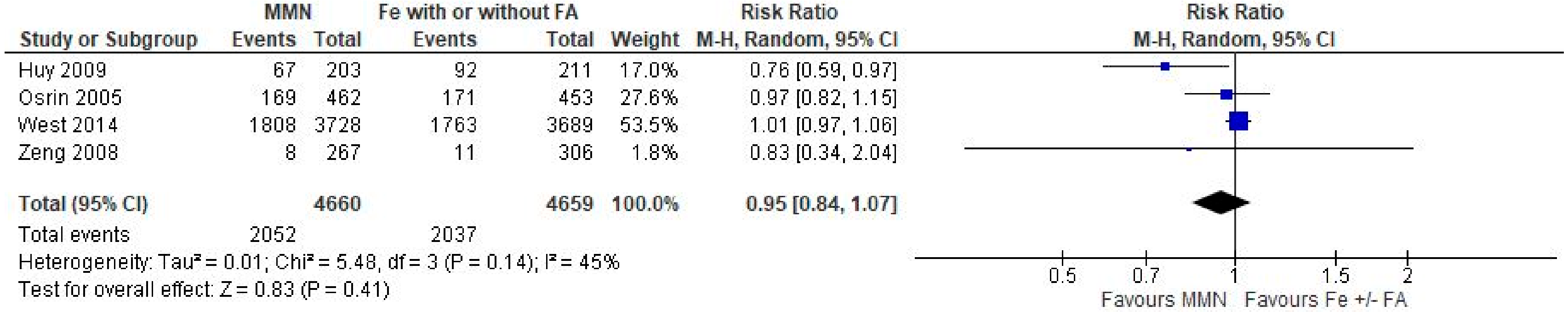

Supplementary Materials

Child development outcome – executive function

Study or Subgroup	MMN Mean	MMN SD	MMN Total	Fe +/- FA Mean	Fe +/- FA SD	Fe +/- FA Total	Weight	Std. Mean Difference IV, Random, 95% CI
Christian 2003	47	20.9	218	47.1	21	103	11.3%	-0.00 [-0.24, 0.23]
Osrin 2005	2.3	6	402	1.7	6.2	403	32.6%	0.10 [-0.04, 0.24]
Zeng 2008	95	11.5	708	93.9	10.9	677	56.1%	0.10 [-0.01, 0.20]
**Total (95% CI)**			**1328**			**1183**	**100.0%**	**0.09 [0.01, 0.17]**

Heterogeneity: Tau² = 0.00; Chi² = 0.66, df = 2 (P = 0.72); I² = 0%
Test for overall effect: Z = 2.15 (P = 0.03)

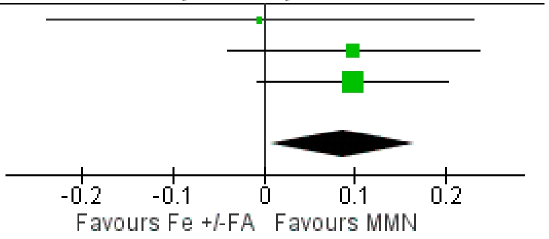

Child development outcome – general intelligence

Study or Subgroup	MMN Mean	MMN SD	MMN Total	Fe +/- FA Mean	Fe +/- FA SD	Fe +/- FA Total	Weight	Std. Mean Difference IV, Random, 95% CI
**2.25.1 >4 micronutrients**								
Hanieh 2013 (1)	101.2	9.9	335	100.2	11.4	351	12.4%	0.09 [-0.06, 0.24]
Osrin 2005 (2)	77.6	12.3	404	76.4	14	409	13.8%	0.09 [-0.05, 0.23]
Tofail 2008 (3)	13.3	7	354	13.2	6.6	356	12.7%	0.01 [-0.13, 0.16]
West 2014 (4)	76.2	7	4287	76.5	7.8	4242	33.3%	-0.04 [-0.08, 0.00]
Zeng 2008 (5)	103.65	41.9135	351	102.44	45.1734	393	13.0%	0.03 [-0.12, 0.17]
**Subtotal (95% CI)**			**5731**			**5751**	**85.2%**	**0.01 [-0.05, 0.08]**
Heterogeneity: Tau² = 0.00; Chi² = 6.17, df = 4 (P = 0.19); I² = 35%								
Test for overall effect: Z = 0.43 (P = 0.67)								
**2.25.2 < or equal to 4 micronutrients**								
Christian 2003 (6)	49.3	9.7	218	51.7	8.5	103	6.2%	-0.26 [-0.49, -0.02]
Merialdi 2004 (7)	91.9	10	85	92.3	10.1	96	4.2%	-0.04 [-0.33, 0.25]
Muslimatun 2001 (8)	94.7	1.7	94	94.5	1.7	94	4.4%	0.12 [-0.17, 0.40]
**Subtotal (95% CI)**			**397**			**293**	**14.8%**	**-0.07 [-0.29, 0.15]**
Heterogeneity: Tau² = 0.02; Chi² = 4.05, df = 2 (P = 0.13); I² = 51%								
Test for overall effect: Z = 0.64 (P = 0.52)								
**Total (95% CI)**			**6128**			**6044**	**100.0%**	**0.00 [-0.06, 0.07]**

Heterogeneity: Tau² = 0.00; Chi² = 11.02, df = 7 (P = 0.14); I² = 36%
Test for overall effect: Z = 0.12 (P = 0.91)
Test for subgroup differences: Chi² = 0.53, df = 1 (P = 0.46), I² = 0%

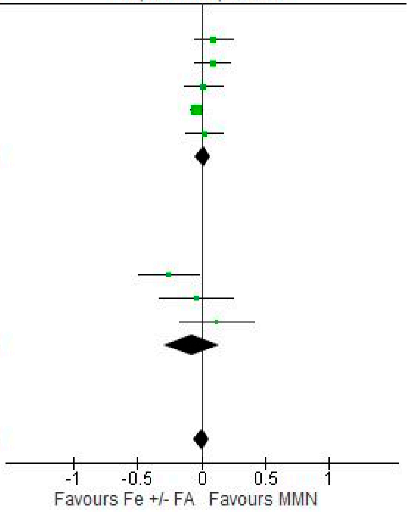

Footnotes
(1) Bayley Scale III
(2) Full scale IQ
(3) Cover tests
(4) Bayley Scale III
(5) Mental Development raw score
(6) UNIT Score
(7) Wechsler Preschool and Primary Scale of Intelligence
(8) BSID-I

Supplementary Materials

Child development outcome – verbal comprehension and language

	MMN			Fe +/- FA				Std. Mean Difference
Study or Subgroup	Mean	SD	Total	Mean	SD	Total	Weight	IV, Random, 95% CI
**2.26.1 >4 micronutrients**								
Hanieh 2013 (1)	94.8	7.7	335	94	8	351	24.7%	0.10 [-0.05, 0.25]
West 2014 (2)	85.3	8.9	4287	86.2	10	4242	32.6%	-0.10 [-0.14, -0.05]
Zeng 2008	103.6	16.1	708	101.7	15.1	677	28.5%	0.12 [0.02, 0.23]
**Subtotal (95% CI)**			**5330**			**5270**	**85.8%**	**0.03 [-0.13, 0.20]**
Heterogeneity: Tau² = 0.02; Chi² = 18.40, df = 2 (P = 0.0001); I² = 89%								
Test for overall effect: Z = 0.41 (P = 0.68)								
**2.26.2 < or equal to 4 micronutrients**								
Merialdi 2004 (3)	85.3	9.8	85	86.2	10.3	96	14.2%	-0.09 [-0.38, 0.20]
**Subtotal (95% CI)**			**85**			**96**	**14.2%**	**-0.09 [-0.38, 0.20]**
Heterogeneity: Not applicable								
Test for overall effect: Z = 0.60 (P = 0.55)								
**Total (95% CI)**			**5415**			**5366**	**100.0%**	**0.02 [-0.13, 0.16]**
Heterogeneity: Tau² = 0.02; Chi² = 18.45, df = 3 (P = 0.0004); I² = 84%								
Test for overall effect: Z = 0.22 (P = 0.83)								
Test for subgroup differences: Chi² = 0.52, df = 1 (P = 0.47), I² = 0%								

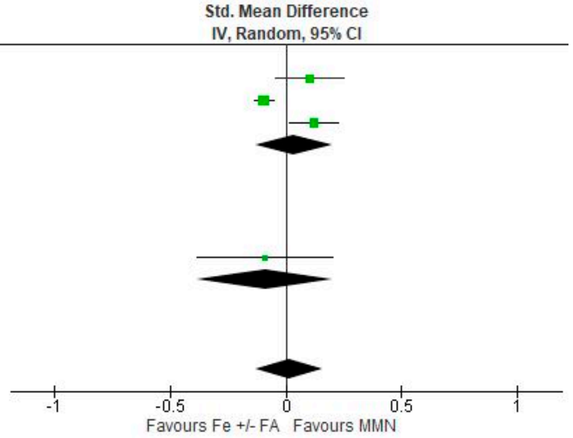

Footnotes

(1) *Bayley Scale III

(2) *Bayley Scale III

(3) Wechsler Preschool and Primary Scale of Intelligence

Supplementary Materials

Child development outcome – motor function

Study or Subgroup	MMN Mean	MMN SD	MMN Total	IFA Mean	IFA SD	IFA Total	Weight	Std. Mean Difference IV, Random, 95% CI
**2.27.1 >4 micronutrients**								
Hanieh 2013 (1)	112.5	18.4	335	112.3	18.2	351	15.4%	0.01 [-0.14, 0.16]
Tofail 2008 (2)	104.5297	16.3937	701	101.7335	15.224	707	16.7%	0.18 [0.07, 0.28]
West 2014 (3)	90.1	7.4	4287	90.7	7.8	4242	17.8%	-0.08 [-0.12, -0.04]
Zeng 2008 (4)	45.64	20.4805	351	45.3	22.0826	393	15.6%	0.02 [-0.13, 0.16]
**Subtotal (95% CI)**			**5674**			**5693**	**65.5%**	**0.03 [-0.11, 0.16]**
Heterogeneity: Tau² = 0.02; Chi² = 20.66, df = 3 (P = 0.0001); I² = 85%								
Test for overall effect: Z = 0.41 (P = 0.68)								
**2.27.2 < or equal to 4 micronutrients**								
Christian 2003 (5)	8.65	6.14	218	6.78	4.93	103	12.7%	0.32 [0.09, 0.56]
Merialdi 2004 (6)	96.2	12.4	85	95.6	14.9	96	11.0%	0.04 [-0.25, 0.34]
Muslimatun 2001 (7)	101.3	1.9	94	102.8	1.9	94	10.8%	-0.79 [-1.08, -0.49]
**Subtotal (95% CI)**			**397**			**293**	**34.5%**	**-0.14 [-0.78, 0.51]**
Heterogeneity: Tau² = 0.31; Chi² = 33.59, df = 2 (P < 0.00001); I² = 94%								
Test for overall effect: Z = 0.41 (P = 0.68)								
**Total (95% CI)**			**6071**			**5986**	**100.0%**	**-0.02 [-0.17, 0.13]**
Heterogeneity: Tau² = 0.03; Chi² = 54.36, df = 6 (P < 0.00001); I² = 89%								
Test for overall effect: Z = 0.25 (P = 0.80)								
Test for subgroup differences: Chi² = 0.24, df = 1 (P = 0.63), I² = 0%								

Std. Mean Difference IV, Random, 95% CI

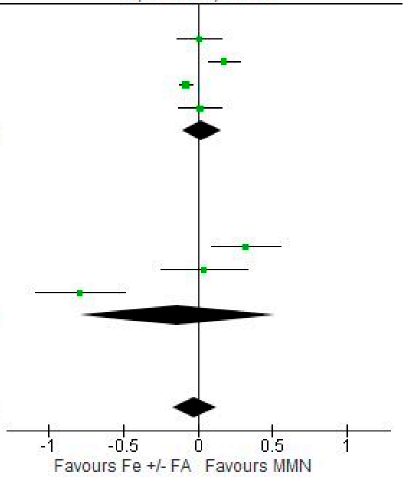

Footnotes

(1) Bayley Scale III

(2) BSID

(3) Bayley Scale III

(4) Psychomotor development raw score

(5) Movement Assessment Battery Children (MABC)

(6) Spanish Adaptation of Vineland Adaptive Behaviour Scales

(7) BSID-I

Maternal iron-deficiency anemia

Study or Subgroup	log[Risk Ratio]	SE	MMN Total	Fe with or without FA Total	Weight	Risk Ratio IV, Random, 95% CI
**2.3.1 >4 micronutrients**						
Dewey 2009	0.7136	0.3364	291	264	25.9%	2.04 [1.06, 3.95]
Hanieh 2013	-1.9317	0.7475	242	263	11.4%	0.14 [0.03, 0.63]
Ramakrishnan 2003	0.1214	0.1351	142	148	35.4%	1.13 [0.87, 1.47]
**Subtotal (95% CI)**			**675**	**675**	**72.7%**	**0.92 [0.37, 2.26]**
Heterogeneity: Tau² = 0.47; Chi² = 10.61, df = 2 (P = 0.005); I² = 81%						
Test for overall effect: Z = 0.19 (P = 0.85)						
**2.3.2 < or equal to 4 micronutrients**						
Christian 2003	0.375	0.3081	135	110	27.3%	1.45 [0.80, 2.66]
**Subtotal (95% CI)**			**135**	**110**	**27.3%**	**1.45 [0.80, 2.66]**
Heterogeneity: Not applicable						
Test for overall effect: Z = 1.22 (P = 0.22)						
**Total (95% CI)**			**810**	**785**	**100.0%**	**1.12 [0.62, 2.02]**
Heterogeneity: Tau² = 0.24; Chi² = 11.08, df = 3 (P = 0.01); I² = 73%						
Test for overall effect: Z = 0.36 (P = 0.72)						
Test for subgroup differences: Chi² = 0.69, df = 1 (P = 0.41), I² = 0%						

Risk Ratio IV, Random, 95% CI

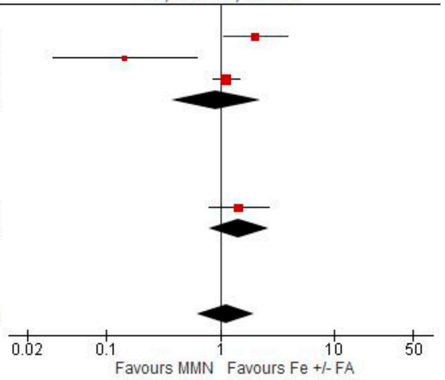

Supplementary Materials

Maternal serum/plasma hemoglobin concentration (g/L)

Study or Subgroup	MMN Mean	MMN SD	MMN Total	Fe +/- FA Mean	Fe +/- FA SD	Fe +/- FA Total	Weight	Mean Difference IV, Random, 95% CI
**2.6.1 >4 micronutrients**								
Bhutta 2009	109	16	233	109	16	261	5.7%	0.00 [-2.83, 2.83]
Dewey 2009	117	12	291	120	11	270	7.0%	-3.00 [-4.90, -1.10]
Fawzi 2007	121	20.0481	3098	119	20.0481	3078	8.0%	2.00 [1.00, 3.00]
Hanieh 2013	123.4	12.4	249	124.9	12.2	269	6.7%	-1.50 [-3.62, 0.62]
Liu 2013	123	9	5904	122	9	5913	8.4%	1.00 [0.68, 1.32]
Osrin 2005	118	12	533	118	14	517	7.4%	0.00 [-1.58, 1.58]
Ramakrishnan 2003	104.2	12.0995	176	108.1	11.7198	185	6.2%	-3.90 [-6.36, -1.44]
Tofail 2008	114.9	12.7424	977	115	12.7096	951	7.9%	-0.10 [-1.24, 1.04]
West 2014	110.6	10.3	745	111.2	10.3	775	7.9%	-0.60 [-1.64, 0.44]
Zeng 2008	111.8	14.1	145	110.1	14.8	149	5.1%	1.70 [-1.60, 5.00]
**Subtotal (95% CI)**			**12351**			**12368**	**70.3%**	**-0.30 [-1.27, 0.67]**
Heterogeneity: Tau² = 1.67; Chi² = 51.15, df = 9 (P < 0.00001); I² = 82%								
Test for overall effect: Z = 0.61 (P = 0.54)								
**2.6.2 < or equal to 4 micronutrients**								
Caulfield 1999	114	13	325	115	13	320	6.8%	-1.00 [-3.01, 1.01]
Choudhury 2012	109.5	12.9	207	112	11.2	198	6.4%	-2.50 [-4.85, -0.15]
Christian 2003	114	16	132	116	14	105	4.5%	-2.00 [-5.82, 1.82]
Dijkhuizen 2001	106	14	34	109	12	33	2.6%	-3.00 [-9.24, 3.24]
Muslimatun 2001 (1)	3.7	1.7	71	-0.7	1.6	53	8.3%	4.40 [3.82, 4.98]
Semba 2001 (2)	4.7	36.5	63	7.3	16.59	52	1.2%	-2.60 [-12.68, 7.48]
**Subtotal (95% CI)**			**832**			**761**	**29.7%**	**-0.72 [-4.44, 3.00]**
Heterogeneity: Tau² = 16.88; Chi² = 66.96, df = 5 (P < 0.00001); I² = 93%								
Test for overall effect: Z = 0.38 (P = 0.70)								
**Total (95% CI)**			**13183**			**13129**	**100.0%**	**-0.34 [-1.53, 0.86]**
Heterogeneity: Tau² = 4.38; Chi² = 199.02, df = 15 (P < 0.00001); I² = 92%								
Test for overall effect: Z = 0.55 (P = 0.58)								
Test for subgroup differences: Chi² = 0.05, df = 1 (P = 0.83), I² = 0%								

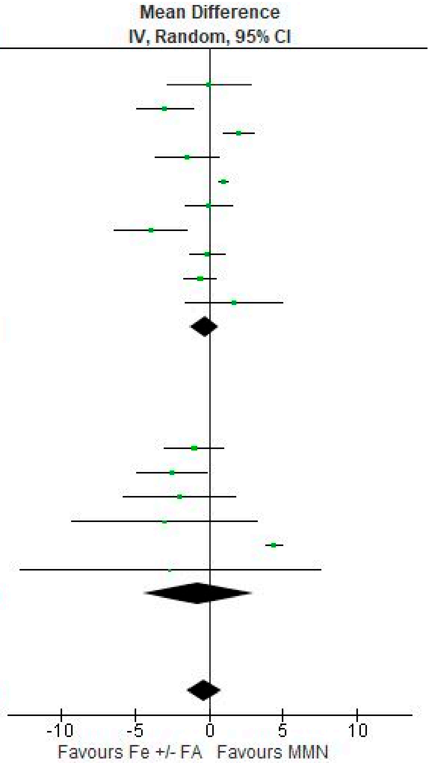

Footnotes

(1) Weekly MMN supplementation vs. daily IFA supplementation

(2) SEM converted to SD; change score from enrollment to delivery was used

Supplementary Materials

Maternal serum/plasma ferritin concentration (ug/L)

Study or Subgroup	MMN Mean	MMN SD	MMN Total	Fe with or without FA Mean	Fe with or without FA SD	Fe with or without FA Total	Weight	Mean Difference IV, Random, 95% CI
**2.7.1 >4 micronutrients**								
Bhutta 2009 (1)	0.4	0.372	233	0.489	0.391	261	9.8%	-0.09 [-0.16, -0.02]
Hanieh 2013	21.7	0.3309	250	34.5	0.3309	269	9.8%	-12.80 [-12.86, -12.74]
Liu 2013	15	12.6121	274	16.7	11.9435	278	9.6%	-1.70 [-3.75, 0.35]
Osrin 2005	25	14.69	101	29	20.7289	123	9.1%	-4.00 [-8.65, 0.65]
Ramakrishnan 2003 (2)	4.06	3.1947	142	4.35	3.5089	148	9.7%	-0.29 [-1.06, 0.48]
Tofail 2008	16.1	10.3407	212	16.1	11.1845	213	9.6%	0.00 [-2.05, 2.05]
West 2014	37.8	36.6	740	41.1	36.2	772	9.3%	-3.30 [-6.97, 0.37]
**Subtotal (95% CI)**			**1952**			**2064**	**66.9%**	**-3.17 [-9.87, 3.53]**
Heterogeneity: Tau² = 80.14; Chi² = 80221.69, df = 6 (P < 0.00001); I² = 100%								
Test for overall effect: Z = 0.93 (P = 0.35)								
**2.7.2 < or equal to 4 micronutrients**								
Caulfield 1999	17.6	22.5185	325	17.8	22.74	320	9.4%	-0.20 [-3.69, 3.29]
Christian 2003	16.12	2.16	158	13.46	2.05	126	9.7%	2.66 [2.17, 3.15]
Dijkhuizen 2001	20.23	29.01	34	22.47	47.48	33	4.5%	-2.24 [-21.15, 16.67]
Muslimatun 2001 (3)	-7.1	9.7171	71	-3	9.356	66	9.4%	-4.10 [-7.29, -0.91]
**Subtotal (95% CI)**			**588**			**545**	**33.1%**	**-0.41 [-4.53, 3.70]**
Heterogeneity: Tau² = 11.99; Chi² = 19.30, df = 3 (P = 0.0002); I² = 84%								
Test for overall effect: Z = 0.20 (P = 0.84)								
**Total (95% CI)**			**2540**			**2609**	**100.0%**	**-2.37 [-7.84, 3.10]**
Heterogeneity: Tau² = 79.79; Chi² = 81860.07, df = 10 (P < 0.00001); I² = 100%								
Test for overall effect: Z = 0.85 (P = 0.40)								
Test for subgroup differences: Chi² = 0.47, df = 1 (P = 0.49), I² = 0%								

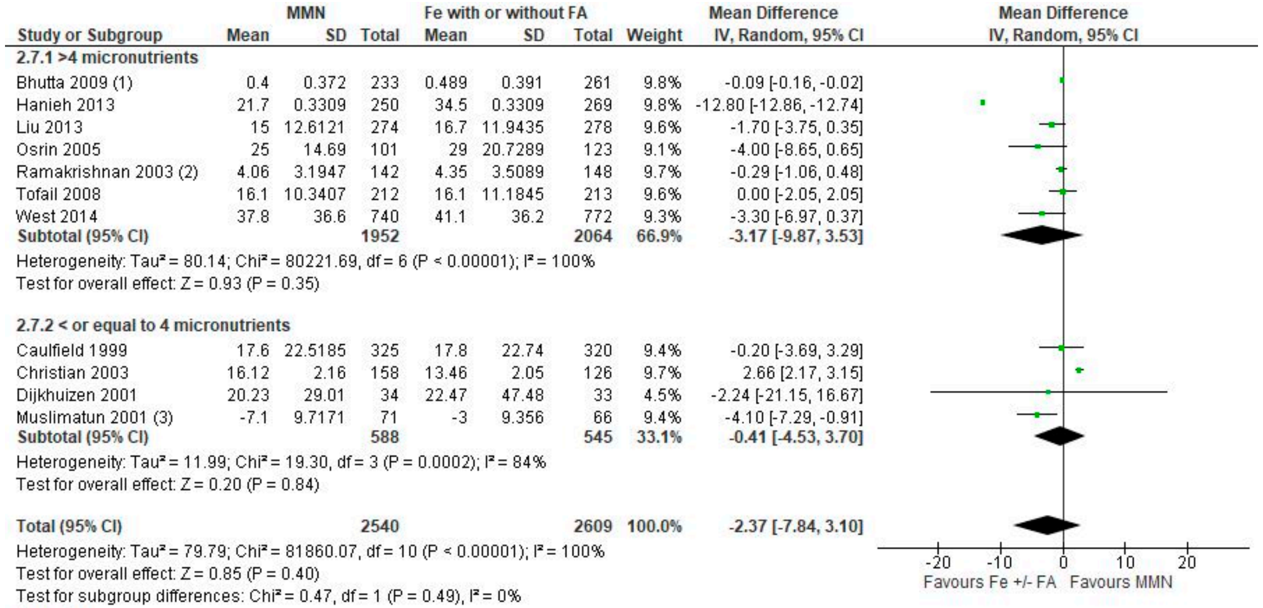

Footnotes

(1) *values seem quite different from other studies

(2) Presented as log [serum ferritin]

(3) Weekly MMN supplementation vs. daily IFA supplementation

Supplementary Materials

Maternal iron deficiency

Study or Subgroup	MMN Events	MMN Total	Fe +/- FA Events	Fe +/- FA Total	Weight	Risk Ratio M-H, Random, 95% CI
**2.8.1 >4 micronutrients**						
Hanieh 2013	69	243	31	263	30.7%	2.41 [1.64, 3.54]
Liu 2013	117	274	98	278	36.2%	1.21 [0.98, 1.49]
**Subtotal (95% CI)**		**517**		**541**	**66.9%**	**1.68 [0.85, 3.32]**
Total events	186		129			
Heterogeneity: Tau² = 0.22; Chi² = 9.69, df = 1 (P = 0.002); I² = 90%						
Test for overall effect: Z = 1.48 (P = 0.14)						
**2.8.2 < or equal to 4 micronutrients**						
Muslimatun 2001 (1)	39	71	30	53	33.1%	0.97 [0.71, 1.33]
**Subtotal (95% CI)**		**71**		**53**	**33.1%**	**0.97 [0.71, 1.33]**
Total events	39		30			
Heterogeneity: Not applicable						
Test for overall effect: Z = 0.19 (P = 0.85)						
**Total (95% CI)**		**588**		**594**	**100.0%**	**1.39 [0.88, 2.20]**
Total events	225		159			
Heterogeneity: Tau² = 0.14; Chi² = 14.25, df = 2 (P = 0.0008); I² = 86%						
Test for overall effect: Z = 1.40 (P = 0.16)						
Test for subgroup differences: Chi² = 2.02, df = 1 (P = 0.16), I² = 50.5%						

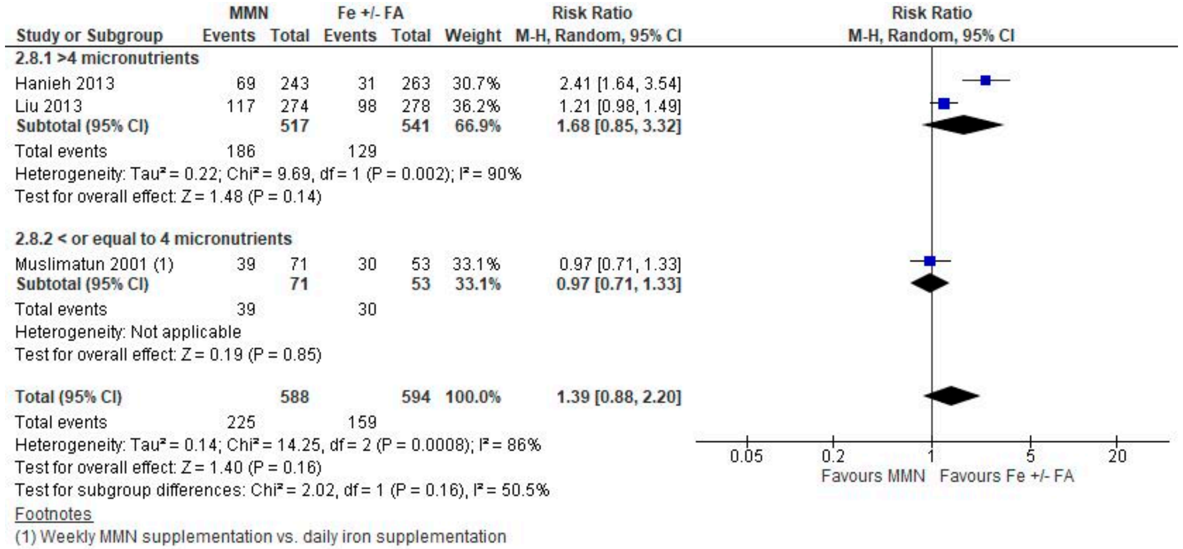

Footnotes

(1) Weekly MMN supplementation vs. daily iron supplementation

Maternal serum/plasma transferrin receptor concentration (mg/L)

Study or Subgroup	MMN Mean	MMN SD	MMN Total	Fe with or without FA Mean	Fe with or without FA SD	Fe with or without FA Total	Weight	Mean Difference IV, Random, 95% CI
**2.9.1 >4 micronutrients**								
Dewey 2009	4.6	1.7	291	4	1.3	266	16.1%	0.60 [0.35, 0.85]
Hanieh 2013 (1)	3.58	0.2942	249	3.54	0.2942	269	27.9%	0.04 [-0.01, 0.09]
Liu 2013 (2)	4.34	4.6245	274	3.99	4.6068	278	3.4%	0.35 [-0.42, 1.12]
West 2014	5.4	2.3	233	5.3	2.3	251	9.2%	0.10 [-0.31, 0.51]
**Subtotal (95% CI)**			**1047**			**1064**	**56.6%**	**0.26 [-0.09, 0.61]**
Heterogeneity: Tau² = 0.09; Chi² = 19.06, df = 3 (P = 0.0003); I² = 84%								
Test for overall effect: Z = 1.45 (P = 0.15)								
**2.9.2 < or equal to 4 micronutrients**								
Caulfield 1999	16.6	7.6	14	13.5	3.5	17	0.1%	3.10 [-1.21, 7.41]
Christian 2003	1.55	0.48	158	1.51	0.39	126	25.5%	0.04 [-0.06, 0.14]
Muslimatun 2001 (3)	0.43	0.5915	71	0.56	0.6509	53	17.7%	-0.13 [-0.35, 0.09]
**Subtotal (95% CI)**			**243**			**196**	**43.4%**	**-0.02 [-0.22, 0.18]**
Heterogeneity: Tau² = 0.01; Chi² = 3.82, df = 2 (P = 0.15); I² = 48%								
Test for overall effect: Z = 0.18 (P = 0.86)								
**Total (95% CI)**			**1290**			**1260**	**100.0%**	**0.12 [-0.03, 0.27]**
Heterogeneity: Tau² = 0.02; Chi² = 23.82, df = 6 (P = 0.0006); I² = 75%								
Test for overall effect: Z = 1.56 (P = 0.12)								
Test for subgroup differences: Chi² = 1.81, df = 1 (P = 0.18), I² = 44.9%								

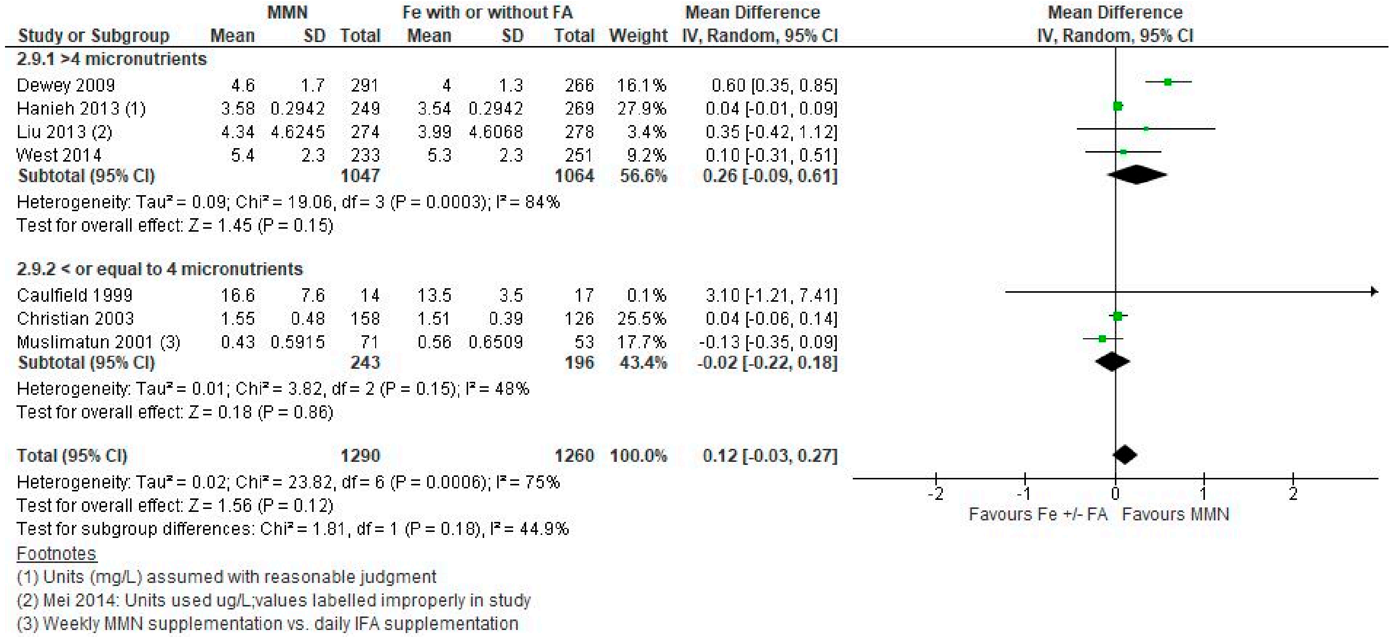

Footnotes

(1) Units (mg/L) assumed with reasonable judgment

(2) Mei 2014: Units used ug/L;values labelled improperly in study

(3) Weekly MMN supplementation vs. daily IFA supplementation

Supplementary Materials

Maternal serum/plasma retinol concentration (umol/L)

Study or Subgroup	MMN Mean	MMN SD	MMN Total	Fe with or without FA Mean	Fe with or without FA SD	Fe with or without FA Total	Weight	Mean Difference IV, Random, 95% CI
**2.10.1 >4 micronutrients**								
Bhutta 2009 (1)	43.6	25.8	466	43.3	21.4	522	0.0%	0.30 [-2.68, 3.28]
Osrin 2005	1.39	0.33	45	1.2	0.39	56	10.7%	0.19 [0.05, 0.33]
Ramakrishnan 2003	0.98	0.57	95	1	0.52	101	9.6%	-0.02 [-0.17, 0.13]
West 2014	1.08	0.33	746	0.99	0.33	774	24.6%	0.09 [0.06, 0.12]
**Subtotal (95% CI)**			**1352**			**1453**	**44.9%**	**0.09 [0.02, 0.16]**
Heterogeneity: Tau² = 0.00; Chi² = 3.95, df = 3 (P = 0.27); I² = 24%								
Test for overall effect: Z = 2.68 (P = 0.007)								
**2.10.2 < or equal to 4 micronutrients**								
Dijkhuizen 2001 (2)	0.9	0.24	34	0.8	0.29	33	11.9%	0.10 [-0.03, 0.23]
Muslimatun 2001 (3)	0.97	0.03	71	0.79	0.07	53	25.9%	0.18 [0.16, 0.20]
Semba 2001 (4)	0.86	0.24	63	0.78	0.22	52	17.3%	0.08 [-0.00, 0.16]
**Subtotal (95% CI)**			**168**			**138**	**55.1%**	**0.13 [0.06, 0.21]**
Heterogeneity: Tau² = 0.00; Chi² = 6.41, df = 2 (P = 0.04); I² = 69%								
Test for overall effect: Z = 3.41 (P = 0.0006)								
**Total (95% CI)**			**1520**			**1591**	**100.0%**	**0.11 [0.05, 0.17]**
Heterogeneity: Tau² = 0.00; Chi² = 29.42, df = 6 (P < 0.0001); I² = 80%								
Test for overall effect: Z = 3.74 (P = 0.0002)								
Test for subgroup differences: Chi² = 0.65, df = 1 (P = 0.42), I² = 0%								

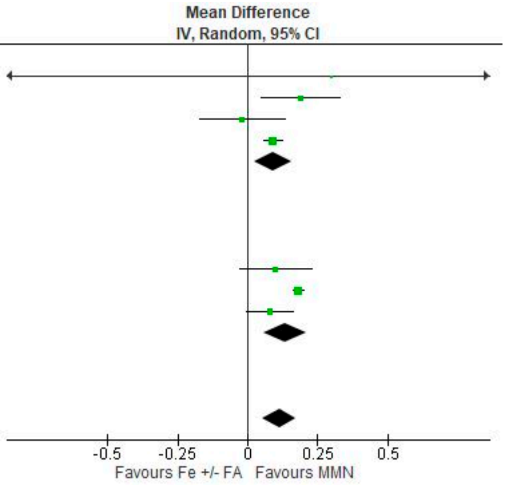

Footnotes

(1) *Values seem strange compared to other studies

(2) measured at 6 months postpartum

(3) Weekly MMN supplementation vs. daily IFA supplementation

(4) Assumed same sample sizes for intervention and control group as reported for serum hemoglobin

Maternal serum/plasma zinc concentration (umol/L)

Study or Subgroup	MMN Mean	MMN SD	MMN Total	Fe with or without FA Mean	Fe with or without FA SD	Fe with or without FA Total	Weight	Mean Difference IV, Random, 95% CI
**2.11.1 >4 micronutrients**								
Bhutta 2009	10.27	3.89	233	9.16	3.39	261	9.9%	1.11 [0.46, 1.76]
Tofail 2008	7.77	2.2897	212	7.54	2.8134	213	15.7%	0.23 [-0.26, 0.72]
West 2014	9.7	2.5	741	9.3	2.4	763	35.8%	0.40 [0.15, 0.65]
**Subtotal (95% CI)**			**1186**			**1237**	**61.5%**	**0.51 [0.11, 0.91]**
Heterogeneity: Tau² = 0.07; Chi² = 4.93, df = 2 (P = 0.08); I² = 59%								
Test for overall effect: Z = 2.49 (P = 0.01)								
**2.11.2 < or equal to 4 micronutrients**								
Caulfield 1999	8.6	1.5	270	8.3	1.4	268	36.2%	0.30 [0.05, 0.55]
Dijkhuizen 2001	9.1	2.9	34	9.1	3	33	2.4%	0.00 [-1.41, 1.41]
**Subtotal (95% CI)**			**304**			**301**	**38.5%**	**0.29 [0.05, 0.53]**
Heterogeneity: Tau² = 0.00; Chi² = 0.17, df = 1 (P = 0.68); I² = 0%								
Test for overall effect: Z = 2.36 (P = 0.02)								
**Total (95% CI)**			**1490**			**1538**	**100.0%**	**0.40 [0.18, 0.62]**
Heterogeneity: Tau² = 0.02; Chi² = 5.96, df = 4 (P = 0.20); I² = 33%								
Test for overall effect: Z = 3.52 (P = 0.0004)								
Test for subgroup differences: Chi² = 0.84, df = 1 (P = 0.36), I² = 0%								

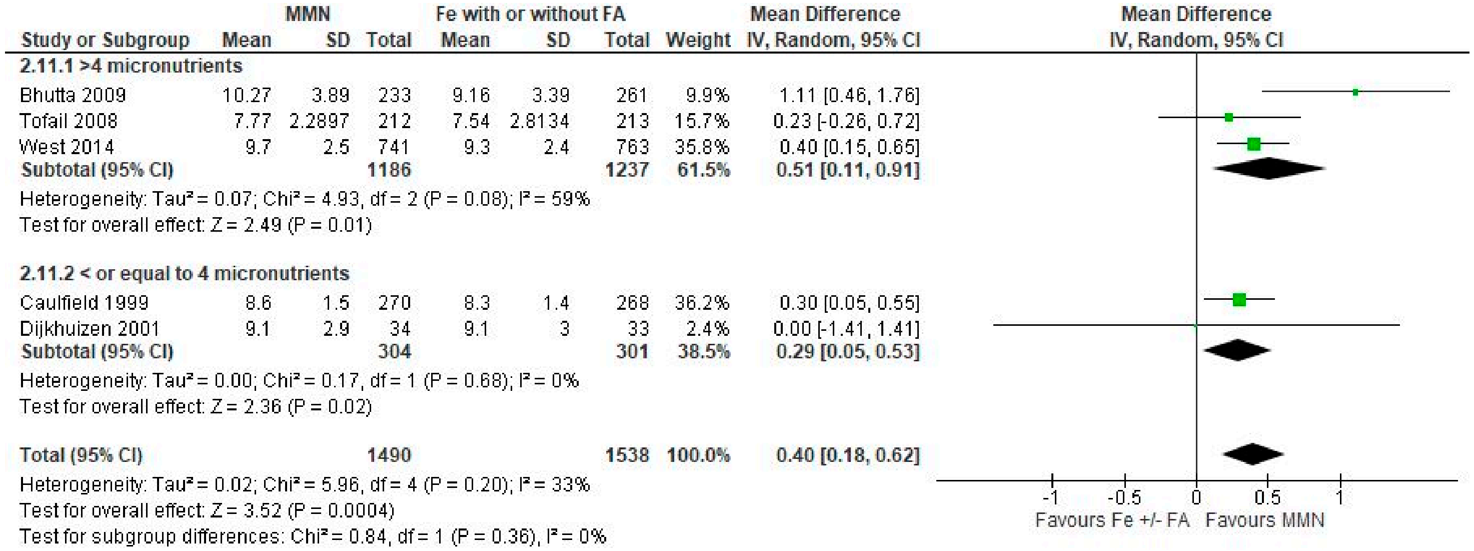

Supplementary Materials

Materna serum/plasma folate concentration (nmol/L)

Study or Subgroup	MMN Mean	MMN SD	MMN Total	Fe with or without FA Mean	Fe with or without FA SD	Fe with or without FA Total	Weight	Mean Difference IV, Random, 95% CI
**2.12.1 >4 micronutrients**								
Hanieh 2013	24.9	8.8	249	28.9	10.7	269	30.2%	-4.00 [-5.68, -2.32]
Ramakrishnan 2003	-38	205.5	63	-56.9	153.2	69	0.2%	18.90 [-43.40, 81.20]
Tofail 2008	21.8	11.0793	212	20.7	13.4215	213	27.3%	1.10 [-1.24, 3.44]
West 2014	27	20.3	746	30.6	19.9	774	28.7%	-3.60 [-5.62, -1.58]
**Subtotal (95% CI)**			**1270**			**1325**	**86.3%**	**-2.21 [-5.11, 0.69]**
Heterogeneity: Tau² = 5.53; Chi² = 13.65, df = 3 (P = 0.003); I² = 78%								
Test for overall effect: Z = 1.49 (P = 0.14)								
**2.12.2 < or equal to 4 micronutrients**								
Caulfield 1999	14.4	6.5	10	12.6	6.2	9	13.7%	1.80 [-3.91, 7.51]
**Subtotal (95% CI)**			**10**			**9**	**13.7%**	**1.80 [-3.91, 7.51]**
Heterogeneity: Not applicable								
Test for overall effect: Z = 0.62 (P = 0.54)								
**Total (95% CI)**			**1280**			**1334**	**100.0%**	**-1.66 [-4.39, 1.08]**
Heterogeneity: Tau² = 5.73; Chi² = 15.92, df = 4 (P = 0.003); I² = 75%								
Test for overall effect: Z = 1.19 (P = 0.24)								
Test for subgroup differences: Chi² = 1.50, df = 1 (P = 0.22), I² = 33.5%								

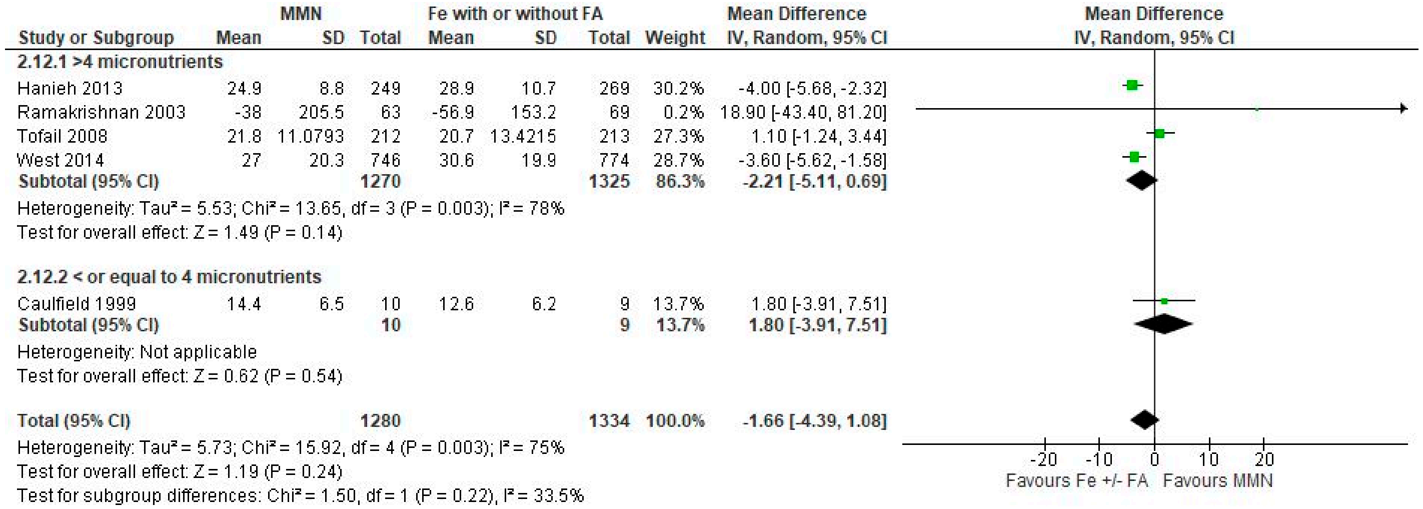

Maternal serum/plasma vitamin B12 concentration (pmol/L)

Study or Subgroup	MMN Mean	MMN SD	MMN Total	Fe with or without FA Mean	Fe with or without FA SD	Fe with or without FA Total	Weight	Mean Difference IV, Random, 95% CI
**2.13.1 >4 micronutrients**								
Hanieh 2013	249.2	90.3	249	239.4	78.1	269	43.7%	9.80 [-4.79, 24.39]
Tofail 2008	119	66.4759	212	101	74.5637	213	51.6%	18.00 [4.57, 31.43]
**Subtotal (95% CI)**			**461**			**482**	**95.3%**	**14.24 [4.36, 24.12]**
Heterogeneity: Tau² = 0.00; Chi² = 0.66, df = 1 (P = 0.42); I² = 0%								
Test for overall effect: Z = 2.82 (P = 0.005)								
**2.13.2 < or equal to 4 micronutrients**								
Caulfield 1999	101.9	65	10	76.4	28.4	9	4.7%	25.50 [-18.85, 69.85]
**Subtotal (95% CI)**			**10**			**9**	**4.7%**	**25.50 [-18.85, 69.85]**
Heterogeneity: Not applicable								
Test for overall effect: Z = 1.13 (P = 0.26)								
**Total (95% CI)**			**471**			**491**	**100.0%**	**14.77 [5.13, 24.42]**
Heterogeneity: Tau² = 0.00; Chi² = 0.89, df = 2 (P = 0.64); I² = 0%								
Test for overall effect: Z = 3.00 (P = 0.003)								
Test for subgroup differences: Chi² = 0.24, df = 1 (P = 0.63), I² = 0%								

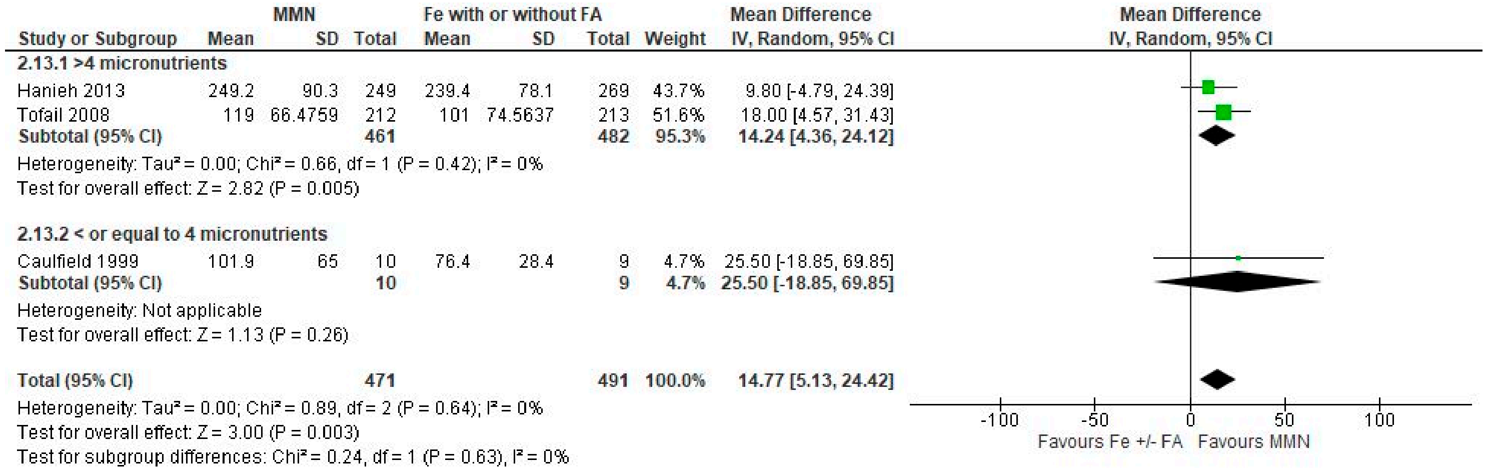

Supplementary Materials

Miscarriage

Study or Subgroup	log[Risk Ratio]	SE	MMN Total	Fe+/- FA Total	Weight	Risk Ratio IV, Random, 95% CI
Ashorn 2015 (1)	1.3886	1.116	433	434	0.1%	4.01 [0.45, 35.73]
Bhutta 2009	0.0488	0.1452	1148	1230	3.2%	1.05 [0.79, 1.40]
Dewey 2009 (2)	-0.3819	0.427	354	349	0.4%	0.68 [0.30, 1.58]
Fawzi 2007	-0.0408	0.199	4191	4188	1.7%	0.96 [0.65, 1.42]
Kæstel 2005	-0.0556	0.3814	695	708	0.5%	0.95 [0.45, 2.00]
Osrin 2005	-0.9163	0.8347	600	600	0.1%	0.40 [0.08, 2.05]
Roberfroid 2008	-0.0718	0.3678	632	628	0.5%	0.93 [0.45, 1.91]
SUMMIT 2008	-0.103	0.0889	12420	12194	8.5%	0.90 [0.76, 1.07]
Sunawang 2009	-0.5004	0.4775	360	343	0.3%	0.61 [0.24, 1.55]
Tofail 2008	0	0.1508	1480	1480	3.0%	1.00 [0.74, 1.34]
West 2014	-0.0017	0.0295	19483	19271	77.2%	1.00 [0.94, 1.06]
Zagré 2007	-0.0169	0.2126	1577	1481	1.5%	0.98 [0.65, 1.49]
Zeng 2008	0.1152	0.146	1345	1347	3.2%	1.12 [0.84, 1.49]
**Total (95% CI)**			**44718**	**44253**	**100.0%**	**0.99 [0.94, 1.04]**

Heterogeneity: Tau² = 0.00; Chi² = 6.71, df = 12 (P = 0.88); I² = 0%

Test for overall effect: Z = 0.36 (P = 0.72)

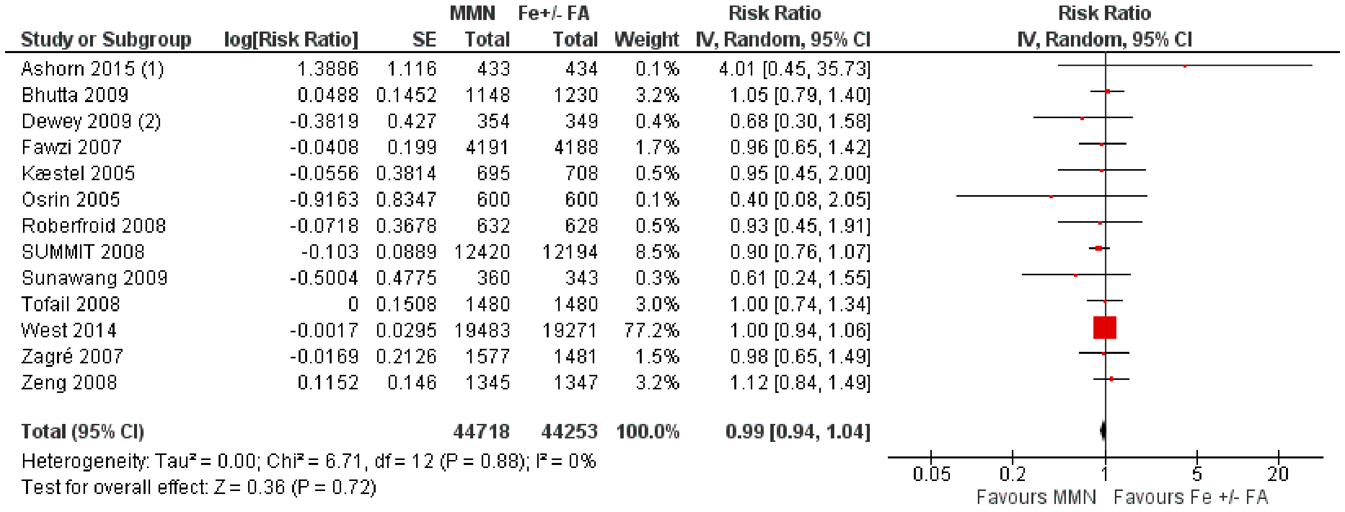

Footnotes

(1) Definition - loss of pregnancy up to 22 wks gestation

(2) In this paper: reference under Adu-Afarwuah et al. (2015)

Supplementary Materials

Congential Anomalies

Study or Subgroup	log[Risk Ratio]	SE	MMN Total	Fe +/- FA Total	Weight	Risk Ratio IV, Fixed, 95% CI
**2.16.1 >4 micronutrients**						
Bhutta 2009	-0.2937	0.9101	22	15	10.3%	0.75 [0.13, 4.44]
Dewey 2009 (1)	1.0913	1.6314	408	411	3.2%	2.98 [0.12, 72.88]
Osrin 2005	-0.0053	0.9982	571	568	8.5%	0.99 [0.14, 7.04]
**Subtotal (95% CI)**			**1001**	**994**	**22.0%**	**1.02 [0.30, 3.45]**
Heterogeneity: Chi² = 0.55, df = 2 (P = 0.76); I² = 0%						
Test for overall effect: Z = 0.03 (P = 0.98)						
**2.16.2 < or equal to 4 micronutrients**						
Christian 2003	1.4896	1.5482	725	644	3.5%	4.44 [0.21, 92.21]
Dijkhuizen 2001	0.5596	1.2064	48	42	5.8%	1.75 [0.16, 18.62]
Merialdi 2004	-0.3694	0.6314	109	113	21.3%	0.69 [0.20, 2.38]
Roth 2018 (MDIG)	-0.697	0.424	260	259	47.3%	0.50 [0.22, 1.14]
**Subtotal (95% CI)**			**1142**	**1058**	**78.0%**	**0.66 [0.35, 1.26]**
Heterogeneity: Chi² = 2.61, df = 3 (P = 0.46); I² = 0%						
Test for overall effect: Z = 1.25 (P = 0.21)						
**Total (95% CI)**			**2143**	**2052**	**100.0%**	**0.73 [0.41, 1.29]**
Heterogeneity: Chi² = 3.54, df = 6 (P = 0.74); I² = 0%						
Test for overall effect: Z = 1.09 (P = 0.27)						
Test for subgroup differences: Chi² = 0.38, df = 1 (P = 0.54), I² = 0%						

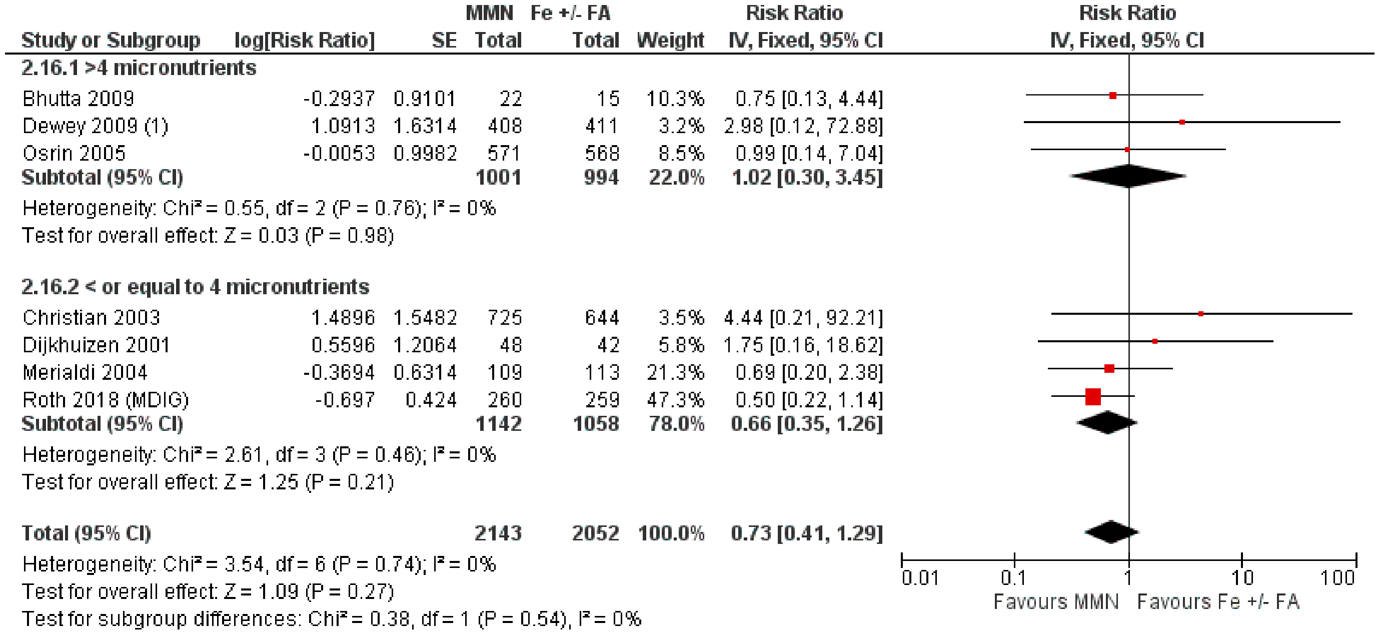

Footnotes

(1) in this paper, under reference: Adu-Afarwuah et al. (2015)

Supplementary Materials

Caesarean section as a mode of delivery

Study or Subgroup	log[Risk Ratio]	SE	MMN Total	Fe +/- FA Total	Weight	Risk Ratio IV, Random, 95% CI
**2.19.1 >4 micronutrients**						
Bhutta 2009	-0.0943	0.3825	743	832	0.7%	0.91 [0.43, 1.93]
Fawzi 2007	0.1398	0.0764	4079	4058	18.6%	1.15 [0.99, 1.34]
Friis 2004	-0.0408	0.4341	564	542	0.6%	0.96 [0.41, 2.25]
Osrin 2005	0.0621	0.2036	571	568	2.6%	1.06 [0.71, 1.59]
Roberfroid 2008	0.6868	0.8642	632	628	0.1%	1.99 [0.37, 10.81]
**Subtotal (95% CI)**			**6589**	**6628**	**22.6%**	**1.13 [0.99, 1.29]**
Heterogeneity: Tau² = 0.00; Chi² = 1.03, df = 4 (P = 0.91); I² = 0%						
Test for overall effect: Z = 1.76 (P = 0.08)						
**2.19.2 < or equal to 4 micronutrients**						
Asemi 2016	-0.3185	0.3474	21	21	0.9%	0.73 [0.37, 1.44]
Christian 2003	-0.0684	0.2778	726	625	1.4%	0.93 [0.54, 1.61]
Dijkhuizen 2001	0.968	1.6196	48	42	0.0%	2.63 [0.11, 62.95]
Roth 2013 (AViDD)	0.0136	0.1351	73	74	5.9%	1.01 [0.78, 1.32]
Roth 2018 (MDIG)	0.0445	0.0918	260	259	12.9%	1.05 [0.87, 1.25]
Villar 2006	-0.0503	0.0439	4181	4197	56.2%	0.95 [0.87, 1.04]
**Subtotal (95% CI)**			**5309**	**5218**	**77.4%**	**0.97 [0.90, 1.04]**
Heterogeneity: Tau² = 0.00; Chi² = 2.06, df = 5 (P = 0.84); I² = 0%						
Test for overall effect: Z = 0.87 (P = 0.38)						
**Total (95% CI)**			**11898**	**11846**	**100.0%**	**1.00 [0.94, 1.07]**
Heterogeneity: Tau² = 0.00; Chi² = 6.95, df = 10 (P = 0.73); I² = 0%						
Test for overall effect: Z = 0.07 (P = 0.94)						
Test for subgroup differences: Chi² = 3.86, df = 1 (P = 0.05), I² = 74.1%						

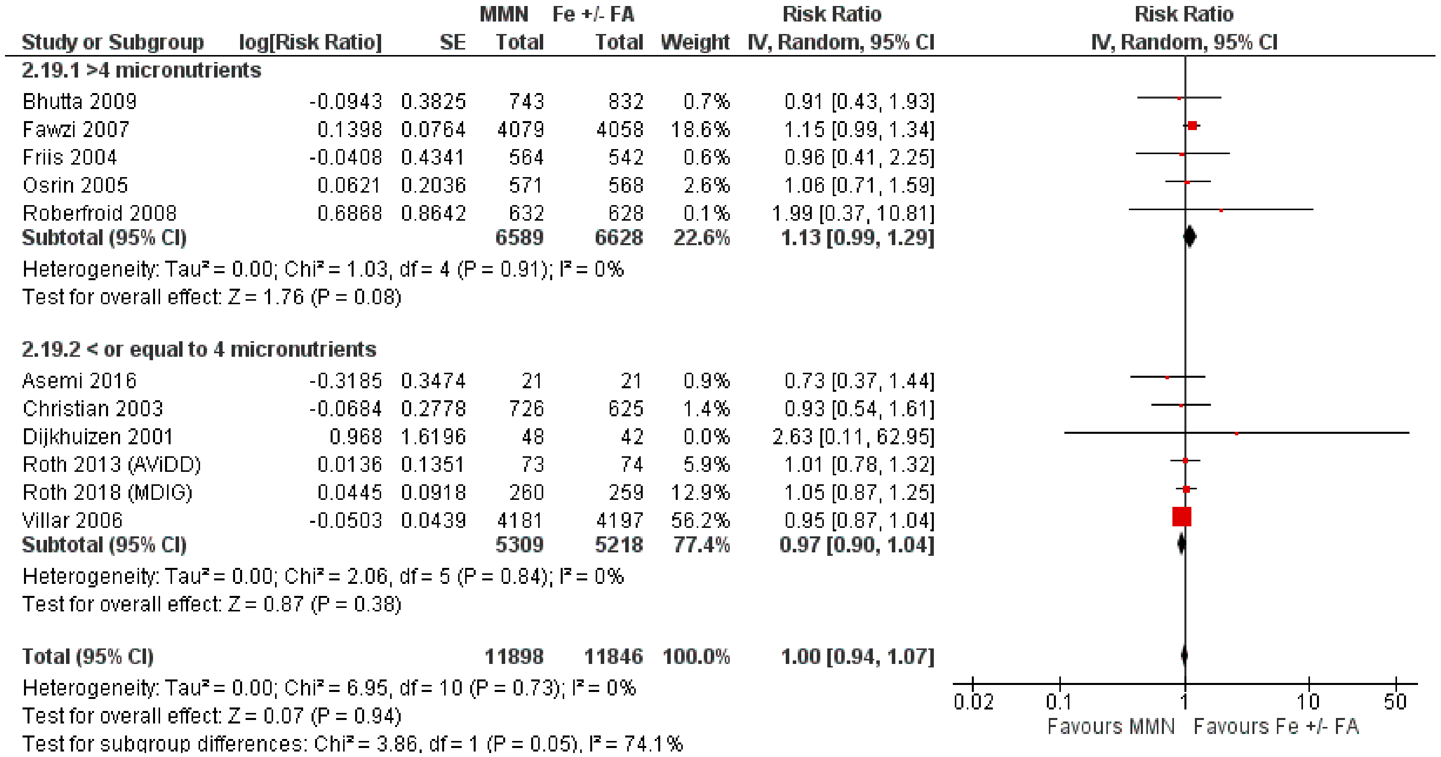

Supplementary Materials

Neonatal mortality

Study or Subgroup	log[Risk Ratio]	SE	Weight	Risk Ratio IV, Fixed, 95% CI
**2.20.1 >4 micronutrients**				
Ashorn 2015	-0.5113	0.3963	1.0%	0.60 [0.28, 1.30]
Bhutta 2009	0.3646	0.2122	3.5%	1.44 [0.95, 2.18]
Dewey 2009	0.6789	1.2225	0.1%	1.97 [0.18, 21.65]
Fawzi 2007	-0.1393	0.1333	8.8%	0.87 [0.67, 1.13]
Hanieh 2013 (1)	0.4358	0.9092	0.2%	1.55 [0.26, 9.19]
Kæstel 2005	0.1555	0.283	2.0%	1.17 [0.67, 2.03]
Liu 2013	-0.2107	0.2564	2.4%	0.81 [0.49, 1.34]
Osrin 2005	0.4245	0.3823	1.1%	1.53 [0.72, 3.23]
Roberfroid 2008	0.7028	0.4967	0.6%	2.02 [0.76, 5.35]
SUMMIT 2008	-0.1053	0.08626	21.1%	0.90 [0.76, 1.07]
Sunawang 2009	-0.6555	0.436	0.8%	0.52 [0.22, 1.22]
Tofail 2008	0.0191	0.2466	2.6%	1.02 [0.63, 1.65]
West 2014	-0.0202	0.0549	52.0%	0.98 [0.88, 1.09]
Zeng 2008	0.1743	0.3912	1.0%	1.19 [0.55, 2.56]
**Subtotal (95% CI)**			**97.2%**	**0.97 [0.89, 1.05]**
Heterogeneity: Chi² = 13.88, df = 13 (P = 0.38); I² = 6%				
Test for overall effect: Z = 0.82 (P = 0.41)				
**2.20.2 < or equal to 4 micronutrients**				
Christian 2003	0.3988	0.2405	2.7%	1.49 [0.93, 2.39]
Roth 2013 (AViDD)	-1.0848	1.1427	0.1%	0.34 [0.04, 3.17]
**Subtotal (95% CI)**			**2.8%**	**1.40 [0.88, 2.22]**
Heterogeneity: Chi² = 1.61, df = 1 (P = 0.20); I² = 38%				
Test for overall effect: Z = 1.43 (P = 0.15)				
**Total (95% CI)**			**100.0%**	**0.98 [0.90, 1.06]**
Heterogeneity: Chi² = 17.88, df = 15 (P = 0.27); I² = 16%				
Test for overall effect: Z = 0.57 (P = 0.57)				
Test for subgroup differences: Chi² = 2.39, df = 1 (P = 0.12), I² = 58.1%				

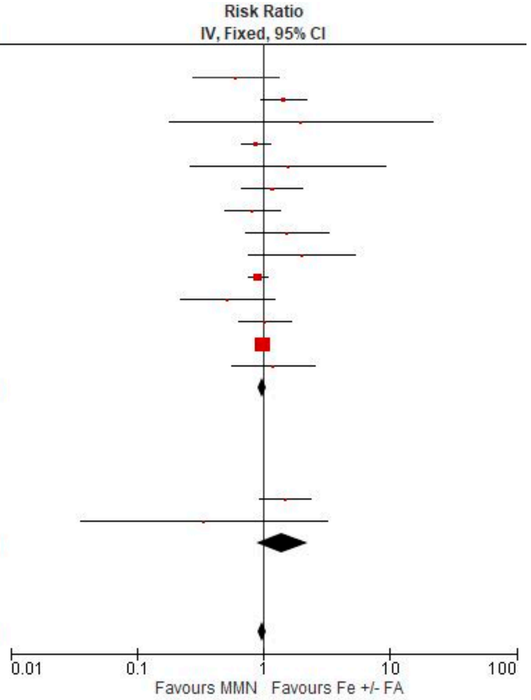

Footnotes

(1) infant deaths <24 days of life

Supplementary Materials

Infant mortality

Study or Subgroup	MMN Events	MMN Total	Fe +/- FA Events	Fe +/- FA Total	Weight	Risk Ratio M-H, Random, 95% CI
Ashorn 2015	17	290	11	288	1.2%	1.53 [0.73, 3.22]
Christian 2003 (1)	52	870	49	876	4.6%	1.07 [0.73, 1.56]
Fawzi 2007	95	3763	104	3724	8.8%	0.90 [0.69, 1.19]
Kæstel 2005	56	528	44	519	4.7%	1.25 [0.86, 1.82]
Liu 2013	45	5915	44	5926	3.9%	1.02 [0.68, 1.55]
Osrin 2005	24	556	21	550	2.0%	1.13 [0.64, 2.01]
Roberfroid 2008	44	650	36	644	3.7%	1.21 [0.79, 1.86]
Tofail 2008	28	740	21	741	2.2%	1.34 [0.77, 2.33]
West 2014 (2)	741	14374	764	14142	68.9%	0.95 [0.86, 1.05]
**Total (95% CI)**		**27686**		**27410**	**100.0%**	**0.99 [0.92, 1.08]**
Total events	1102		1094			

Heterogeneity: Tau² = 0.00; Chi² = 6.14, df = 8 (P = 0.63); I² = 0%

Test for overall effect: Z = 0.14 (P = 0.89)

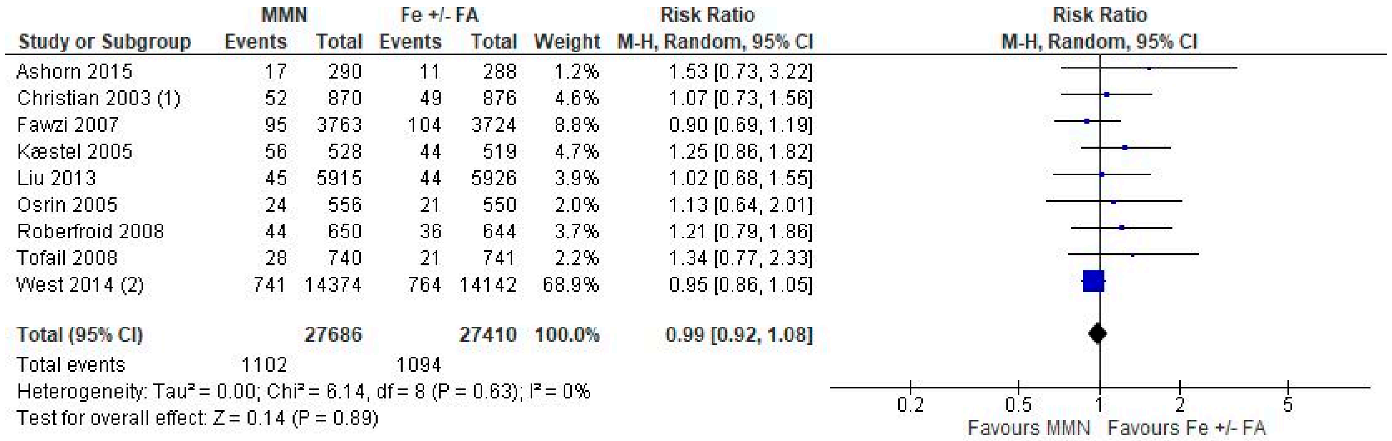

Footnotes

(1) *infant mortality defined as deaths at 3 months of age or less

(2) *infant mortality defined as deaths at 6 months of age or less

Child serum/plasma hemoglobin concentration (g/L)

Study or Subgroup	MMN Mean	MMN SD	MMN Total	Fe +/- FA Mean	Fe +/- FA SD	Fe +/- FA Total	Weight	Mean Difference IV, Random, 95% CI
**2.29.1 >4 micronutrients**								
Hanieh 2013	111.1	11.6	254	110.3	11.4	258	8.4%	0.80 [-1.19, 2.79]
Liu 2013 (1)	121.8	8.8	5734	121.7	8.6	5779	38.5%	0.10 [-0.22, 0.42]
SUMMIT 2008	110	13	174	109	13	76	3.1%	1.00 [-2.50, 4.50]
Tofail 2008	105.7	11.11	212	105.3	11.11	202	7.4%	0.40 [-1.74, 2.54]
**Subtotal (95% CI)**			**6374**			**6315**	**57.4%**	**0.13 [-0.18, 0.44]**
Heterogeneity: Tau² = 0.00; Chi² = 0.77, df = 3 (P = 0.86); I² = 0%								
Test for overall effect: Z = 0.82 (P = 0.41)								
**2.29.2 < or equal to 4 micronutrients**								
Caulfield 1999	122.2	10.3	79	120	9.7	86	4.0%	2.20 [-0.86, 5.26]
Dijkhuizen 2001	111	10	34	109	15	33	1.1%	2.00 [-4.12, 8.12]
Muslimatun 2001 (2)	105	1	72	105.7	1.2	74	37.5%	-0.70 [-1.06, -0.34]
**Subtotal (95% CI)**			**185**			**193**	**42.6%**	**0.42 [-1.81, 2.65]**
Heterogeneity: Tau² = 2.10; Chi² = 4.13, df = 2 (P = 0.13); I² = 52%								
Test for overall effect: Z = 0.37 (P = 0.71)								
**Total (95% CI)**			**6559**			**6508**	**100.0%**	**0.01 [-0.63, 0.66]**

Heterogeneity: Tau² = 0.25; Chi² = 15.49, df = 6 (P = 0.02); I² = 61%

Test for overall effect: Z = 0.04 (P = 0.97)

Test for subgroup differences: Chi² = 0.06, df = 1 (P = 0.80), I² = 0%

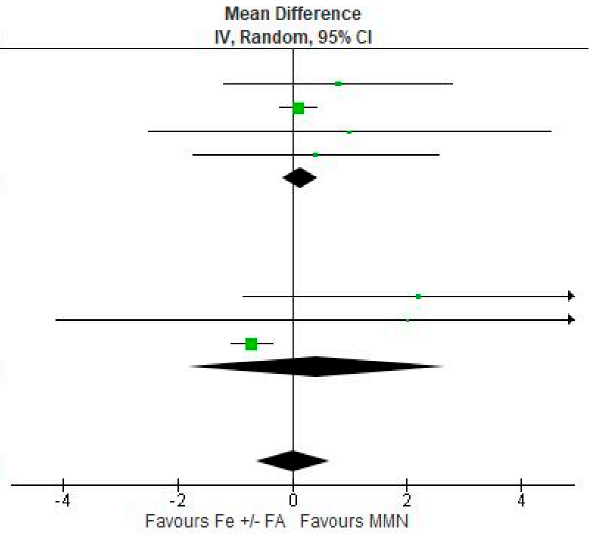

Footnotes

(1) Infant serum Hb measured at 6 months

(2) Weekly MMN supplementation vs. daily iron supplementation

Supplementary Materials

Child serum/plasma ferritin concentration (ug/L)

Study or Subgroup	MMN Mean	MMN SD	MMN Total	Fe +/- FA Mean	Fe +/- FA SD	Fe +/- FA Total	Weight	Mean Difference IV, Random, 95% CI
**2.30.1 >4 micronutrients**								
Hanieh 2013	28.8	25.2856	273	29.5	22.9513	270	42.7%	-0.70 [-4.76, 3.36]
Tofail 2008	27.9	26	360	24.1	22.29	340	55.0%	3.80 [0.22, 7.38]
**Subtotal (95% CI)**			**633**			**610**	**97.7%**	**1.66 [-2.75, 6.06]**
Heterogeneity: Tau² = 6.31; Chi² = 2.65, df = 1 (P = 0.10); I² = 62%								
Test for overall effect: Z = 0.74 (P = 0.46)								
**2.30.2 < or equal to 4 micronutrients**								
Dijkhuizen 2001	31.77	77.94	34	25.37	44.26	33	0.8%	6.40 [-23.84, 36.64]
Muslimatun 2001 (1)	74	66.8555	64	73.3	58.6947	69	1.5%	0.70 [-20.75, 22.15]
**Subtotal (95% CI)**			**98**			**102**	**2.3%**	**2.61 [-14.89, 20.10]**
Heterogeneity: Tau² = 0.00; Chi² = 0.09, df = 1 (P = 0.76); I² = 0%								
Test for overall effect: Z = 0.29 (P = 0.77)								
**Total (95% CI)**			**731**			**712**	**100.0%**	**1.85 [-0.81, 4.50]**
Heterogeneity: Tau² = 0.00; Chi² = 2.75, df = 3 (P = 0.43); I² = 0%								
Test for overall effect: Z = 1.37 (P = 0.17)								
Test for subgroup differences: Chi² = 0.01, df = 1 (P = 0.92), I² = 0%								

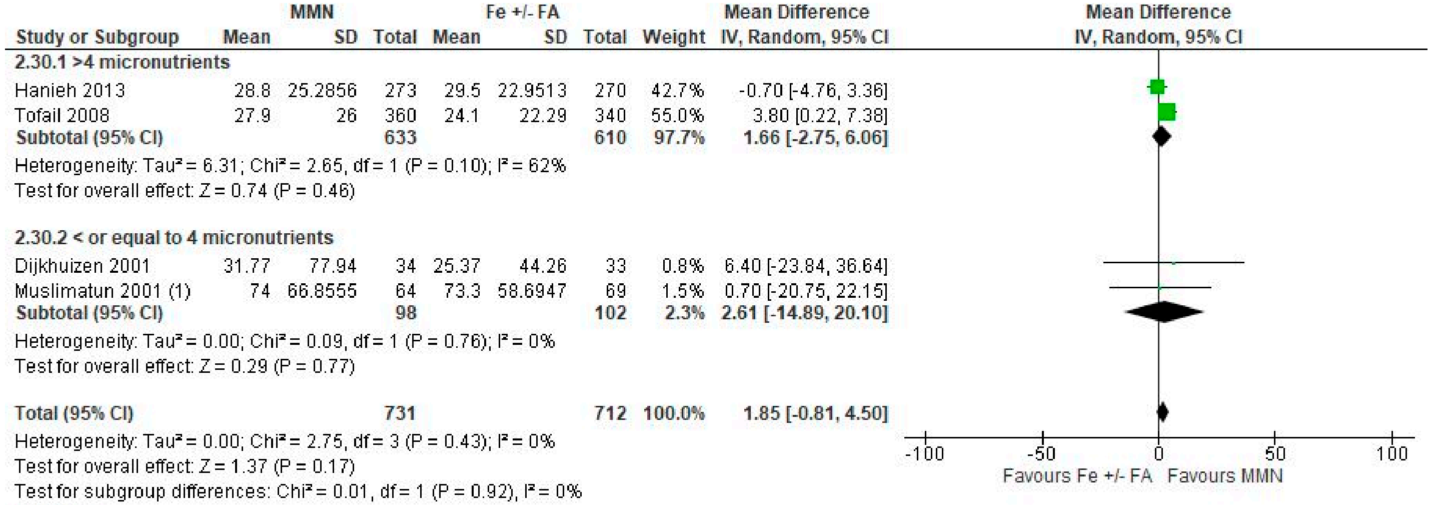

Footnotes
(1) Weekly MMN supplementation vs. daily iron supplementation

Child serum/plasma zinc concentration (umol/L)

Study or Subgroup	MMN Mean	MMN SD	MMN Total	Fe with or without FA Mean	Fe with or without FA SD	Fe with or without FA Total	Weight	Mean Difference IV, Random, 95% CI
**2.32.1 >4 micronutrients**								
Tofail 2008	9.5	1.926	357	9.5	2	355	79.8%	0.00 [-0.29, 0.29]
**Subtotal (95% CI)**			**357**			**355**	**79.8%**	**0.00 [-0.29, 0.29]**
Heterogeneity: Not applicable								
Test for overall effect: Z = 0.00 (P = 1.00)								
**2.32.2 < or equal to 4 micronutrients**								
Caulfield 1999	12.2	2.5	79	11.9	1.8	86	14.8%	0.30 [-0.37, 0.97]
Dijkhuizen 2001	12.4	2.5	34	12.4	2.1	33	5.4%	0.00 [-1.10, 1.10]
**Subtotal (95% CI)**			**113**			**119**	**20.2%**	**0.22 [-0.35, 0.79]**
Heterogeneity: Tau² = 0.00; Chi² = 0.21, df = 1 (P = 0.65); I² = 0%								
Test for overall effect: Z = 0.75 (P = 0.45)								
**Total (95% CI)**			**470**			**474**	**100.0%**	**0.04 [-0.21, 0.30]**
Heterogeneity: Tau² = 0.00; Chi² = 0.66, df = 2 (P = 0.72); I² = 0%								
Test for overall effect: Z = 0.34 (P = 0.74)								
Test for subgroup differences: Chi² = 0.45, df = 1 (P = 0.50), I² = 0%								

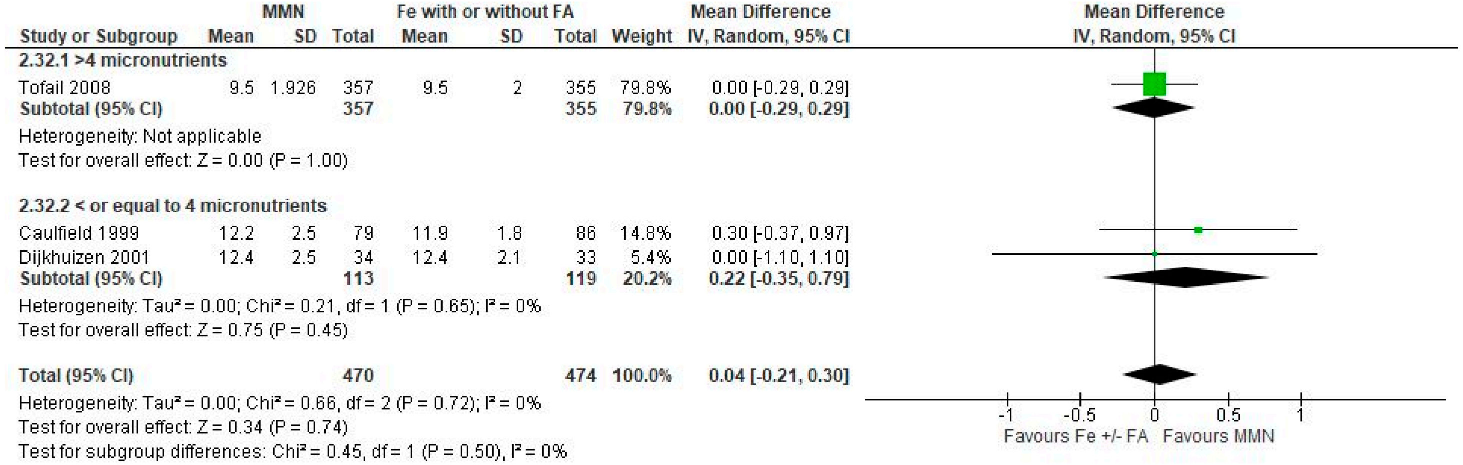

Supplementary Materials

Child anemia

Study or Subgroup	MMN Events	MMN Total	Fe +/- FA Events	Fe +/- FA Total	Weight	Risk Ratio M-H, Random, 95% CI
Hanieh 2013	128	254	122	258	34.6%	1.07 [0.89, 1.27]
Huy 2009	43	203	95	211	31.1%	0.47 [0.35, 0.64]
Tofail 2008	129	273	114	259	34.4%	1.07 [0.89, 1.29]
**Total (95% CI)**		**730**		**728**	**100.0%**	**0.83 [0.54, 1.28]**
Total events	300		331			

Heterogeneity: Tau² = 0.13; Chi² = 24.36, df = 2 (P < 0.00001); I² = 92%

Test for overall effect: Z = 0.85 (P = 0.39)

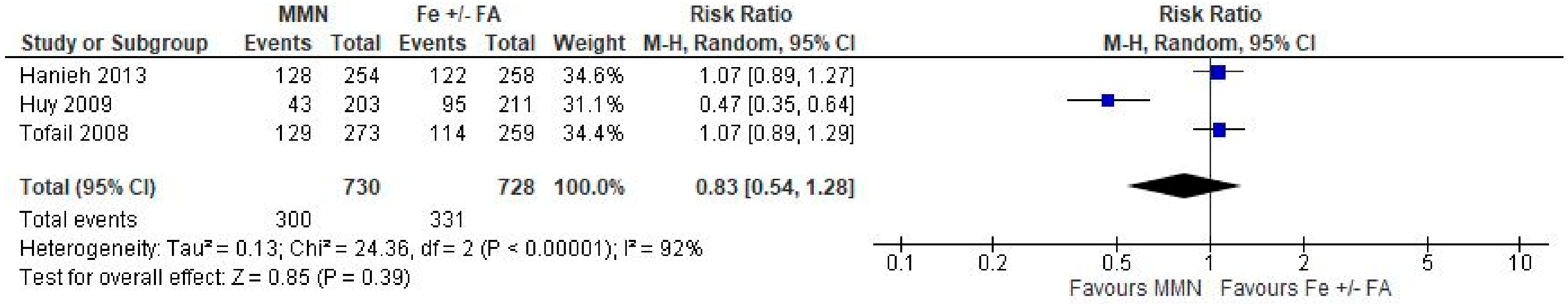

Supplementary Materials

Subgroup Analysis for Comparison: MMN Supplementation vs. IFA Supplementation

Maternal Mortality (MMN Formulation)

Study or Subgroup	log[Risk Ratio]	SE	Weight	Risk Ratio IV, Random, 95% CI
**9.1.1 UNIMMAP & Adapted UNIMMAP**				
Kæstel 2005	-0.5711	0.6236	9.4%	0.56 [0.17, 1.92]
SUMMIT 2008	0.02955	0.2427	62.1%	1.03 [0.64, 1.66]
West 2014 (1)	0.5768	0.5577	11.8%	1.78 [0.60, 5.31]
Zagré 2007	0.1906	0.7652	6.2%	1.21 [0.27, 5.42]
**Subtotal (95% CI)**			**89.5%**	**1.05 [0.71, 1.56]**
Heterogeneity: Tau² = 0.00; Chi² = 1.93, df = 3 (P = 0.59); I² = 0%				
Test for overall effect: Z = 0.25 (P = 0.81)				
**9.1.2 Non-UNIMMAP**				
Ashorn 2015	-0.3268	0.7603	6.3%	0.72 [0.16, 3.20]
Dewey 2009	1.1032	1.1527	2.8%	3.01 [0.31, 28.86]
Roth 2018 (MDIG)	-1.1025	1.6306	1.4%	0.33 [0.01, 8.11]
**Subtotal (95% CI)**			**10.5%**	**0.95 [0.30, 3.02]**
Heterogeneity: Tau² = 0.00; Chi² = 1.55, df = 2 (P = 0.46); I² = 0%				
Test for overall effect: Z = 0.09 (P = 0.93)				
**Total (95% CI)**			**100.0%**	**1.04 [0.71, 1.51]**
Heterogeneity: Tau² = 0.00; Chi² = 3.50, df = 6 (P = 0.74); I² = 0%				
Test for overall effect: Z = 0.20 (P = 0.84)				
Test for subgroup differences: Chi² = 0.03, df = 1 (P = 0.87), I² = 0%				

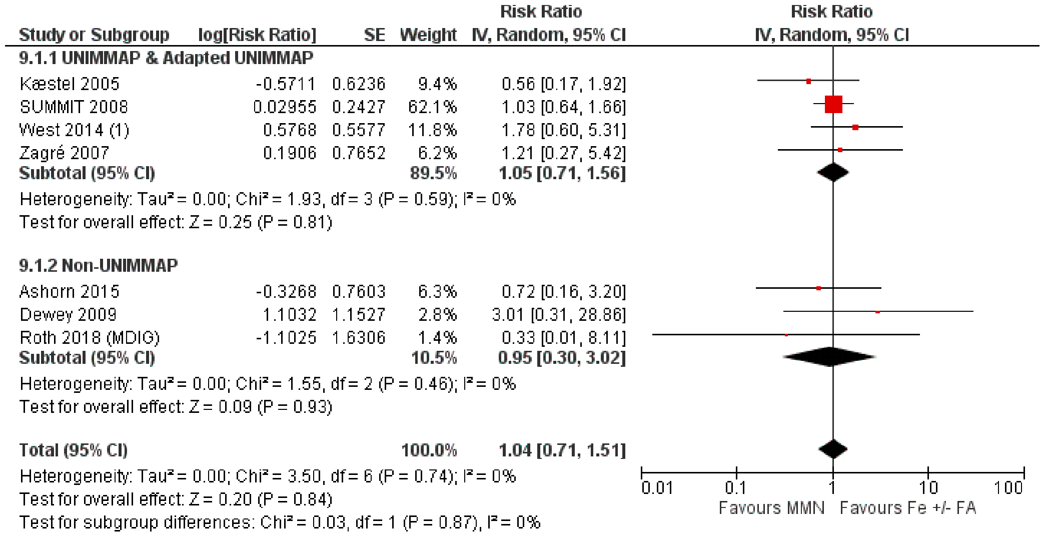

Footnotes

(1) Adapted UNIMMAP - same components, different doses per component

Maternal Mortality (Geographical Region)

Study or Subgroup	log[Risk Ratio]	SE	Weight	Risk Ratio IV, Random, 95% CI
**9.2.1 Africa**				
Ashorn 2015	-0.3268	0.7603	6.3%	0.72 [0.16, 3.20]
Dewey 2009	1.1032	1.1527	2.8%	3.01 [0.31, 28.86]
Kæstel 2005	-0.5711	0.6236	9.4%	0.56 [0.17, 1.92]
Zagré 2007	0.1906	0.7652	6.2%	1.21 [0.27, 5.42]
**Subtotal (95% CI)**			**24.7%**	**0.88 [0.41, 1.87]**
Heterogeneity: Tau² = 0.00; Chi² = 1.89, df = 3 (P = 0.60); I² = 0%				
Test for overall effect: Z = 0.34 (P = 0.74)				
**9.2.2 South Asia**				
Roth 2018 (MDIG)	-1.1025	1.6306	1.4%	0.33 [0.01, 8.11]
SUMMIT 2008	0.02955	0.2427	62.1%	1.03 [0.64, 1.66]
West 2014	0.5768	0.5577	11.8%	1.78 [0.60, 5.31]
**Subtotal (95% CI)**			**75.3%**	**1.10 [0.71, 1.69]**
Heterogeneity: Tau² = 0.00; Chi² = 1.36, df = 2 (P = 0.51); I² = 0%				
Test for overall effect: Z = 0.43 (P = 0.67)				
**Total (95% CI)**			**100.0%**	**1.04 [0.71, 1.51]**
Heterogeneity: Tau² = 0.00; Chi² = 3.50, df = 6 (P = 0.74); I² = 0%				
Test for overall effect: Z = 0.20 (P = 0.84)				
Test for subgroup differences: Chi² = 0.26, df = 1 (P = 0.61), I² = 0%				

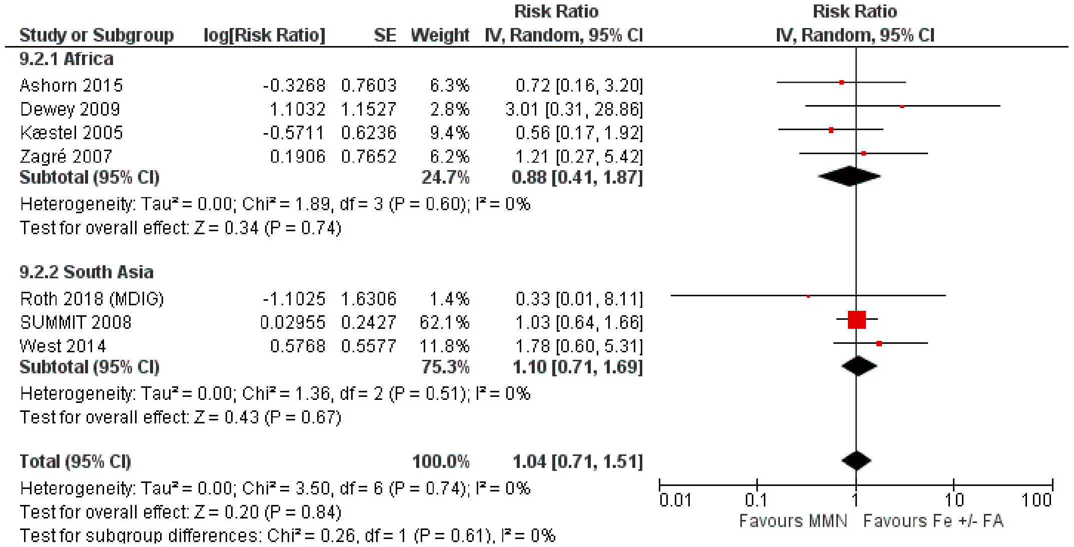

Supplementary Materials

Maternal Mortality (Duration of Intervention)

Study or Subgroup	log[Risk Ratio]	SE	Weight	Risk Ratio IV, Random, 95% CI
**9.3.1 Enrollment to Delivery**				
Kæstel 2005	-0.5711	0.6236	9.4%	0.56 [0.17, 1.92]
Roth 2018 (MDIG)	-1.1025	1.6306	1.4%	0.33 [0.01, 8.11]
West 2014	0.5768	0.5577	11.8%	1.78 [0.60, 5.31]
Zagré 2007	0.1906	0.7652	6.2%	1.21 [0.27, 5.42]
**Subtotal (95% CI)**			**28.8%**	**1.04 [0.52, 2.09]**
Heterogeneity: Tau² = 0.00; Chi² = 2.42, df = 3 (P = 0.49); I² = 0%				
Test for overall effect: Z = 0.11 (P = 0.92)				
**9.3.2 Enrollment to greater than or equal to 1 month postpartum**				
Ashorn 2015	-0.3268	0.7603	6.3%	0.72 [0.16, 3.20]
Dewey 2009	1.1032	1.1527	2.8%	3.01 [0.31, 28.86]
SUMMIT 2008	0.02955	0.2427	62.1%	1.03 [0.64, 1.66]
**Subtotal (95% CI)**			**71.2%**	**1.04 [0.67, 1.62]**
Heterogeneity: Tau² = 0.00; Chi² = 1.09, df = 2 (P = 0.58); I² = 0%				
Test for overall effect: Z = 0.17 (P = 0.86)				
**Total (95% CI)**			**100.0%**	**1.04 [0.71, 1.51]**
Heterogeneity: Tau² = 0.00; Chi² = 3.50, df = 6 (P = 0.74); I² = 0%				
Test for overall effect: Z = 0.20 (P = 0.84)				
Test for subgroup differences: Chi² = 0.00, df = 1 (P = 1.00), I² = 0%				

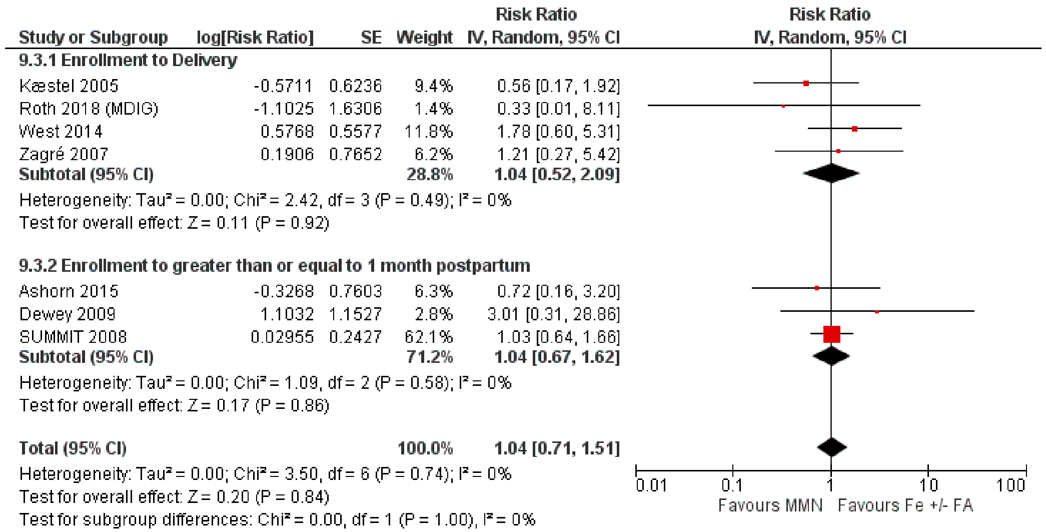

Maternal Mortality (Dose of Iron, mg)

Study or Subgroup	log[Risk Ratio]	SE	Weight	Risk Ratio IV, Random, 95% CI
**9.4.1 60mg of Iron**				
Ashorn 2015	-0.3268	0.7603	6.3%	0.72 [0.16, 3.20]
Dewey 2009	1.1032	1.1527	2.8%	3.01 [0.31, 28.86]
Roth 2018 (MDIG)	-1.1025	1.6306	1.4%	0.33 [0.01, 8.11]
Zagré 2007	0.1906	0.7652	6.2%	1.21 [0.27, 5.42]
**Subtotal (95% CI)**			**16.7%**	**1.04 [0.42, 2.60]**
Heterogeneity: Tau² = 0.00; Chi² = 1.61, df = 3 (P = 0.66); I² = 0%				
Test for overall effect: Z = 0.08 (P = 0.93)				
**9.4.2 <60mg of Iron**				
Kæstel 2005 (1)	-0.5711	0.6236	9.4%	0.56 [0.17, 1.92]
SUMMIT 2008 (2)	0.02955	0.2427	62.1%	1.03 [0.64, 1.66]
West 2014 (3)	0.5768	0.5577	11.8%	1.78 [0.60, 5.31]
**Subtotal (95% CI)**			**83.3%**	**1.04 [0.69, 1.57]**
Heterogeneity: Tau² = 0.00; Chi² = 1.89, df = 2 (P = 0.39); I² = 0%				
Test for overall effect: Z = 0.19 (P = 0.85)				
**Total (95% CI)**			**100.0%**	**1.04 [0.71, 1.51]**
Heterogeneity: Tau² = 0.00; Chi² = 3.50, df = 6 (P = 0.74); I² = 0%				
Test for overall effect: Z = 0.20 (P = 0.84)				
Test for subgroup differences: Chi² = 0.00, df = 1 (P = 1.00), I² = 0%				

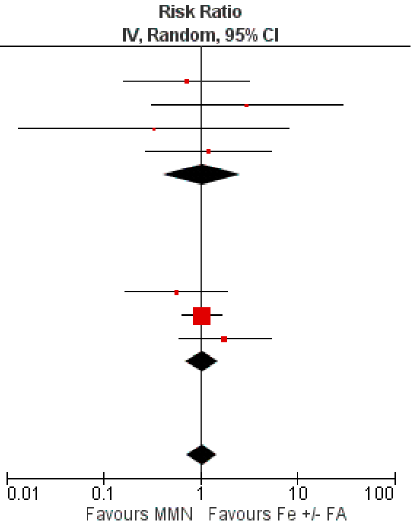

Footnotes
(1) 30mg Fe
(2) 30mg Fe
(3) 27mg Fe

Supplementary Materials

Maternal Anemia (MMN Formulation)

Study or Subgroup	log[Risk Ratio]	SE	Weight	Risk Ratio IV, Random, 95% CI
**9.5.1 UNIMMAP**				
Bhutta 2009	-0.1038	0.0969	7.7%	0.90 [0.75, 1.09]
Huy 2009	0.292	0.3564	1.1%	1.34 [0.67, 2.69]
Liu 2013	-0.0108	0.0763	9.4%	0.99 [0.85, 1.15]
Osrin 2005	-0.1373	0.1023	7.3%	0.87 [0.71, 1.07]
Roberfroid 2008	0.0327	0.0954	7.8%	1.03 [0.86, 1.25]
SUMMIT 2008	0.0236	0.0249	13.8%	1.02 [0.98, 1.08]
Zeng 2008	-0.0571	0.1319	5.5%	0.94 [0.73, 1.22]
**Subtotal (95% CI)**			**52.6%**	**1.01 [0.97, 1.05]**
Heterogeneity: Tau² = 0.00; Chi² = 4.74, df = 6 (P = 0.58); I² = 0%				
Test for overall effect: Z = 0.31 (P = 0.75)				
**9.5.2 Adapted UNIMMAP**				
Hanieh 2013	0.3454	0.2465	2.2%	1.41 [0.87, 2.29]
Moore 2012	-0.2076	0.2075	2.9%	0.81 [0.54, 1.22]
West 2014	0.0108	0.0623	10.7%	1.01 [0.89, 1.14]
**Subtotal (95% CI)**			**15.7%**	**1.02 [0.83, 1.25]**
Heterogeneity: Tau² = 0.01; Chi² = 2.95, df = 2 (P = 0.23); I² = 32%				
Test for overall effect: Z = 0.17 (P = 0.86)				
**9.5.3 Non-UNIMMAP**				
Ashorn 2015	0.1317	0.0789	9.2%	1.14 [0.98, 1.33]
Choudhury 2012	0.2099	0.1255	5.8%	1.23 [0.96, 1.58]
Christian 2003	0.3716	0.1999	3.0%	1.45 [0.98, 2.15]
Dewey 2009	0.5188	0.2161	2.7%	1.68 [1.10, 2.57]
Muslimatun 2001	-0.4701	0.1345	5.4%	0.62 [0.48, 0.81]
Ramakrishnan 2003	0.1178	0.1295	5.6%	1.13 [0.87, 1.45]
**Subtotal (95% CI)**			**31.7%**	**1.13 [0.89, 1.44]**
Heterogeneity: Tau² = 0.07; Chi² = 24.50, df = 5 (P = 0.0002); I² = 80%				
Test for overall effect: Z = 1.00 (P = 0.32)				
**Total (95% CI)**			**100.0%**	**1.02 [0.95, 1.10]**
Heterogeneity: Tau² = 0.01; Chi² = 34.65, df = 15 (P = 0.003); I² = 57%				
Test for overall effect: Z = 0.52 (P = 0.60)				
Test for subgroup differences: Chi² = 0.87, df = 2 (P = 0.65), I² = 0%				

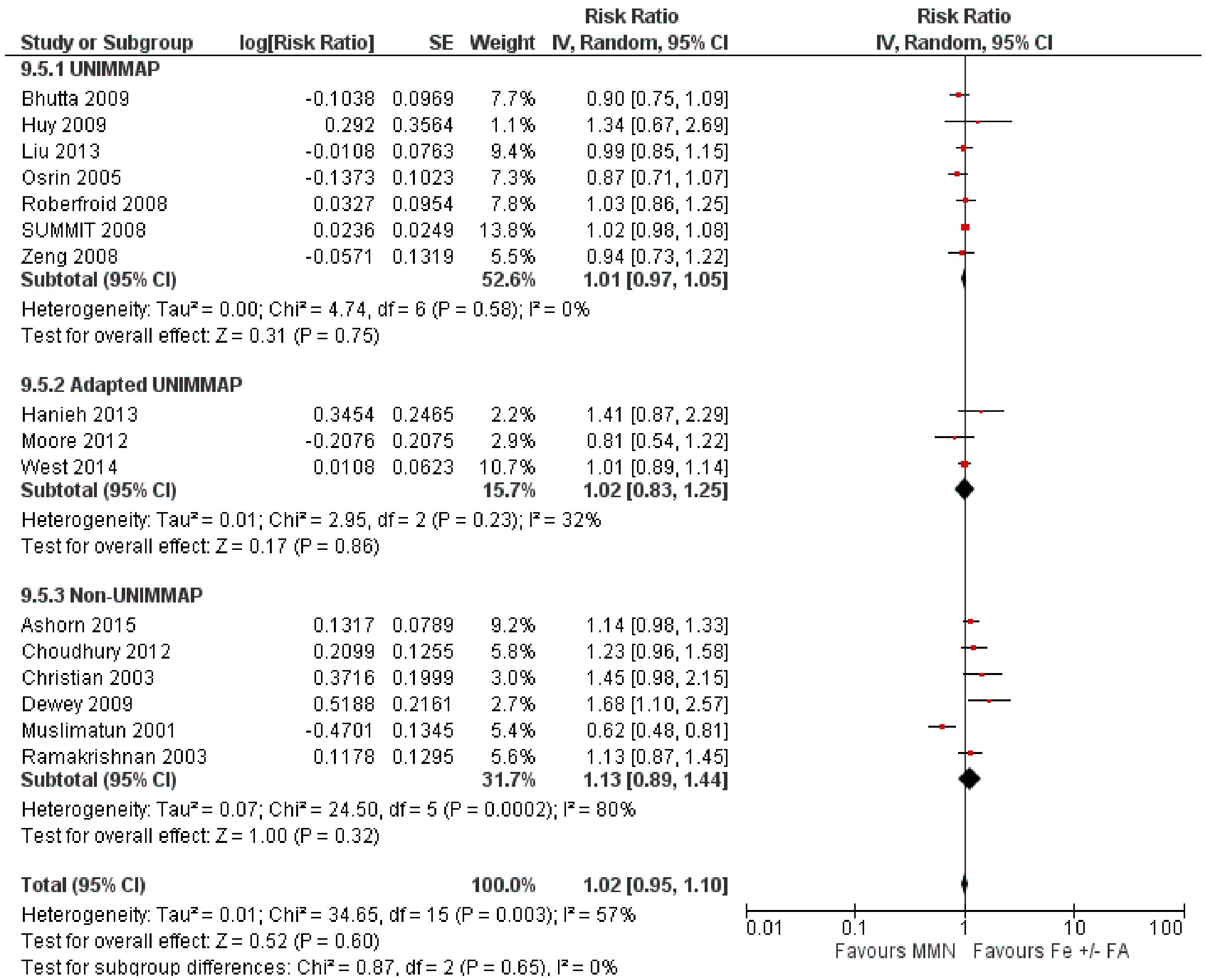

Supplementary Materials

Maternal Anemia (Geographical Region)

Study or Subgroup	log[Risk Ratio]	SE	Weight	Risk Ratio IV, Random, 95% CI
**9.6.1 Africa**				
Ashorn 2015	0.1317	0.0789	9.7%	1.14 [0.98, 1.33]
Dewey 2009	0.5188	0.2161	2.9%	1.68 [1.10, 2.57]
Moore 2012	-0.2076	0.2075	3.1%	0.81 [0.54, 1.22]
Roberfroid 2008	0.0327	0.0954	8.3%	1.03 [0.86, 1.25]
**Subtotal (95% CI)**			**24.0%**	**1.11 [0.92, 1.34]**
Heterogeneity: Tau² = 0.02; Chi² = 6.61, df = 3 (P = 0.09); I² = 55%				
Test for overall effect: Z = 1.06 (P = 0.29)				
**9.6.2 South Asia**				
Bhutta 2009	-0.1038	0.0969	8.2%	0.90 [0.75, 1.09]
Choudhury 2012	0.2099	0.1255	6.3%	1.23 [0.96, 1.58]
Christian 2003	0.3716	0.1999	3.3%	1.45 [0.98, 2.15]
Muslimatun 2001	-0.4701	0.1345	5.8%	0.62 [0.48, 0.81]
Osrin 2005	-0.1373	0.1023	7.8%	0.87 [0.71, 1.07]
SUMMIT 2008	0.0236	0.0249	14.3%	1.02 [0.98, 1.08]
West 2014	0.0108	0.0623	11.2%	1.01 [0.89, 1.14]
**Subtotal (95% CI)**			**56.7%**	**0.97 [0.86, 1.10]**
Heterogeneity: Tau² = 0.02; Chi² = 22.27, df = 6 (P = 0.001); I² = 73%				
Test for overall effect: Z = 0.48 (P = 0.63)				
**9.6.3 Western Pacific**				
Hanieh 2013	0.3454	0.2465	2.3%	1.41 [0.87, 2.29]
Huy 2009	0.292	0.3564	1.2%	1.34 [0.67, 2.69]
Liu 2013	-0.0108	0.0763	9.9%	0.99 [0.85, 1.15]
Zeng 2008	-0.0571	0.1319	5.9%	0.94 [0.73, 1.22]
**Subtotal (95% CI)**			**19.3%**	**1.01 [0.89, 1.14]**
Heterogeneity: Tau² = 0.00; Chi² = 2.81, df = 3 (P = 0.42); I² = 0%				
Test for overall effect: Z = 0.18 (P = 0.86)				
**Total (95% CI)**			**100.0%**	**1.02 [0.94, 1.10]**
Heterogeneity: Tau² = 0.01; Chi² = 34.06, df = 14 (P = 0.002); I² = 59%				
Test for overall effect: Z = 0.37 (P = 0.71)				
Test for subgroup differences: Chi² = 1.33, df = 2 (P = 0.51), I² = 0%				

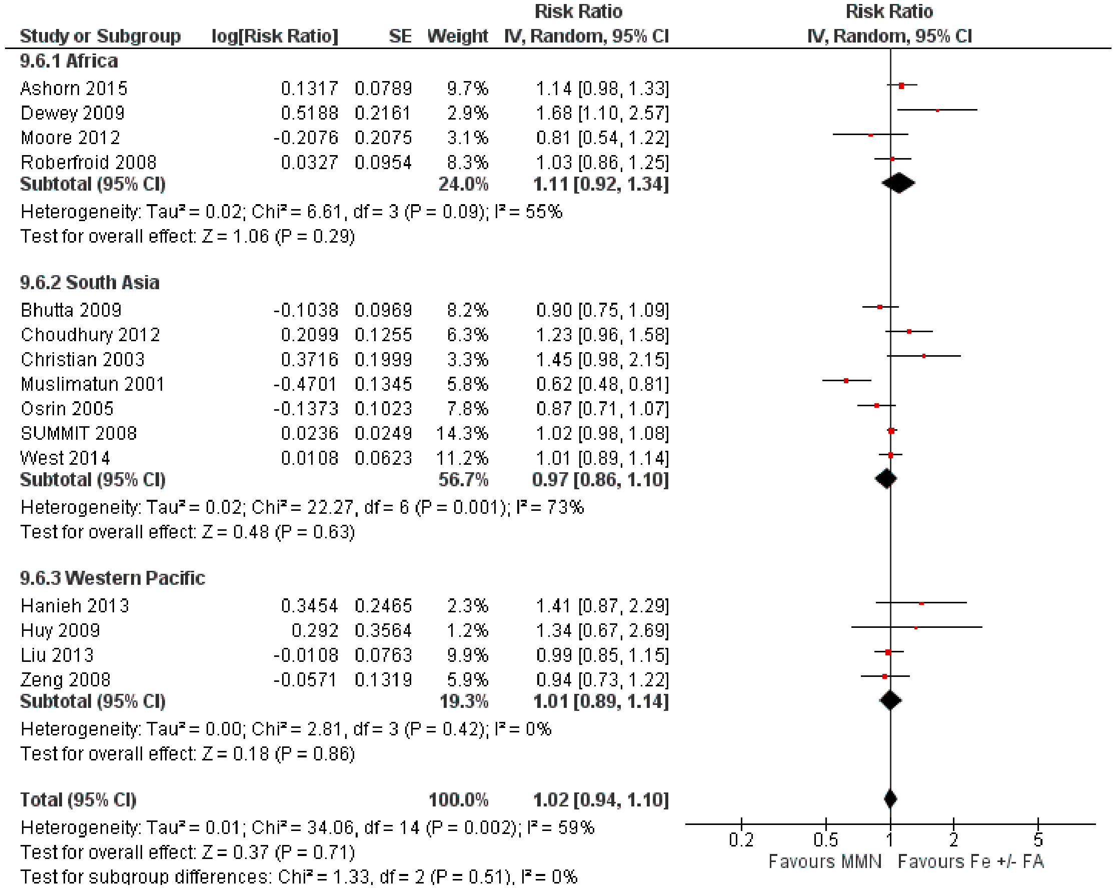

Supplementary Materials

Maternal Anemia (Dose of Iron, mg)

Study or Subgroup	log[Risk Ratio]	SE	Weight	Risk Ratio IV, Random, 95% CI
**9.7.1 60mg of Iron**				
Choudhury 2012	0.2099	0.1255	5.8%	1.23 [0.96, 1.58]
Christian 2003	0.3716	0.1999	3.0%	1.45 [0.98, 2.15]
Hanieh 2013	0.3454	0.2465	2.2%	1.41 [0.87, 2.29]
Moore 2012	-0.2076	0.2075	2.9%	0.81 [0.54, 1.22]
Muslimatun 2001	-0.4701	0.1345	5.4%	0.62 [0.48, 0.81]
Ramakrishnan 2003	0.1178	0.1295	5.6%	1.13 [0.87, 1.45]
**Subtotal (95% CI)**			**24.9%**	**1.05 [0.79, 1.38]**
Heterogeneity: Tau² = 0.09; Chi² = 22.18, df = 5 (P = 0.0005); I² = 77%				
Test for overall effect: Z = 0.33 (P = 0.74)				
**9.7.2 <60mg of Iron**				
Ashorn 2015	0.1317	0.0789	9.2%	1.14 [0.98, 1.33]
Bhutta 2009	-0.1038	0.0969	7.7%	0.90 [0.75, 1.09]
Dewey 2009	0.5188	0.2161	2.7%	1.68 [1.10, 2.57]
Huy 2009	0.292	0.3564	1.1%	1.34 [0.67, 2.69]
Liu 2013 (1)	-0.0108	0.0763	9.4%	0.99 [0.85, 1.15]
Osrin 2005	-0.1373	0.1023	7.3%	0.87 [0.71, 1.07]
Roberfroid 2008	0.0327	0.0954	7.8%	1.03 [0.86, 1.25]
SUMMIT 2008 (2)	0.0236	0.0249	13.8%	1.02 [0.98, 1.08]
West 2014 (3)	0.0108	0.0623	10.7%	1.01 [0.89, 1.14]
Zeng 2008	-0.0571	0.1319	5.5%	0.94 [0.73, 1.22]
**Subtotal (95% CI)**			**75.1%**	**1.02 [0.96, 1.08]**
Heterogeneity: Tau² = 0.00; Chi² = 12.47, df = 9 (P = 0.19); I² = 28%				
Test for overall effect: Z = 0.52 (P = 0.60)				
**Total (95% CI)**			**100.0%**	**1.02 [0.95, 1.10]**
Heterogeneity: Tau² = 0.01; Chi² = 34.65, df = 15 (P = 0.003); I² = 57%				
Test for overall effect: Z = 0.52 (P = 0.60)				
Test for subgroup differences: Chi² = 0.05, df = 1 (P = 0.83), I² = 0%				

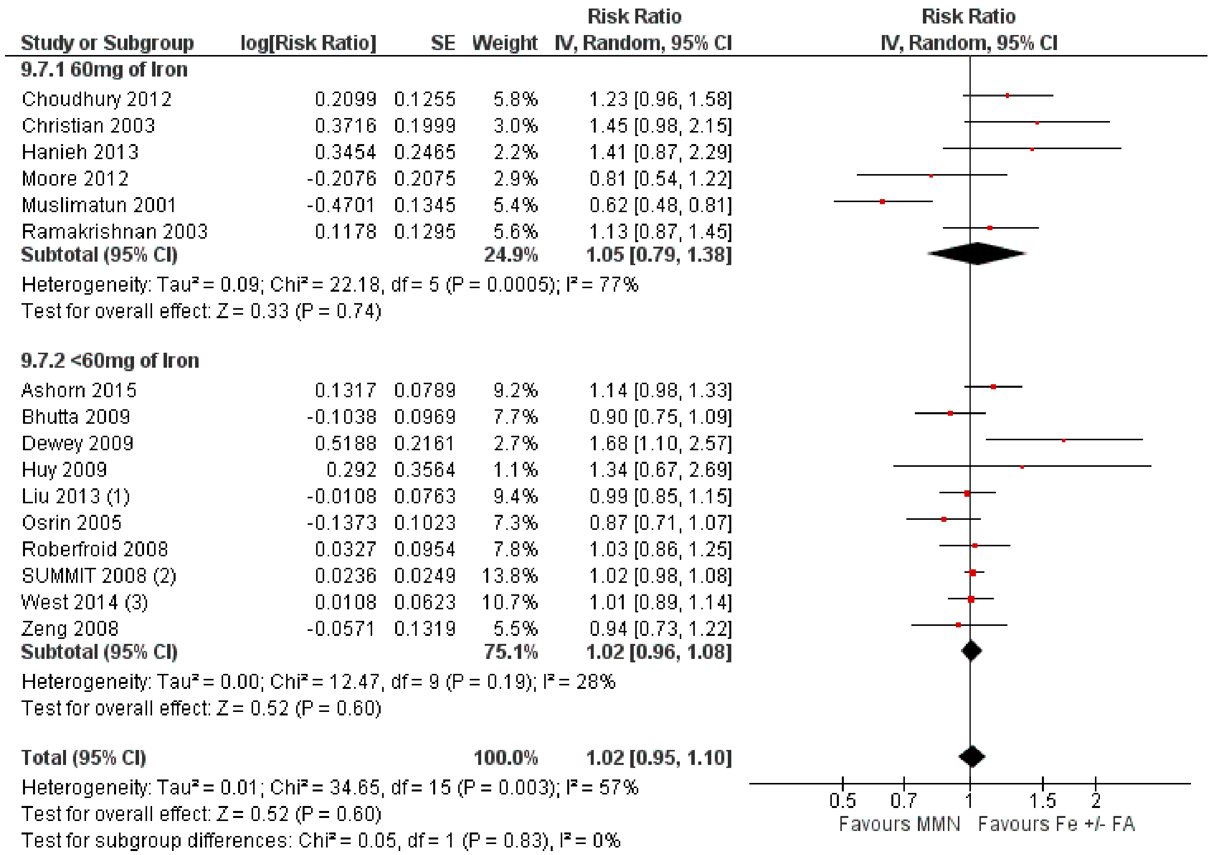

Footnotes

(1) 30mg of Fe

(2) 30mg of Fe

(3) 27mg of Fe

Supplementary Materials

Perinatal Mortality (MMN Formulation)

Study or Subgroup	log[Risk Ratio]	SE	Weight	Risk Ratio IV, Random, 95% CI
**9.8.1 UNIMMAP & Adapted UNIMMAP**				
Bhutta 2009	0.2927	0.1545	7.9%	1.34 [0.99, 1.81]
Kæstel 2005	-0.1884	0.2044	5.4%	0.83 [0.55, 1.24]
Liu 2013	-0.0619	0.1961	5.7%	0.94 [0.64, 1.38]
Osrin 2005	0.1914	0.2751	3.3%	1.21 [0.71, 2.08]
Roberfroid 2008	0.7245	0.3329	2.4%	2.06 [1.07, 3.96]
SUMMIT 2008	-0.10536	0.06651	16.5%	0.90 [0.79, 1.03]
Sunawang 2009	-0.1406	0.281	3.2%	0.87 [0.50, 1.51]
Tofail 2008	-0.1195	0.1834	6.3%	0.89 [0.62, 1.27]
West 2014 (1)	-0.0726	0.0399	19.6%	0.93 [0.86, 1.01]
Zeng 2008	0.3195	0.1942	5.8%	1.38 [0.94, 2.01]
**Subtotal (95% CI)**			**75.9%**	**1.01 [0.89, 1.14]**
Heterogeneity: Tau² = 0.01; Chi² = 16.45, df = 9 (P = 0.06); I² = 45%				
Test for overall effect: Z = 0.13 (P = 0.89)				
**9.8.2 Non-UNIMMAP**				
Ashorn 2015	-0.5793	0.3715	2.0%	0.56 [0.27, 1.16]
Christian 2003	0.3436	0.1856	6.2%	1.41 [0.98, 2.03]
Dewey 2009	-0.2374	0.4683	1.3%	0.79 [0.31, 1.97]
Dijkhuizen 2001	2.0666	1.476	0.1%	7.90 [0.44, 142.52]
Fawzi 2007	-0.1278	0.0884	13.9%	0.88 [0.74, 1.05]
Ramakrishnan 2003	0.2151	0.6601	0.7%	1.24 [0.34, 4.52]
**Subtotal (95% CI)**			**24.1%**	**0.98 [0.70, 1.37]**
Heterogeneity: Tau² = 0.07; Chi² = 9.66, df = 5 (P = 0.09); I² = 48%				
Test for overall effect: Z = 0.11 (P = 0.91)				
**Total (95% CI)**			**100.0%**	**1.00 [0.90, 1.11]**
Heterogeneity: Tau² = 0.01; Chi² = 26.13, df = 15 (P = 0.04); I² = 43%				
Test for overall effect: Z = 0.03 (P = 0.98)				
Test for subgroup differences: Chi² = 0.02, df = 1 (P = 0.88), I² = 0%				

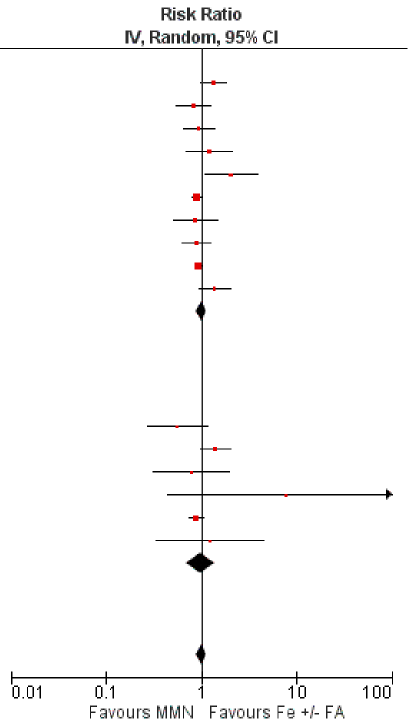

Footnotes

(1) Adapted UNIMMAP - same components, different doses per component

Supplementary Materials

Perinatal Mortality (Geographical Region)

Study or Subgroup	log[Risk Ratio]	SE	Weight	Risk Ratio IV, Random, 95% CI
**9.9.1 Africa**				
Ashorn 2015	-0.5793	0.3715	2.0%	0.56 [0.27, 1.16]
Dewey 2009	-0.2374	0.4683	1.3%	0.79 [0.31, 1.97]
Fawzi 2007	-0.1278	0.0884	13.8%	0.88 [0.74, 1.05]
Kæstel 2005	-0.1884	0.2044	5.5%	0.83 [0.55, 1.24]
Roberfroid 2008	0.7245	0.3329	2.5%	2.06 [1.07, 3.96]
**Subtotal (95% CI)**			**25.1%**	**0.92 [0.68, 1.25]**
Heterogeneity: Tau² = 0.06; Chi² = 8.14, df = 4 (P = 0.09); I² = 51%				
Test for overall effect: Z = 0.54 (P = 0.59)				
**9.9.2 Western Pacific**				
Liu 2013	-0.0619	0.1961	5.8%	0.94 [0.64, 1.38]
Zeng 2008	0.3195	0.1942	5.9%	1.38 [0.94, 2.01]
**Subtotal (95% CI)**			**11.8%**	**1.14 [0.78, 1.65]**
Heterogeneity: Tau² = 0.03; Chi² = 1.91, df = 1 (P = 0.17); I² = 48%				
Test for overall effect: Z = 0.68 (P = 0.50)				
**9.9.3 South Asia**				
Bhutta 2009	0.2927	0.1545	8.1%	1.34 [0.99, 1.81]
Christian 2003	0.3436	0.1856	6.3%	1.41 [0.98, 2.03]
Dijkhuizen 2001	2.0666	1.476	0.1%	7.90 [0.44, 142.52]
Osrin 2005	0.1914	0.2751	3.4%	1.21 [0.71, 2.08]
SUMMIT 2008	-0.10536	0.06651	16.3%	0.90 [0.79, 1.03]
Sunawang 2009	-0.1406	0.281	3.3%	0.87 [0.50, 1.51]
Tofail 2008	-0.1195	0.1834	6.4%	0.89 [0.62, 1.27]
West 2014	-0.0726	0.0399	19.1%	0.93 [0.86, 1.01]
**Subtotal (95% CI)**			**63.1%**	**1.01 [0.89, 1.16]**
Heterogeneity: Tau² = 0.01; Chi² = 13.50, df = 7 (P = 0.06); I² = 48%				
Test for overall effect: Z = 0.20 (P = 0.84)				
**Total (95% CI)**			**100.0%**	**1.00 [0.90, 1.12]**
Heterogeneity: Tau² = 0.01; Chi² = 25.96, df = 14 (P = 0.03); I² = 46%				
Test for overall effect: Z = 0.03 (P = 0.98)				
Test for subgroup differences: Chi² = 0.76, df = 2 (P = 0.68), I² = 0%				

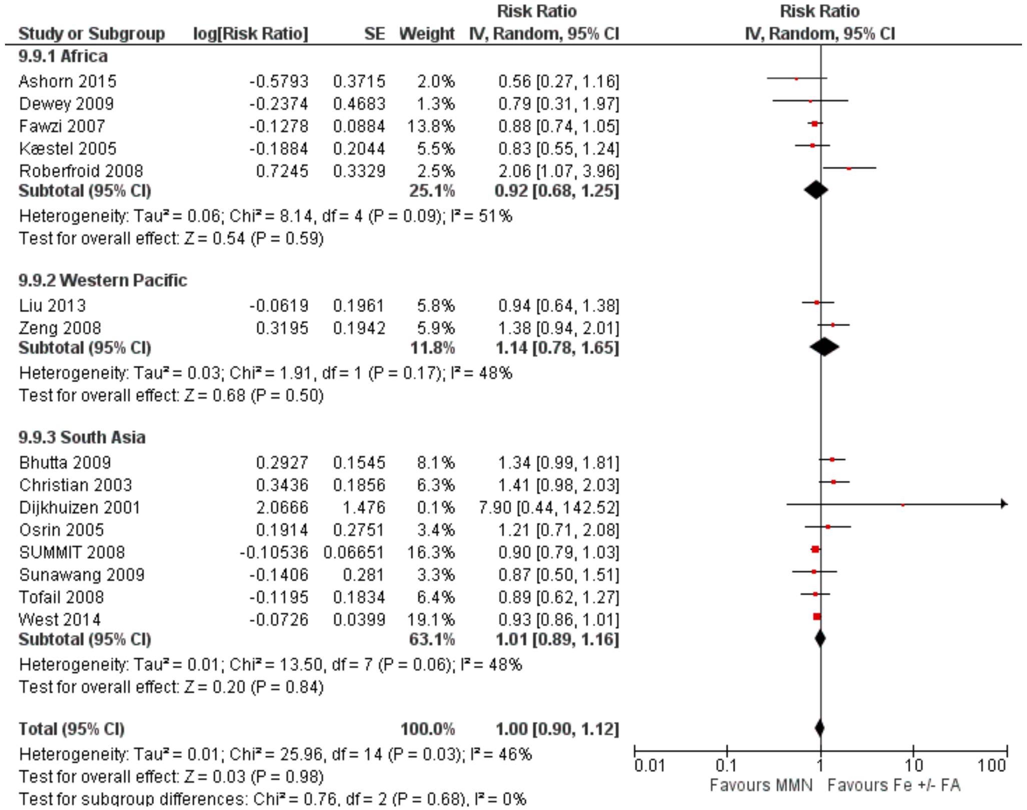

Supplementary Materials

Perinatal Mortality (Dose of Iron, mg)

Study or Subgroup	log[Risk Ratio]	SE	Weight	Risk Ratio IV, Random, 95% CI
**9.10.1 60mg of Iron**				
Christian 2003	0.3436	0.1856	6.2%	1.41 [0.98, 2.03]
Fawzi 2007	-0.1278	0.0884	13.9%	0.88 [0.74, 1.05]
Ramakrishnan 2003	0.2151	0.6601	0.7%	1.24 [0.34, 4.52]
**Subtotal (95% CI)**			**20.7%**	**1.09 [0.74, 1.60]**
Heterogeneity: Tau² = 0.07; Chi² = 5.41, df = 2 (P = 0.07); I² = 63%				
Test for overall effect: Z = 0.42 (P = 0.67)				
**9.10.2 <60mg of Iron**				
Ashorn 2015	-0.5793	0.3715	2.0%	0.56 [0.27, 1.16]
Bhutta 2009	0.2927	0.1545	7.9%	1.34 [0.99, 1.81]
Dewey 2009	-0.2374	0.4683	1.3%	0.79 [0.31, 1.97]
Dijkhuizen 2001	2.0666	1.476	0.1%	7.90 [0.44, 142.52]
Kæstel 2005 (1)	-0.1884	0.2044	5.4%	0.83 [0.55, 1.24]
Liu 2013 (2)	-0.0619	0.1961	5.7%	0.94 [0.64, 1.38]
Osrin 2005	0.1914	0.2751	3.3%	1.21 [0.71, 2.08]
Roberfroid 2008	0.7245	0.3329	2.4%	2.06 [1.07, 3.96]
SUMMIT 2008 (3)	-0.10536	0.06651	16.5%	0.90 [0.79, 1.03]
Sunawang 2009	-0.1406	0.281	3.2%	0.87 [0.50, 1.51]
Tofail 2008	-0.1195	0.1834	6.3%	0.89 [0.62, 1.27]
West 2014 (4)	-0.0726	0.0399	19.6%	0.93 [0.86, 1.01]
Zeng 2008	0.3195	0.1942	5.8%	1.38 [0.94, 2.01]
**Subtotal (95% CI)**			**79.3%**	**1.00 [0.88, 1.12]**
Heterogeneity: Tau² = 0.01; Chi² = 20.68, df = 12 (P = 0.06); I² = 42%				
Test for overall effect: Z = 0.06 (P = 0.95)				
**Total (95% CI)**			**100.0%**	**1.00 [0.90, 1.11]**
Heterogeneity: Tau² = 0.01; Chi² = 26.13, df = 15 (P = 0.04); I² = 43%				
Test for overall effect: Z = 0.03 (P = 0.98)				
Test for subgroup differences: Chi² = 0.18, df = 1 (P = 0.68), I² = 0%				

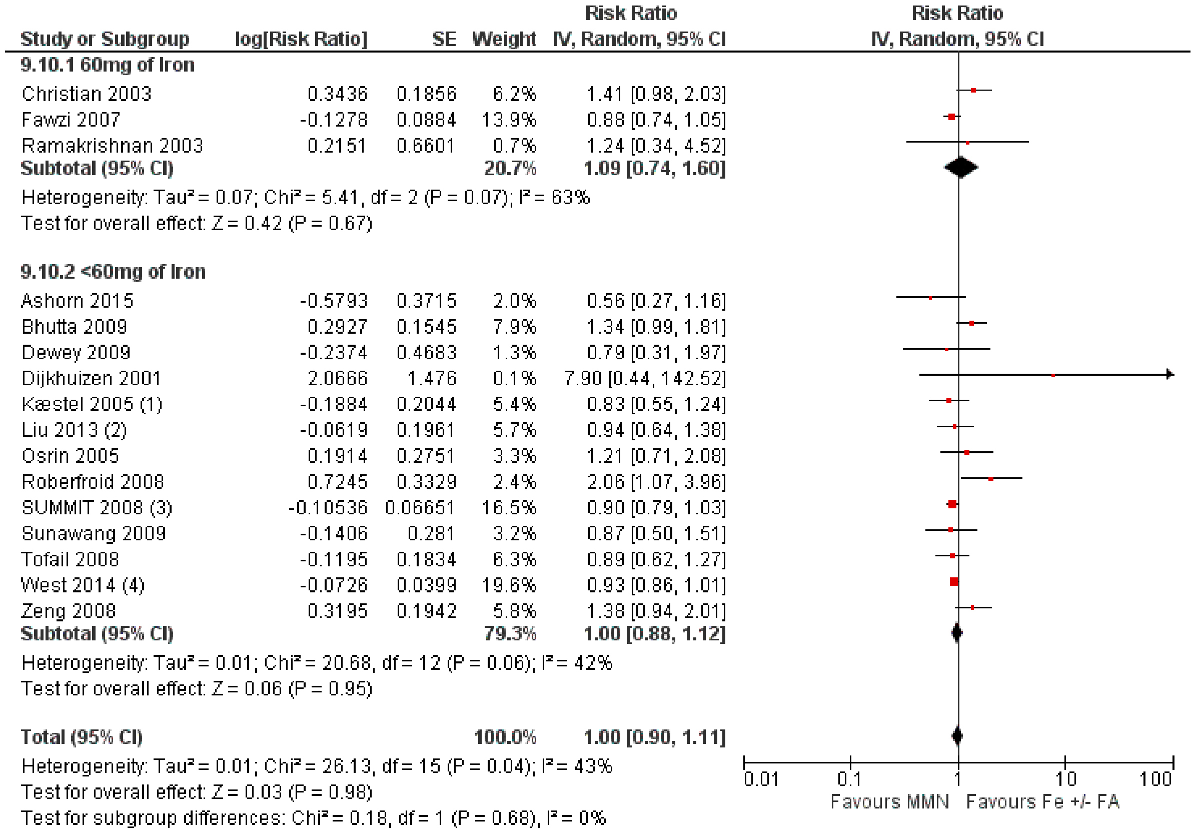

Footnotes

(1) 30mg of Fe

(2) 30mg of Fe

(3) 30mg of Fe

(4) 27mg of Fe

Supplementary Materials

Low Birthweight (Geographical Region)

Study or Subgroup	log[Risk Ratio]	SE	Weight	Risk Ratio IV, Fixed, 95% CI
**9.12.1 Africa**				
Ashorn 2015	0.2738	0.2266	0.4%	1.31 [0.84, 2.05]
Dewey 2009	-0.3603	0.2181	0.5%	0.70 [0.45, 1.07]
Fawzi 2007	-0.1985	0.0807	3.5%	0.82 [0.70, 0.96]
Friis 2004	-0.3011	0.2537	0.4%	0.74 [0.45, 1.22]
Kæstel 2005	-0.1315	0.1921	0.6%	0.88 [0.60, 1.28]
Moore 2012	-0.2341	0.3242	0.2%	0.79 [0.42, 1.49]
Roberfroid 2008	-0.0629	0.1462	1.1%	0.94 [0.71, 1.25]
Saaka 2009	-0.1662	0.2974	0.3%	0.85 [0.47, 1.52]
Zagré 2007	-0.1562	0.1491	1.0%	0.86 [0.64, 1.15]
**Subtotal (95% CI)**			**8.0%**	**0.85 [0.77, 0.95]**
Heterogeneity: Chi² = 5.56, df = 8 (P = 0.70); I² = 0%				
Test for overall effect: Z = 2.95 (P = 0.003)				
**9.12.2 South Asia**				
Bhutta 2009	-0.1985	0.1426	1.1%	0.82 [0.62, 1.08]
Christian 2003	0.0296	0.0861	3.1%	1.03 [0.87, 1.22]
Dijkhuizen 2001	0.4595	0.5854	0.1%	1.58 [0.50, 4.99]
Hafeez 2005	0.3102	0.3756	0.2%	1.36 [0.65, 2.85]
Osrin 2005	-0.2866	0.1167	1.7%	0.75 [0.60, 0.94]
Roth 2018 (MDIG)	0.3667	0.2077	0.5%	1.44 [0.96, 2.17]
SUMMIT 2008	-0.1508	0.0836	3.3%	0.86 [0.73, 1.01]
Sunawang 2009	-0.1682	0.3016	0.3%	0.85 [0.47, 1.53]
Tofail 2008	-0.0992	0.0655	5.3%	0.91 [0.80, 1.03]
West 2014	-0.1278	0.0177	73.2%	0.88 [0.85, 0.91]
**Subtotal (95% CI)**			**88.7%**	**0.89 [0.86, 0.91]**
Heterogeneity: Chi² = 13.59, df = 9 (P = 0.14); I² = 34%				
Test for overall effect: Z = 7.54 (P < 0.00001)				
**9.12.3 Americas**				
Caulfield 1999	-0.0397	0.3368	0.2%	0.96 [0.50, 1.86]
Ramakrishnan 2003	-0.0458	0.2577	0.3%	0.96 [0.58, 1.58]
**Subtotal (95% CI)**			**0.5%**	**0.96 [0.64, 1.43]**
Heterogeneity: Chi² = 0.00, df = 1 (P = 0.99); I² = 0%				
Test for overall effect: Z = 0.21 (P = 0.83)				
**9.12.4 Western Pacific**				
Hanieh 2013	-0.0631	0.3918	0.1%	0.94 [0.44, 2.02]
Huy 2009	-0.982	0.2489	0.4%	0.37 [0.23, 0.61]
Liu 2013	-1.054	0.1282	1.4%	0.35 [0.27, 0.45]
Zeng 2008	-0.1034	0.2013	0.6%	0.90 [0.61, 1.34]
**Subtotal (95% CI)**			**2.5%**	**0.46 [0.38, 0.56]**
Heterogeneity: Chi² = 19.85, df = 3 (P = 0.0002); I² = 85%				
Test for overall effect: Z = 7.97 (P < 0.00001)				
**9.12.5 Eastern Mediterranean**				
Aminisani 2009	-2.5536	1.4599	0.0%	0.08 [0.00, 1.36]
Sorouri 2016	-0.1036	0.3223	0.2%	0.90 [0.48, 1.70]
**Subtotal (95% CI)**			**0.2%**	**0.80 [0.43, 1.49]**
Heterogeneity: Chi² = 2.69, df = 1 (P = 0.10); I² = 63%				
Test for overall effect: Z = 0.69 (P = 0.49)				
**Total (95% CI)**			**100.0%**	**0.87 [0.84, 0.90]**
Heterogeneity: Chi² = 85.93, df = 26 (P < 0.00001); I² = 70%				
Test for overall effect: Z = 9.24 (P < 0.00001)				
Test for subgroup differences: Chi² = 44.24, df = 4 (P < 0.00001), I² = 91.0%				

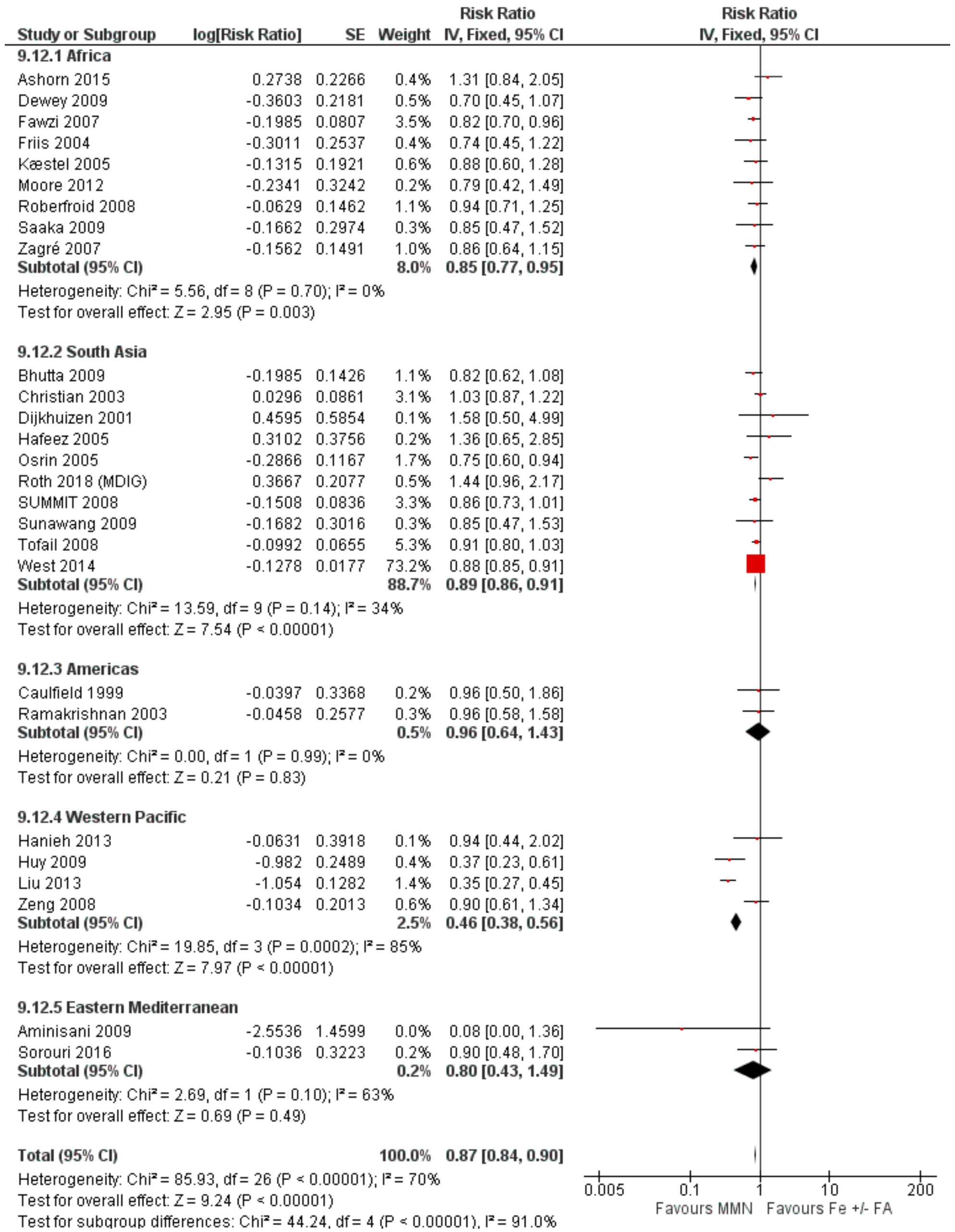

Supplementary Materials

Low Birthweight (Dose of Iron, mg)

Study or Subgroup	log[Risk Ratio]	SE	Weight	Risk Ratio IV, Random, 95% CI
**9.13.1 60mg of Iron**				
Caulfield 1999	-0.0397	0.3368	1.8%	0.96 [0.50, 1.86]
Christian 2003	0.0296	0.0861	7.0%	1.03 [0.87, 1.22]
Fawzi 2007	-0.1985	0.0807	7.1%	0.82 [0.70, 0.96]
Hanieh 2013	-0.0631	0.3918	1.4%	0.94 [0.44, 2.02]
Moore 2012	-0.2341	0.3242	2.0%	0.79 [0.42, 1.49]
Ramakrishnan 2003	-0.0458	0.2577	2.7%	0.96 [0.58, 1.58]
Roth 2018 (MDIG)	0.3667	0.2077	3.6%	1.44 [0.96, 2.17]
**Subtotal (95% CI)**			**25.6%**	**0.96 [0.83, 1.12]**
Heterogeneity: Tau² = 0.01; Chi² = 8.54, df = 6 (P = 0.20); I² = 30%				
Test for overall effect: Z = 0.49 (P = 0.63)				
**9.13.2 <60mg of Iron**				
Aminisani 2009	-2.5536	1.4599	0.1%	0.08 [0.00, 1.36]
Ashorn 2015	0.2738	0.2266	3.2%	1.31 [0.84, 2.05]
Bhutta 2009	-0.1985	0.1426	5.2%	0.82 [0.62, 1.08]
Dewey 2009	-0.3603	0.2181	3.4%	0.70 [0.45, 1.07]
Dijkhuizen 2001	0.4595	0.5854	0.7%	1.58 [0.50, 4.99]
Huy 2009	-0.982	0.2489	2.9%	0.37 [0.23, 0.61]
Kæstel 2005 (1)	-0.1315	0.1921	3.9%	0.88 [0.60, 1.28]
Liu 2013 (2)	-1.054	0.1282	5.6%	0.35 [0.27, 0.45]
Osrin 2005	-0.2866	0.1167	6.0%	0.75 [0.60, 0.94]
Roberfroid 2008	-0.0629	0.1462	5.1%	0.94 [0.71, 1.25]
Saaka 2009 (3)	-0.1662	0.2974	2.2%	0.85 [0.47, 1.52]
Sorouri 2016	-0.1036	0.3223	2.0%	0.90 [0.48, 1.70]
SUMMIT 2008 (4)	-0.1508	0.0836	7.0%	0.86 [0.73, 1.01]
Sunawang 2009	-0.1682	0.3016	2.2%	0.85 [0.47, 1.53]
Tofail 2008	-0.0992	0.0655	7.6%	0.91 [0.80, 1.03]
West 2014 (5)	-0.1278	0.0177	8.5%	0.88 [0.85, 0.91]
Zagré 2007	-0.1562	0.1491	5.0%	0.86 [0.64, 1.15]
Zeng 2008	-0.1034	0.2013	3.7%	0.90 [0.61, 1.34]
**Subtotal (95% CI)**			**74.4%**	**0.79 [0.69, 0.89]**
Heterogeneity: Tau² = 0.04; Chi² = 73.19, df = 17 (P < 0.00001); I² = 77%				
Test for overall effect: Z = 3.67 (P = 0.0002)				
**Total (95% CI)**			**100.0%**	**0.83 [0.75, 0.92]**
Heterogeneity: Tau² = 0.03; Chi² = 84.09, df = 24 (P < 0.00001); I² = 71%				
Test for overall effect: Z = 3.55 (P = 0.0004)				
Test for subgroup differences: Chi² = 3.92, df = 1 (P = 0.05), I² = 74.5%				

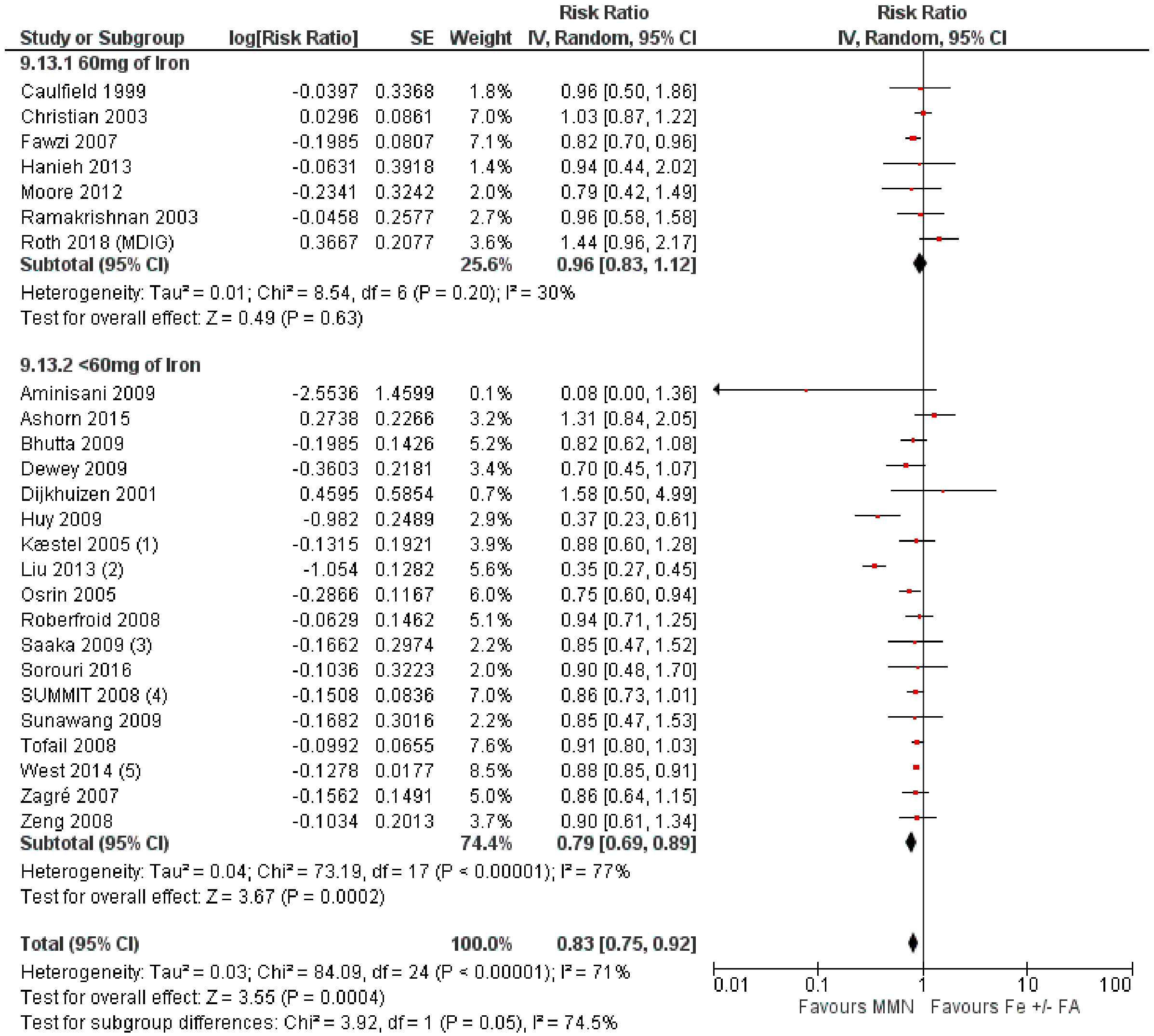

Footnotes

(1) 30mg of Fe

(2) 30mg of Fe

(3) 40mg of Fe

(4) 30mg of Fe

(5) 27mg of Fe

Supplementary Materials

Comparison: Lipid-based Nutrient Supplementation (LNS) vs. MMN Supplementation

Low birthweight

	LNS		MMN			Risk Ratio
Study or Subgroup	Events	Total	Events	Total	Weight	M-H, Random, 95% CI
Ashorn 2015	46	380	51	379	30.5%	0.90 [0.62, 1.31]
Dewey 2009 (1)	27	307	32	318	17.8%	0.87 [0.54, 1.42]
Huybregts 2009	67	523	67	497	42.4%	0.95 [0.69, 1.30]
Moore 2012 (2)	15	159	16	164	9.4%	0.97 [0.49, 1.89]
**Total (95% CI)**		**1369**		**1358**	**100.0%**	**0.92 [0.75, 1.13]**
Total events	155		166			

Heterogeneity: Tau² = 0.00; Chi² = 0.12, df = 3 (P = 0.99); I² = 0%

Test for overall effect: Z = 0.77 (P = 0.44)

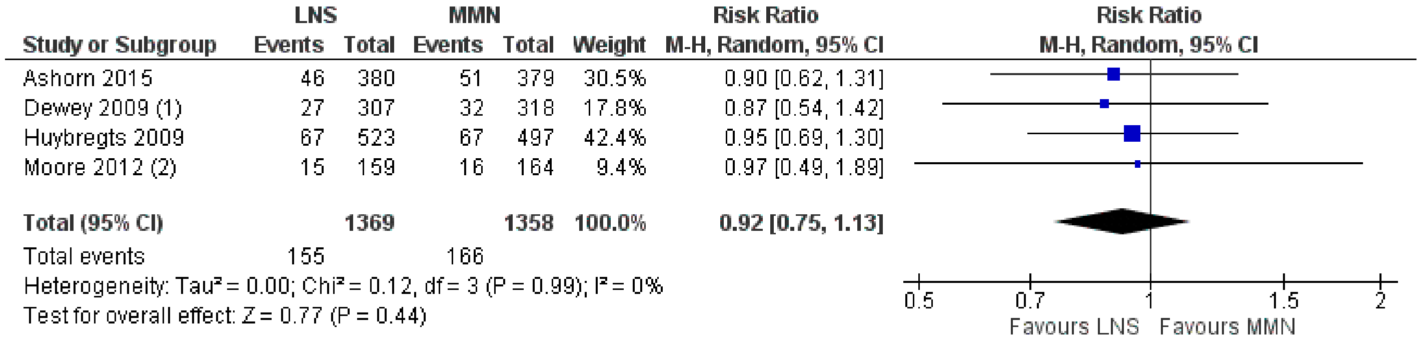

Footnotes

(1) In this paper, under reference Adu-Afarwuah et al. (2015)

(2) In this paper, under reference: Johnson et al. (2017)

Perinatal mortality

	LNS		MMN			Risk Ratio
Study or Subgroup	Events	Total	Events	Total	Weight	M-H, Random, 95% CI
Ashorn 2015 (1)	21	428	14	433	40.9%	1.52 [0.78, 2.94]
Dewey 2009 (2)	5	354	8	354	17.6%	0.63 [0.21, 1.89]
Huybregts 2009	16	606	19	596	41.6%	0.83 [0.43, 1.59]
**Total (95% CI)**		**1388**		**1383**	**100.0%**	**1.01 [0.62, 1.65]**
Total events	42		41			

Heterogeneity: Tau² = 0.04; Chi² = 2.52, df = 2 (P = 0.28); I² = 21%

Test for overall effect: Z = 0.04 (P = 0.97)

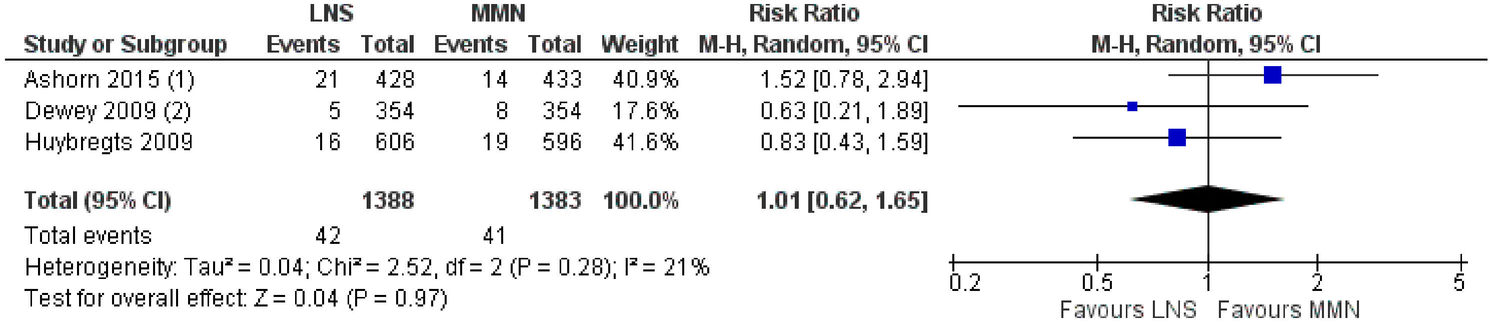

Footnotes

(1) The denominator includes women with a singleton pregnancy for whom the date of abortion or delivery is known

(2) In this paper, under reference Adu-Afarwuah et al. (2015)

Supplementary Materials

Miscarriage

Study or Subgroup	LNS Events	LNS Total	MMN Events	MMN Total	Weight	Risk Ratio M-H, Random, 95% CI
Ashorn 2015	3	428	4	433	10.3%	0.76 [0.17, 3.37]
Dewey 2009 (1)	9	354	9	354	27.5%	1.00 [0.40, 2.49]
Huybregts 2009 (2)	23	655	18	641	62.2%	1.25 [0.68, 2.29]
**Total (95% CI)**		**1437**		**1428**	**100.0%**	**1.12 [0.69, 1.80]**
Total events	35		31			

Heterogeneity: Tau² = 0.00; Chi² = 0.45, df = 2 (P = 0.80); I² = 0%
Test for overall effect: Z = 0.45 (P = 0.65)

Risk Ratio
M-H, Random, 95% CI

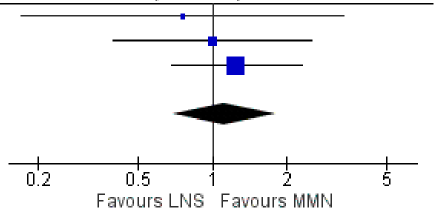

Footnotes
(1) In this paper, under reference Adu-Afarwuah et al. (2015)
(2) *Note: denominator used is different from other two studies. Denominator is total pregnant women assigned to control/intervention...

Neonatal mortality

Study or Subgroup	LNS Events	LNS Total	MMN Events	MMN Total	Weight	Risk Ratio M-H, Random, 95% CI
Ashorn 2015	8	414	13	430	45.7%	0.64 [0.27, 1.53]
Dewey 2009 (1)	3	354	2	354	10.9%	1.50 [0.25, 8.92]
Huybregts 2009	9	594	10	581	43.4%	0.88 [0.36, 2.15]
**Total (95% CI)**		**1362**		**1365**	**100.0%**	**0.81 [0.45, 1.45]**
Total events	20		25			

Heterogeneity: Tau² = 0.00; Chi² = 0.78, df = 2 (P = 0.68); I² = 0%
Test for overall effect: Z = 0.72 (P = 0.47)

Risk Ratio
M-H, Random, 95% CI

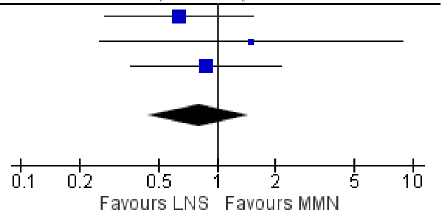

Footnotes
(1) In this paper, under reference Adu-Afarwuah et al. (2015)

Preterm births

Study or Subgroup	LNS Events	LNS Total	MMN Events	MMN Total	Weight	Risk Ratio M-H, Random, 95% CI
Ashorn 2015	39	428	41	433	25.6%	0.96 [0.63, 1.46]
Dewey 2009 (1)	29	308	19	318	14.4%	1.58 [0.90, 2.75]
Huybregts 2009	92	576	79	567	58.0%	1.15 [0.87, 1.51]
Moore 2012 (2)	4	159	3	164	2.0%	1.38 [0.31, 6.05]
**Total (95% CI)**		**1471**		**1482**	**100.0%**	**1.15 [0.93, 1.42]**
Total events	164		142			

Heterogeneity: Tau² = 0.00; Chi² = 1.99, df = 3 (P = 0.58); I² = 0%
Test for overall effect: Z = 1.31 (P = 0.19)

Risk Ratio
M-H, Random, 95% CI

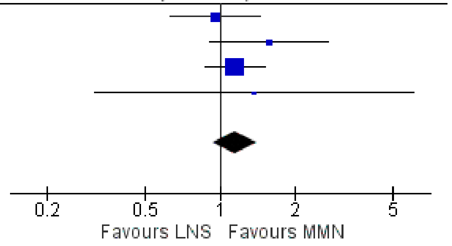

Footnotes
(1) In this paper, under reference Adu-Afarwuah et al. (2015)
(2) In this paper, under reference: Johnson et al. (2017)

Supplementary Materials

Stillbirths

	LNS		MMN			Risk Ratio	Risk Ratio
**Study or Subgroup**	**Events**	**Total**	**Events**	**Total**	**Weight**	**M-H, Random, 95% CI**	**M-H, Random, 95% CI**
Ashorn 2015	2	290	14	281	29.3%	0.14 [0.03, 0.60]	
Dewey 2009 (1)	3	354	7	354	31.1%	0.43 [0.11, 1.64]	
Huybregts 2009	15	596	12	606	39.6%	1.27 [0.60, 2.69]	
**Total (95% CI)**		**1240**		**1241**	**100.0%**	**0.47 [0.12, 1.81]**	
Total events	20		33				

Heterogeneity: Tau² = 1.03; Chi² = 7.85, df = 2 (P = 0.02); I² = 75%

Test for overall effect: Z = 1.09 (P = 0.27)

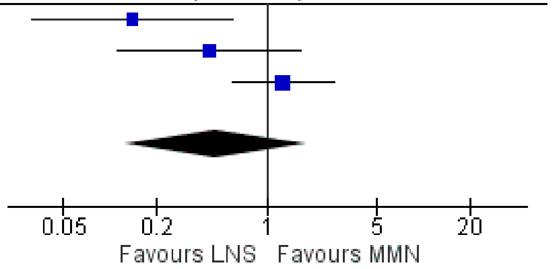

Footnotes

(1) In this paper, under reference Adu-Afarwuah et al. (2015)
